# Regional nutrient decrease drove redox stabilisation and metazoan diversification in the late Ediacaran Nama Group, Namibia

**DOI:** 10.1038/s41598-020-59335-2

**Published:** 2020-02-10

**Authors:** F. T. Bowyer, A. J. Shore, R. A. Wood, L. J. Alcott, A. L. Thomas, I. B. Butler, A. Curtis, S. Hainanan, S. Curtis-Walcott, A. M. Penny, S. W. Poulton

**Affiliations:** 1 University of Edinburgh, School of GeoSciences, James Hutton Road, Edinburgh, EH9 3FE UK; 2 University of Leeds, School of Earth and Environment, Leeds, LS2 9JT UK; 3 Ministry of Mines and Energy, 6 Aviation Road, Private Bag, 13297 Windhoek, Namibia; 4 Department of Physics, Imperial College, London, SW7 2AZ UK; 5 Finnish Museum of Natural History, University of Helsinki, Jyrängöntie 2, 00560 Helsinki, Finland; 6 Present Address: University of Leeds, School of Earth and Environment, Leeds, LS2 9JT UK

**Keywords:** Element cycles, Geochemistry, Palaeontology

## Abstract

The late Ediacaran witnessed an increase in metazoan diversity and ecological complexity, marking the inception of the Cambrian Explosion. To constrain the drivers of this diversification, we combine redox and nutrient data for two shelf transects, with an inventory of biotic diversity and distribution from the Nama Group, Namibia (~550 to ~538 Million years ago; Ma). Unstable marine redox conditions characterised all water depths in inner to outer ramp settings from ~550 to 547 Ma, when the first skeletal metazoans appeared. However, a marked deepening of the redoxcline and a reduced frequency of anoxic incursions onto the inner to mid-ramp is recorded from ~547 Ma onwards, with full ventilation of the outer ramp by ~542 Ma. Phosphorus speciation data show that, whilst anoxic ferruginous conditions were initially conducive to the drawdown of bioavailable phosphorus, they also permitted a limited degree of phosphorus recycling back to the water column. A long-term decrease in nutrient delivery from continental weathering, coupled with a possible decrease in upwelling, led to the gradual ventilation of the Nama Group basins. This, in turn, further decreased anoxic recycling of bioavailable phosphorus to the water column, promoting the development of stable oxic conditions and the radiation of new mobile taxa.

## Introduction

The earliest candidate metazoan body fossils appear in the fossil record in the Ediacaran at ~571 Ma^1^, with the first uncontroversial surface traces created by motile organisms known from ~560 Ma^2^, followed by skeletal hardparts at ~550 Ma^3^. The Ediacaran-Cambrian boundary, as currently defined, is placed at ~541 Ma, and by ~520 Ma most major phyla had appeared, marking the end of the Cambrian Explosion. While the oxygen requirements of Ediacaran metazoans are unknown, it has been widely suggested that an increase in the dissolved oxygen concentration of marine environments enabled the rise of metabolically active ecologies, including large body size, bilaterian burrowing and biomineralisation^4^. The key to clarifying the relationships between redox and early metazoan diversification and complexity lies in high resolution studies that integrate local redox with biotic distribution^5,6^.

The late Ediacaran (c. 580–541 Ma) oceans were likely characterised by low oxygen levels^7^, and individual basins of the Ediacaran and Cambrian that were openly connected to the global ocean exhibit continued regional redox heterogeneity on million year timescales^5,8,9^. Whilst some mid-shelf and lower slope environments record a transition to permanent, stable oxia as early as ~577 Ma, other lower slope settings were characterised by continued anoxic and ferruginous (free Fe(II)) or even euxinic (free aqueous hydrogen sulfide) water column conditions (see review ref. ^9^). Indeed, ferruginous water column conditions have been recorded from shallow marine sediments deposited above fair weather wave base in the final 10 million years of the Ediacaran^5,6^. The observed variability in the local redox of late Ediacaran and early Cambrian marine environments may correspond to the relative distance from, and spatial extent of, oxygen minimum zones (OMZs) that developed in response to local productivity and resultant organic matter remineralization^10^. Spatially variable rates of primary production, in turn, are likely to reflect the provision of limiting nutrients, in particular phosphorus (P), which is commonly considered the ultimate limiting nutrient on geological timescales^11^.

Here, we utilise iron (Fe) and P speciation data across two shelf transects to create a 4D reconstruction of local-scale redox and nutrient cycling dynamics in the Ediacaran to early Cambrian Nama Group, Namibia (~550–538 Ma). Previous palaeoredox studies of the Nama Group have demonstrated the limitation imposed on skeletal metazoans by low oxygen waters^5,6^. By contrast, potentially detrimental incursions of ferruginous waters into the shallowest marine settings that were host to abundant soft-bodied organisms and trace makers, have been largely overlooked in geochemical studies as a consequence of fossil preservation within coarse siliciclastics that are not well-suited to geochemical redox analyses. However, the well-defined sequence stratigraphic framework of the Nama Group enables analysis of down-dip shales deposited time-equivalent to shallow, fossiliferous sandstone layers, and construction of variable-depth geochemical transects. This enables the relative position of the redoxcline to be tracked through time, as well as quantification of the incursion frequency of anoxic waters onto the shallow, inner ramp. We combine this approach with a recent compilation of biotic diversity and distribution throughout the Nama Group in order to clarify the relationship between local redox stability and benthic colonisation.

## Geological Setting: The Nama Group, Namibia

**Figure 1:**
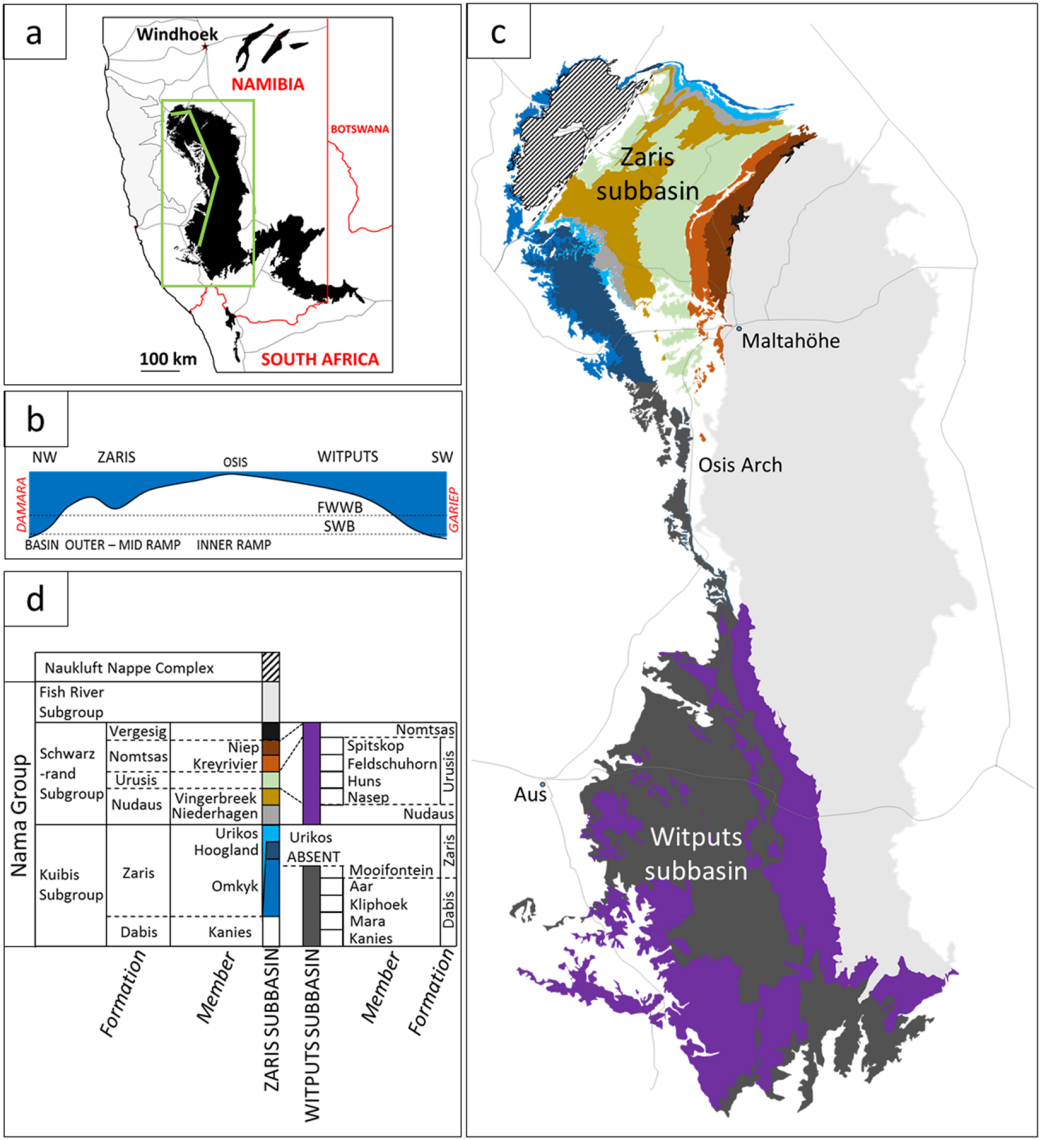
Geological setting of the Nama Group, Namibia. (**a**) Regional map showing outcrop of Nama Group with green box delineating study area and line of cross section in (**b**) through basins in Namibia. (**b**) Schematic cross section of palaeobathymetry in the Kuibis Subgroup. (**c**) Geological map of Nama Group, Namibia. Redrawn using 1:1000000 map and 1:250000 map of Mariental (2416), Geological Survey of Namibia, Ministry of Mines and Energy. (**d**) Stratigraphic key for geological map shown in (c).

The Nama Group comprises a mixed carbonate and siliciclastic ramp succession deposited within a foreland basin on the Kalahari Craton during convergence along two orogenic belts; the Damara to the north and the Gariep to the southwest^12^ (Fig. 1a–d). Zircons within silicified air-fall tuff deposits yield ID-TIMS U-Pb ages that constrain deposition between approximately 547 and 538 Ma^13–15^. The lowermost dated ash layer occurs at a maximum of >500 m above the basal Nama unconformity, and an approximate age of 550–553 Ma for the base of the Nama Group has been extrapolated from inferred rates of sedimentation^16^, suggesting that deposition of the Kuibis and Schwarzrand subgroups persisted for ~12 Myr.

Sequence stratigraphy and δ^13^C_carb_ chemostratigraphy are well established^16–19^, and detailed mapping of individual members enables parasequence level correlation across distances of up to 100 km^19^. Sediments were deposited in two sub-basins, the Zaris and the Witputs, that deepened to the northwest and southwest, respectively, with distance from a basement forebulge, the Osis Arch (Fig. 1b)^12^. The Nama Group is divided into the lower Kuibis and upper Schwarzrand Subgroups (Fig. 1c), which are further subdivided based on regional facies and sequence stratigraphic mapping^12,18,19^. During deposition of the Kuibis Subgroup, the two sub-basins show an independent stratigraphy imposed by the Osis Arch^16,18^ (Fig. 1b). Sub-basin connectivity increased due to transgression during deposition of the Schwarzrand Subgroup and gradual infill of the Zaris sub-basin^12,18^. Connection to the open ocean throughout Nama Group deposition is corroborated by strontium isotopic data^20^, normal marine rare earth element profiles^6^, and carbon isotopic data that are globally correlative. A gradual, basin-wide transition from negative to positive carbonate carbon isotope values may record the local expression of recovery from the presumed-global Shuram excursion (however this remains uncertain), but overlying strata are not known to record (or may predate) the basal Cambrian negative carbon isotope excursion (BACE)^5,16^.

Almost 90 years of palaeontological study in the Nama Group has uncovered a diverse fossil record including the soft-bodied macro-organisms *Pteridinium*, *Rangea*, *Ernietta*, *Nasepia*, *Namalia*, and *Swartpuntia*^21–23^. Microbially-induced sedimentary structures (MISS), stromatolites, thrombolites and organic walled microfossils, including leiosphaerid acritarchs are also described^24^. Carbonate rocks host the skeletal taxa *Namacalathus*, *Cloudina*, and *Namapoikia*^3,25^. The Nama Group also hosts a rich suite of ichnofossils, including the first representatives of treptichnids^26^, and first traces of extensive, but localised, sediment bulldozing^27^.

Previous geochemical studies utilising Fe-S-C systematics and rare earth element profiles have revealed a highly heterogeneous palaeoredox environment for the Kuibis Subgroup, and constrain deposition under variably oxic, dysoxic manganous, and anoxic ferruginous waters in inner to outer-ramp facies^5–7^. Repeated incursions of ferruginous anoxia into inner shelf environments are considered to reflect changes in the position of the redoxcline, which was, in large part, controlled by changing relative sea level^5,9^.

## Methods

Full details of all analytical procedures, including the full dataset, are provided in the Supplementary Information, which includes additional palaeontological information, GPS coordinates and stratigraphic logs. A composite δ^13^C_carb_ curve tied to major sequence boundaries was constructed using sequence stratigraphy, in order to correlate sample position in the Kuibis subgroup across the Osis Arch (see Supplementary Information). Compiled iron and phosphorus speciation data are placed within this framework and partitioned into four broad settings (incorporating inner, mid and outer ramp) based on sedimentary depth indicators and proximity to the Osis Arch^5,7,12,18^ (see Supplementary Information for discussion).

Iron speciation^28^ is the primary palaeoredox proxy employed in this study, and the compilation incorporates new and previously published data from shales (n = 218) and carbonates (n = 104). A sequential leach separates iron carbonates (Fe_carb_), oxides (Fe_ox_) and magnetite (Fe_mag_), which, in addition to pyrite (Fe_py_), operationally defines an iron pool that is considered highly reactive (Fe_HR_) to reduction under anoxic conditions^29,30^. Anoxic water column conditions commonly promote enrichments in Fe_HR_ relative to total iron (Fe_T_) via the water column precipitation of unsulfidized Fe_HR_ minerals in ferruginous basins and Fe sulfides in euxinic settings^31^. Extensive calibration in modern and ancient settings suggests that Fe_HR_/Fe_T_ < 0.22 provides a robust indication of oxic water column depositional conditions, while ratios >0.38 suggest anoxic deposition^31^. Values between 0.22 to 0.38 are considered equivocal due to the potential for muted Fe_HR_/Fe_T_ under anoxic conditions due to rapid deposition, or diagenetic transformation of unsulfidized Fe_HR_ to poorly reactive sheet silicates^32^. In the latter case, further insight can be gained by considering total Fe/Al ratios, which are not affected by diagenetic modification of individual Fe pools. Calibration studies suggest that Fe/Al > ~0.66 indicates deposition from an anoxic water column^33,34^. The proportion of Fe_HR_ contributed by Fe_py_ distinguishes between ferruginous and euxinic conditions, with Fe_py_/Fe_HR_ > 0.7–0.8 indicative of a euxinic water column^32^.

The majority of the compiled iron speciation data (~70%) relate to shales interbedded between carbonates. For carbonates, discerning palaeoredox conditions can be complicated by their potential sensitivity to external inputs of Fe_HR_ during diagenesis, which is particularly the case for carbonates with a low overall Fe content^5,34^. However, a detailed calibration suggests that Fe-speciation and Fe/Al results tend to be robust when Fe_T_ is >0.5 wt%, providing the sediments have not been subject to deep burial dolomitization^34^. All carbonate iron speciation data in this compilation have been published previously^5^ and comply with the requisite minimum total Fe concentration (Fe_T_) of 0.5 wt%^34^. Furthermore, there is no evidence for deep burial dolomitization in this succession, and an early origin for Nama Group dolomite is attributed either to evaporitic conditions^35^, akin to modern dolomitizing environments, or to early diagenetic dolomitization of Fe-rich, high magnesium calcite precursor cements, precipitated in pore fluids that were openly connected to the overlying ferruginous water column^36^. Importantly, Fe-speciation data from interbedded shale and carbonate samples of the Nama Group generally give consistent interpretations, suggesting that their redox interpretation is robust^5^, but where differences exist we place emphasis on the shale record.

We employ the chemical index of alteration (CIA)^37^ to assess changes in the degree of chemical weathering (see Supplementary Information for further discussion). Chemical weathering plays a major role in controlling the mineralogical composition of fine-grained siliciclastic rocks and is therefore a key metric to consider when evaluating compositional changes in their major element distribution. All shale samples for which new data are presented in this study (n = 113) were subjected to a total digestion for the analysis of major element (Al, Ca, Fe, K, Mn, Na, P, Ti) concentrations.

Finally, a selection of representative shale samples (n = 48) across all depositional settings and time intervals were analysed for total organic carbon (TOC) and phosphorus speciation using a modified version of the SEDEX extraction scheme^38^ (see Supplementary Information; note that carbonate samples were avoided due to the potential for additional uptake of P in the carbonate lattice, which may skew comparisons to average shale compositions). This technique quantifies the proportion of total P (P_Tot_) associated with Fe (oxyhydr)oxide minerals (P_Fe_), organic matter (P_org_), authigenic carbonate fluorapatite, biogenic apatite and CaCO_3_ (P_auth_), and detrital apatite (P_det_)^38^. Reactive P (P_Fe_ + P_org_ + P_auth_) defines a pool that may potentially be bioavailable and mobile during deposition and early diagenesis^39^, in contrast to detrital P.

## Results

**Figure 2:**
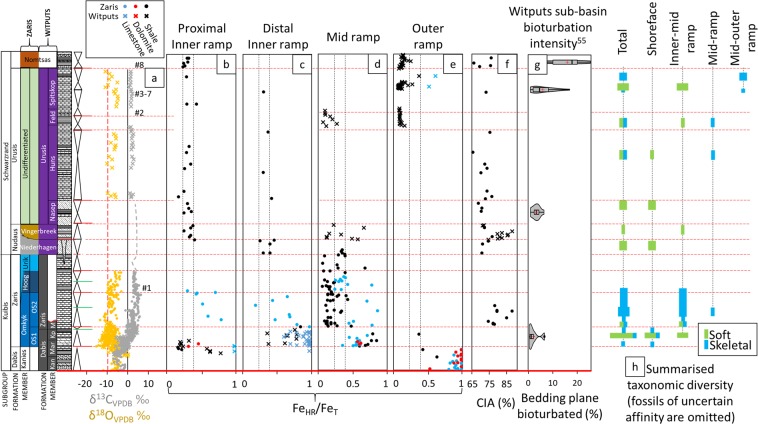
Carbonate C isotopes, redox and fossil occurrences in the Nama Group, Namibia, following reference section of (16). (**a**) Composite δ^13^C_carb_ profile (see Supplementary Information). Hashed numbers correspond to the position of dated ash beds: #1, 547.36 ± 0.65 Ma^14^; #2, 542.68 ± 2.80 Ma^13,52^; #3–7, 540.095 ± 0.099 Ma to 538.99 ± 0.21 Ma^15^; #8, 538.58 ± 0.19 Ma^15^. (**b-e**) Compiled Fe_HR_/Fe_T_ data, separated according to relative depth from inner to outer ramp. Anoxic water column conditions are indicated by Fe_HR_/Fe_T_ > 0.38, ratios of Fe_py_/Fe_HR_ are ubiquitously <0.3, characteristic of a ferruginous water column and consequently, ratios of Fe_py_/Fe_HR_ are omitted. (**f**) Chemical index of alteration (CIA). (**g**) Violin plots of bioturbation intensity for the Witputs sub-basin after (55). (**h**) Summarised taxonomic diversity of published Nama Group body fossil occurrences (see Figure S12 and Table S1 for details).

In the Kuibis Subgroup (~550 to < 547 Ma), carbonate deposits of the Kanies, Mara and lower Omkyk members have been suggested to record recovery from the Shuram C isotope excursion, however the global nature and geochronology of the Shuram excursion remain uncertain (Fig. 2a; see Methods for details of geochemical techniques and their interpretive framework, and Supplementary Information for all data)^5^. At this time, outer ramp deposits in the Zaris sub-basin have persistently elevated Fe_HR_/Fe_T_ > 0.52 (Fe/Al = 0.7–7; Fig. 2e)^5^, whilst samples deposited above fair weather wave base on the shallow inner ramp near the Osis Arch (Fig. 2b) record a transition from initially high (0.58–1.00), to low (0.13–0.19) Fe_HR_/Fe_T_. Meanwhile, distal inner ramp and mid ramp deposits (Fig. 2c,d) continue to record elevated Fe_HR_/Fe_T_ (0.35–1.00) throughout the lower Omkyk Member (and equivalent Mara and Kliphoek members) in both sub-basins. Where Fe_HR_/Fe_T_ is > 0.38, ratios of Fe_py_/Fe_HR_ are ubiquitously <0.3 in samples of the Nama Group, implying anoxic, ferruginous conditions (Figure S11). Mid-ramp shales of the Upper Omkyk Member dominantly yield low Fe_HR_/Fe_T_ (<0.22; with mean Fe/Al = 0.58), however, limestone samples of the inner to mid-ramp, and occasional shales of the mid-ramp, show elevated Fe_HR_/Fe_T_ and Fe/Al.

Strata correlative to the Upper Omkyk and Hoogland members are absent in the Witputs sub-basin^16^, and shales were instead sampled from the mid-ramp Vingerbreek Member of the Nudaus Formation (Schwarzrand Subgroup, <547‒ > 545 Ma, Fig. 2d). Here, shales with Fe_HR_/Fe_T_ < 0.22 (Fe/Al = 0.54–0.73) are interbedded with shales characterised by Fe_HR_/Fe_T_ > 0.38 (Fe/Al = 0.80–1.16) and the occurrence of ironstones (Fe_T_ up to 29.8 wt%).

In the Zaris sub-basin, samples of the Schwarzrand Subgroup (<547‒ ~538 Ma) have relatively invariant Fe_HR_/Fe_T_ values (Fig. 2b,c), largely confined between the calibrated threshold ratios of 0.22 and 0.38 (Fe/Al = 0.41–0.78, mean = 0.58), challenging an unequivocal palaeoredox interpretation. Superimposed upon the iron speciation record, however, is a notable positive trend in Mn/Al and Mn/Fe, reaching an apex in the Urusis Formation (Figure S11) and culminating in values typical for Phanerozoic shale^40^. More distal deposits of the upper Schwarzrand Subgroup on the mid – outer ramp in the Witputs sub-basin (Fig. 2d,e) are characterised by Fe_HR_/Fe_T_ between 0.03 and 0.35 (mean = 0.09, n = 84), with the exception of two carbonate samples that are enriched in Fe_HR_ (Fe_HR_/Fe_T_ = 0.51 and 0.61).

The chemical index of alteration falls between 59% and 88%, with the highest values confined to the Kuibis Subgroup and the Nudaus Formation (Fig. 2f). The highest CIA values are observed deeper in the succession, in samples of the Upper Omkyk Member of the Zaris sub-basin and the Vingerbreek Member of the Witputs sub-basin, with a provenance from the Kalahari craton to the present east^12^. These high values give way to a narrower range and lower average value in samples of the Schwarzrand Subgroup of the Zaris sub-basin, which were initially a product of sediment input from the north, followed by supply from the east (upper Schwarzrand Subgroup)^12^. The evolving CIA values from samples derived from the east thus support an overall change in weathering intensity, rather than specific changes in lithology.

**Figure 3:**
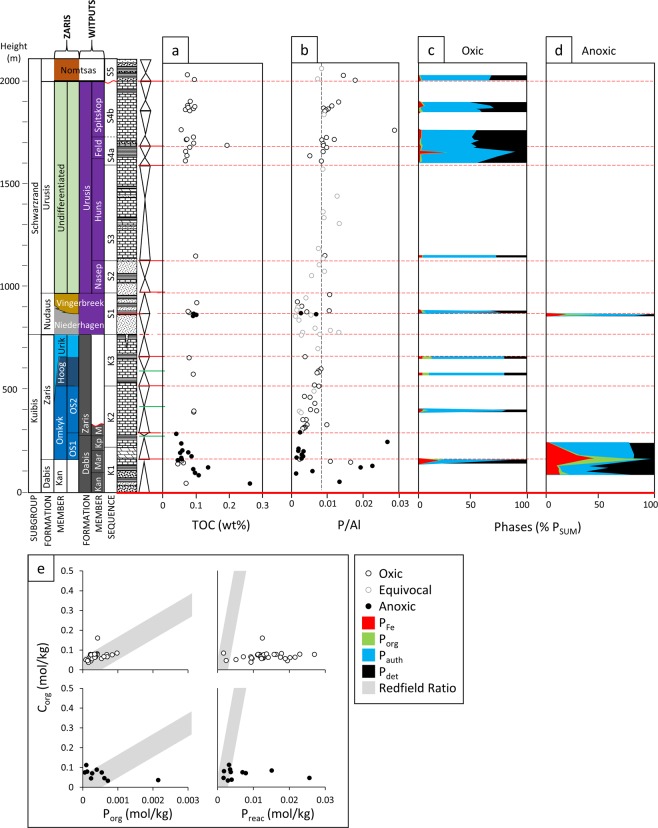
Elemental data of shale samples from the Nama Group, Namibia, differentiated according to redox (based on Fe-speciation). (**a**) TOC, (**b**) The ratio of total phosphorus (P_Tot_) to aluminium (Al), and P phase proportions in (**c**) oxic and (**d**) anoxic samples. (**e**) Cross-plots show (C:P)_org_ and C_org_:P_reac_.

All shale samples have TOC < 0.3 wt%, with similar concentrations for samples deposited beneath oxic and anoxic bottom waters (Fig. 3a). While P/Al ratios scatter above and below average shale values for both oxic and anoxic samples (Fig. 3b), there is distinct P phase partitioning between these redox conditions (Fig. 3c,d). Oxic samples have low P_Fe_ (mean = 2.6% of P_Tot_) and low P_org_ (mean = 1.9% of P_Tot_), with P_auth_ (mean = 52.3% of P_Tot_) and P_det_ (mean = 34.3% of P_Tot_) being the dominant pools. By contrast, ferruginous samples comprise relatively more P_Fe_ (mean = 16.4% of P_Tot_) and P_org_ (mean = 13.0% of P_Tot_), whilst the contributions of P_auth_ (mean = 36.9% of P_Tot_) and P_det_ (mean = 26.8% of P_Tot_) are relatively diminished (but still significant).

## Discussion

Fe-speciation data show pronounced water column redox stratification during deposition of the Kuibis Subgroup^5^. Marine transgression during deposition of the Mara, Kliphoek and lower Omkyk members resulted in basin-wide shoaling of the redoxcline, represented by dominantly anoxic ferruginous deeper and shallower (above fair weather wave base) waters (Fig. 2b,c)^5,6^. The data then show more frequent development of oxygenated conditions through the upper Kuibis Subgroup and the lowermost Schwarzrand Subgroup in both shallower and deeper water. However, short-lived anoxic intervals were a common feature of mid-ramp environments. After this, the inner ramp succession was dominantly deposited under oxic water column conditions, while mid and outer ramp sections were also oxygenated, at least by deposition of the upper Schwarzrand Subgroup. Furthermore, the combination of low and stable Fe_HR_/Fe_T_ and increasing Mn/Fe (Figure S11) suggest a progressive increase in oxygenation during deposition of the Urusis Formation, culminating in conditions conducive to widespread Mn oxidation. Fossil evidence for habitation by trace making organisms occurs in all stable oxic settings from the Nudaus Formation onwards (see Supplementary Information).

Phosphorus systematics provide insight into nutrient cycling as the redox state of the water column developed in the Nama Group. In particular, consideration of P speciation and P/Al ratios helps to constrain potential productivity feedbacks arising from changes in redox conditions^41–43^. Upwelling of ferruginous deep waters may promote P drawdown in association with iron minerals, thus potentially resulting in elevated P/Al and a negative productivity feedback^44^. By contrast, P drawdown in association with iron minerals would be expected to be less significant under oxic water column conditions. However, whilst some ferruginous samples do show P enrichment relative to average shale (Fig. 3b), the majority are depleted, and there is no systematic difference in relation to oxic samples.

Low average P/Al in ferruginous samples of the Kuibis Subgroup may be a consequence of either generally low phosphate availability in the water column, or P recycling from sediments back to the water column. The cycling of P in sediments is largely contingent upon the redox state of the porewaters and water column, with sulfidic conditions being particularly conducive to P recycling^41^. Anaerobic organic matter remineralisation results in the preferential release of P, giving high C_org_/P_org_ ratios relative to the Redfield ratio^45^, while reductive dissolution of Fe (oxyhydr)oxide minerals also releases P to solution^46^. The P released by these processes may undergo ‘sink switching’ to authigenic phases such as carbonate fluorapatite^43^ or vivianite^47^, or may be re-adsorbed to Fe (oxyhydr)oxide minerals where they persist^48^. However, dissolved P may also be recycled back to the water column, particularly under anoxic conditions, potentially promoting a positive productivity feedback^41^.

Our redox data suggest limited sulfide production during diagenesis under both oxic and ferruginous conditions (giving very low Fe_py_/Fe_HR_ ratios; see Supplementary Information). Such conditions would be expected to limit P recycling from the sediment, particularly under oxic conditions (where anaerobic organic matter degradation and the reductive dissolution of Fe (oxyhydr)oxide minerals are restricted), and this can be tested by considering C_org_/P_org_ and C_org_/P_reac_ ratios relative to the Redfield ratio^47^. In oxic samples of the Nama Group, C_org_/P_org_ ratios (Fig. 3e) cluster around the canonical Redfield ratio (106:1). This suggests little preferential release of P from organic matter, which would be consistent with limited anaerobic organic matter remineralization under these low TOC conditions (Fig. 3a). Relatively low C_org_/P_reac_ ratios, coupled with a high proportion of authigenic P (Fig. 3c–e), suggests that the P released from oxic organic matter degradation and the reductive dissolution of Fe (oxyhydr)oxides deeper in the sediment was subsequently fixed in the sediment via ‘sink-switching’, with no evidence for recycling back to the water column. Nevertheless, C_org_/P_org_ ratios at the Redfield ratio argue against chronically nutrient limited productivity (which may raise primary C_org_/P_org_ values to as high as ~600^49^), and instead our data suggest that the oxic Nama basin experienced rates of productivity typical of oxic marine settings.

Some ferruginous samples have elevated (above the Redfield ratio) C_org_/P_org_ ratios, suggesting more effective anaerobic organic matter remineralization relative to oxic samples, as would be expected beneath an anoxic water column. C_org_/P_reac_ ratios are at or below the Redfield ratio, which suggests significant fixation of P in the sediment following draw down with Fe (oxyhydr)oxide minerals. However, despite this enhanced draw down mechanism, the majority of anoxic samples have significantly higher C_org_/P_reac_ ratios relative to oxic samples, which implies a degree of recycling of P back to the water column under ferruginous conditions, with the potential to stimulate a relative increase in productivity^41^.

A specific complication in P speciation analyses of ancient sedimentary rocks involves the post-depositional recrystallisation of authigenic apatite during burial diagenesis^50^. The modified SEDEX protocol employs a sequential chemical extraction which targets P_auth_ (using 1 M Na acetate buffered to pH 4.0) prior to extraction of P_det_ (using 1 M HCl) and as such, any decrease in the solubility of primary P_auth_ as a consequence of burial diagenesis, will be represented by potential transfer of P_auth_ to the P_det_ pool^38^. It has been noted that the detrital P content of modern continental margin sediments is 186 ± 21 ppm^43^, whereas modern oligotrophic settings are characterised by P_det_ in the range 62–310 ppm^51^. Samples from the Nama Group have P_det_ concentrations in the range 4.2–536.4 ppm (mean 208.7 ppm), with maximum values significantly greater than average P_det_ of modern shelf environments. This may suggest that a portion of extracted P_det_ represents burial recrystallisation of initially authigenic P. There is no significant correlation between P_reac_ (as a percentage of P_Tot_) and Al (r^2^ = 0.077, Figure S2c), implying negligible contamination of the P_auth_ pool by P_det_. Consequently, concentrations of P_auth_ (and by extension summed P_reac_) likely represent minimum values, whilst those of P_det_ represent maximum values (see Supplementary Information for further information).

The combined redox proxy data suggest more oxidising water column conditions in the Nama Group basins (above that required to fully oxidise Fe(II)) in inner to mid ramp environments by the time of deposition of the lower Schwarzrand Subgroup, commencing at ~547 Ma (Fig. 2)^14^. The data also support a further progressive increase in oxygenation of the inner ramp to levels sufficient for Mn oxidation by the upper Urusis Formation at ~542–540 Ma^13,15,52^. Whilst outer ramp sections are sparse in the Nama Group, the available data show that ventilation of the outer ramp occurred, at the latest, by deposition of the Feldschuhhorn Member at ~542 Ma^13,52^.

**Figure 4:**
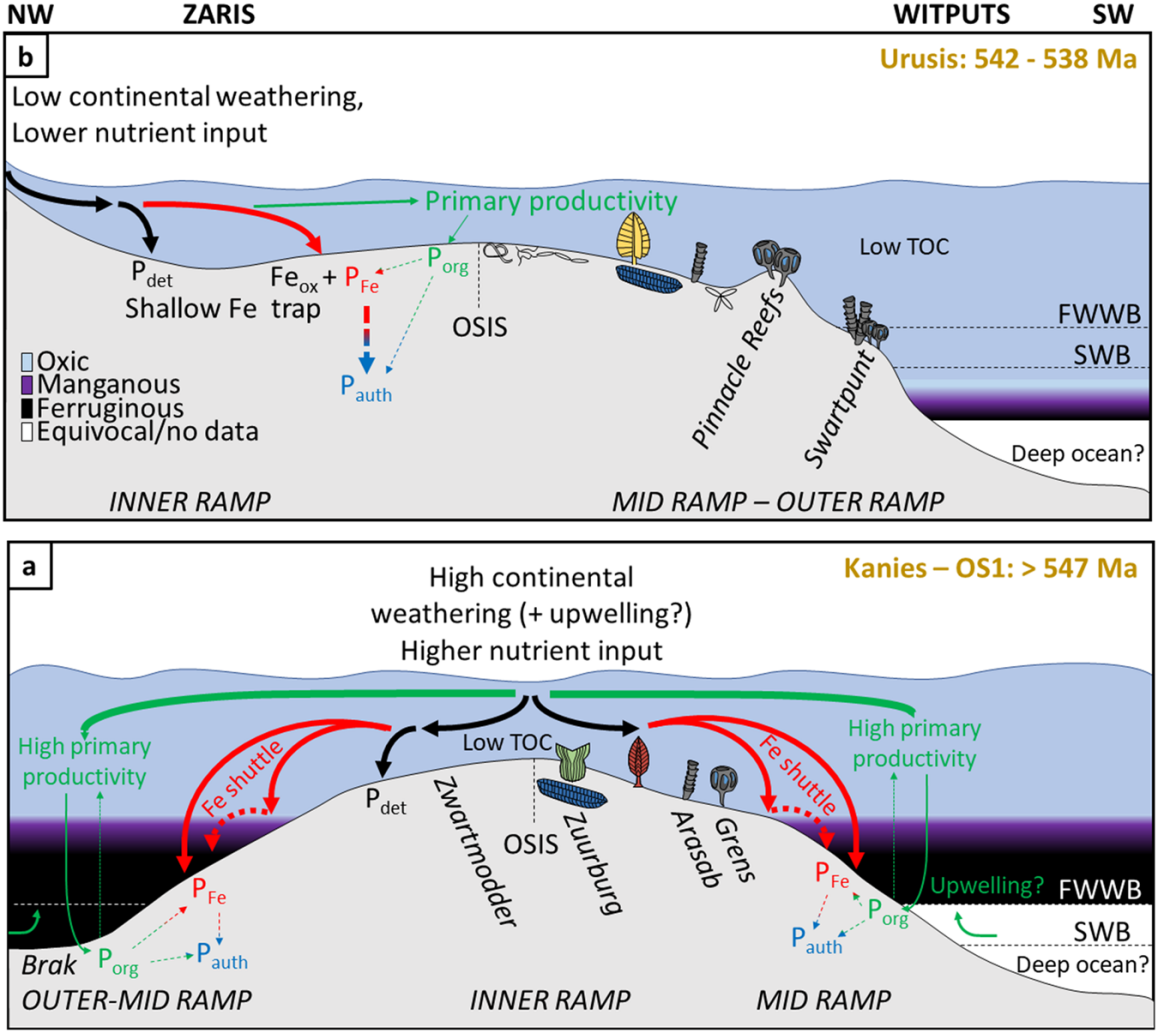
Proposed model for the co-evolution of redox, P burial mechanisms, and evolution of metazoans in the (**a**) lower Kuibis Subgroup (~550–547 Ma), and (**b**) upper Schwarzrand Subgroup (540–538 Ma). Dashed straight arrows denote either sedimentary P diffusion to the water column under ferruginous water column conditions or post-depositional ‘sink-switching’ of P.

What then, caused the progressive ventilation of the Nama basins at ~547 Ma? Fig. 2f shows available data of the chemical index of alteration through the Nama succession. The extent of chemical weathering is dependent upon factors including tectonics and regional climate, and affects the regional supply of nutrients from the continent and the maturity of terrigenous clays^37^. High average CIA values are a dominant feature of the Kuibis Subgroup, and argue for a high degree of chemical weathering, potentially mediated by hot and humid regional climatic conditions^37^. This is followed by a shift, in the Zaris sub-basin, to lower average values that may record a corresponding transition to cooler and drier conditions. There is a distinct peak in CIA that relates to shales of the Vingerbreek Member from the Witputs sub-basin which were deposited atop a sub-basin scale unconformity attributed to a putative short-lived regional glaciation^53^. This peak reflects sediment transport from a source area to the east on the Kalahari Craton and is coeval with a short-lived return to local water column redox stratification and ironstone deposition (see Supplementary Information, section 3d). The overall decrease in the CIA up-section, coupled with the observed covariation with changes in dominant water column redox conditions, point towards a driving role for changes in continentally derived nutrient influx to the basin through time. In this scenario, initial transgression and a high degree of chemical weathering led to deposition of sediments and delivery of nutrients from the Kalahari Craton to the east of the Nama sub-basins. At this time, nutrient input may have been supplemented by a degree of upwelling from the openly connected Brazilides ocean to the west, leading to elevated regional productivity and anoxia (Figs. 4a, S13). However, gradual closure of the Brazilides^54^ ocean likely stifled upwelling in this region, forcing the system to primarily depend upon nutrients supplied from continental weathering. The degree of chemical weathering and associated nutrient input decreased in the Schwarzrand Subgroup, possibly associated with regional climatic change^53^, which would have reduced primary production and hence the extent and maintenance of water column anoxia. The Fe and P speciation data provide insight into the dynamic, regional burial and recycling of the major limiting nutrient P under both ferruginous and oxic conditions^11^. The shallow redoxcline evident during deposition of the lower Kuibis Subgroup readily facilitated the anaerobic degradation of organic matter (and also dissimilatory Fe reduction) in sediments and likely resulted in a small degree of P recycling back to the water column, thus fuelling local productivity (Fig. 4a). However, ferruginous conditions also promoted the removal of reactive P in association with iron minerals, thus limiting the extent of this positive productivity feedback over a timescale of millions of years. By contrast, during deposition of the Schwarzrand Subgroup (547–540 Ma), P was more effectively buried in sediments through sink-switching to authigenic apatite after initial drawdown in association with organic matter and Fe (oxyhydr)oxide minerals (Fig. 4b). The lack of P recycling from oxic sediments of the Schwarzrand Subgoup stabilised oxia in the overlying water column. There is a notable increase in bioturbation intensity recorded in the lowermost Schwarzrand Subgroup^55^, however the potential secondary influence of bioirrigation and mixing-induced sedimentary P retention on local water column productivity remains uncertain.

The consistent spatial separation of anoxic deep waters from the distribution of soft-bodied, skeletal and ichnotaxa in the Nama Group may support the contention that oxygen was a metabolic requirement of these organisms. High resolution sub-sampling of fossiliferous beds in the Nama Group has previously revealed the ability of the skeletal *Namacalathus* to opportunistically colonise the substrate during fleeting oxic episodes under a regime of dominantly ferruginous water column conditions^5^. Similarly, palaeoredox studies of fossiliferous shales of the Blueflower Formation, NW Canada, have been interpreted to show the ability of soft-bodied forms including possible *Pteridinium*, *Inkrylovia* and *Windermeria*, to opportunistically colonise environments that were briefly ventilated for shorter durations than those resolvable by bulk Fe proxy capability^56^. Ferruginous conditions that dominated the deeper water column during deposition of the Kuibis Subgroup may have limited habitable space for soft-bodied macrobiota, the majority of which are preserved in the shallowest environments. However, some *in situ Ernietta* and *Rangea* specimens are also suggested to have been able to opportunistically colonise the substrate during short-lived oxic episodes, similar to the Blueflower Formation, or the ability to survive incursions of anoxic bottom waters^57^.

Whilst there is no paucity of siliciclastic facies in the Witputs sub-basin throughout the Nama succession, the majority of soft-bodied fossil representatives are known only from shallow, shoreface and inner-ramp, quartz-rich sandstone horizons. Particularly prominent fossiliferous quartzites appear in the Aar, Nasep and Spitskop members (see Supplementary Information Table S1 for references)^23,57^. However, a notable trend of increasing bioturbation intensity is present in the shallow inner to mid-ramp during deposition of the Nudaus Formation^55^, within the middle part of the Nama Group succession (Fig. 2g). There is a further increase in bioturbation intensity from the lower Schwarzrand Subgroup to the basal Cambrian unconformity in the Nama Group^26,55^. Indeed, recent quantification of bioturbation intensity in the Nama Group shows an overall increase in the percentage of bedding plane bioturbated from a mean value of 1.94% in the Kliphoek Member to 3.34% in the lower Urusis Formation, and a further increase to 5.61% in the Spitskop Member, immediately beneath the basin-wide unconformity that marks the top of the Urusis Formation (Fig. 2g)^55^. The increase in bioturbation intensity occurs coincident with the stabilisation of oxic conditions in this location (Fig. 2).

The local transition to stable, oxic conditions observed in the Nama succession may have been a prerequisite for the increase in bioturbation intensity in this shallow ramp setting, allowing the efficient exploitation of nutrients held within the sediment and microbial mats. However, the link between oxygenation and increased bioturbation intensity is less clear in broadly contemporaneous successions of South China. Here, sediments of the Dengying Formation contain trace fossils^58^ alongside proxy evidence for only punctuated oxic intervals under a regime of dominantly anoxic water column conditions^9,59^. Evidence from nitrogen isotope studies^60^ of fossiliferous Ediacaran and early Cambrian strata in South China attest to a regional nutrient regime distinct from that of the Nama Group, with high nutrient delivery to the Yangtze platform and slope, likely (in part) supplied by enhanced upwelling, driving an intense and long-lived OMZ throughout this interval^9^. A comparison of the relative rates of ichnotaxa diversification and their spatial distributions between stratigraphic successions of the Nama Group and South China, is required to test the relationship between palaeoredox of equivalent facies and relative diversification rates between these two potentially differing late Ediacaran palaeoenvironments.

Skeletal macrofossil assemblages of the Ediacaran attain their highest abundance and diversity in the Kuibis Subgroup of the Nama Group, where surface and mid-depths were occasionally well oxygenated but deeper waters were anoxic and nutrient rich^5,6^. Indeed, iron speciation data support the importance of a dominantly oxic local environment for the most diverse skeletal assemblage in the Nama basin (Driedoornvlakte, Figure S7). In the Dengying Formation, the shallowest environments of Shaanxi province and the Baimatuo Member in the Yangtze Gorges host relatively depauperate, microbial mat-related skeletal assemblages (e.g.^61^), where rare earth element profiles suggest continued redox instability with only short-lived oxic intervals^59^. The differing geochemical characteristics observed between palaeoenvironments of the Nama Group and Yangtze Platform may suggest a delicate balance between oxygen demand and nutrient loading as an incentive for the radiation of motile macrobiota, and potentially for ecosystem habitation by skeletal organisms in the late Ediacaran. This may allude to a ‘sweet spot’ in oxygen and nutrient demand for skeletonising and filter feeding ecologies in the late Ediacaran, however this remains to be tested.

While geochemical evidence from the uranium isotopic composition of carbonates (δ^238^U_carb_) points to an increase in the global extent of anoxic ocean waters at ~547 Ma^62,63^, the redox data reported here show that, on a local scale, the Nama basins became more stable and oxic at this time. These global and local datasets are not necessarily in conflict, as the uranium proxy data may record an integrated global increase in the spatial extent of productive mid-depth waters at ~547 Ma. In fact, recent compilations of δ^238^U_carb_ from globally distributed late Ediacaran successions do suggest a trend towards an increasingly oxygenated global ocean between 545–540 Ma, in the wake of the highly negative values recorded in the lower Nama Group and Dengying Formation^62–64^. However, these global oxygenation events were transient, and their influence on the pace of animal evolution in shallow shelf ecosystems which were subject to highly heterogeneous local redox conditions demands further investigation.

## Conclusions

Soft-bodied macrobiota occupied a predominantly intertidal to shallow sub-tidal setting throughout deposition of the Nama Group, likely at least partly due to the constraint of proximal deep water anoxia^5,6^. Stable and long-lived oxia in shallow marine oases supported the most diverse skeletal communities in mid-ramp settings^5,6^. Both sub-basins exhibit a shift towards widespread oxic stabilisation between ~547 and ~542 Ma that is accompanied by a distinct transition from high average values of CIA in the Kuibis Subgroup to low values in the Schwarzrand Subgroup. The combined trends in redox and weathering proxies may reflect a reduced supply of continentally derived nutrients, potentially linked to a change in regional climate. This, in turn, reduced primary productivity from initial conditions that were conducive to basin wide anoxia during organic matter remineralisation, to normal oxic marine production. Fe and P speciation studies within the context of evolving basin sedimentology provide evidence for distinct P recycling mechanisms between initially redox stratified conditions in the Kuibis Subgroup and stable, oxic conditions of the overlying Schwarzrand Subgroup. During deposition of the Schwarzrand Subgroup, efficient sink-switching of organic and Fe oxide bound P to authigenic phases was promoted under oxic water column conditions, which may have further stabilised oxic conditions through limiting pore water P recycling. Oxic stabilisation of the Nama sub-basins was accompanied by progressive occupation by active, motile trace makers, which thrived first in shallow inner ramp settings, but later in mid-ramp, clastic environments.

## Supplementary information

Supplementary information.

## References

[1] Pu JP (2016). Dodging snowballs: Geochronology of the Gaskiers glaciation and the first appearance of the Ediacaran biota. Geology.

[2] Seilacher A, Buatois LA, Mángano MG (2005). Trace fossils in the Ediacaran-Cambrian transition: Behavioral diversification, ecological turnover and environmental shift. Palaeogeogr. Palaeoclimatol. Palaeoecol..

[3] Germs GJB (1972). New shelly fossils from Nama Group, South West Africa. American Journal of Science.

[4] Sperling, E. A., Knoll, A. H. & Girguis, P. R. The Ecological Physiology of Earth’s Second Oxygen Revolution. *Annu. Rev. Ecol. Evol. Syst*. **46**, annurev-ecolsys-110512-135808 (2015).

[5] Wood RA (2015). Dynamic redox conditions control late Ediacaran metazoan ecosystems in the Nama Group, Namibia. Precambrian Res..

[6] Tostevin R (2016). Low-oxygen waters limited habitable space for early animals. Nat. Commun..

[7] Sperling EA (2015). Statistical analysis of iron geochemical data suggests limited late Proterozoic oxygenation. Nature.

[8] Och LM (2016). Palaeoceanographic controls on spatial redox distribution over the Yangtze Platform during the Ediacaran-Cambrian transition. Sedimentology.

[9] Bowyer F, Wood RA, Poulton SW (2017). Controls on the evolution of Ediacaran metazoan ecosystems: A redox perspective. Geobiology.

[10] Guilbaud, R. *et al*. Oxygen minimum zones in the early Cambrian ocean. *Geochemical Perspect. Lett*. 33–38. 10.7185/geochemlet.1806 (2018)

[11] Tyrrell T (1999). The relative influences of nitrogen and phosphorus on oceanic primary production. Nature.

[12] Germs GJB (1983). Implications of a sedimentary facies and depositional environmental analysis of the Nama Group in South West Africa/Namibia. Spec. Publ. Geol. Soc. South Africa.

[13] Grotzinger JP, Bowring SA, Saylor BZ, Kaufman AJ (1995). Biostratigraphic and Geochronologic Constraints on Early Animal Evolution. Science (80-.)..

[14] Bowring SA (2007). Geochronologic constraints on the chronostratigraphic framework of the Neoproterozoic Huqf Supergroup, Sultanate of Oman. Am. J. Sci..

[15] Linnemann Ulf, Ovtcharova Maria, Schaltegger Urs, Gärtner Andreas, Hautmann Michael, Geyer Gerd, Vickers-Rich Patricia, Rich Tom, Plessen Birgit, Hofmann Mandy, Zieger Johannes, Krause Rita, Kriesfeld Les, Smith Jeff (2018). New high-resolution age data from the Ediacaran-Cambrian boundary indicate rapid, ecologically driven onset of the Cambrian explosion. Terra Nova.

[16] Saylor BZ, Kaufman AJ, Grotzinger JP, Urban F (1998). A composite reference section for terminal proterozoic strata of southern Namibia. J. Sediment. Res..

[17] Kaufman AJ, Hayes JM, Knoll AH, Germs GJB (1991). Isotopic compositions of carbonates and organic carbon from upper Proterozoic successions in Namibia: stratigraphic variation and the effects of diagenesis and metamorphism. Precambrian Res..

[18] Saylor B, Grotzinger JP, Germs G (1995). Sequence stratigraphy and sedimentology of the Neoproterozoic Kuibis and Schwarzrand Subgroups (Nama Group), southwestern Namibia. Precambrian Res..

[19] Saylor BZ (2003). Sequence stratigraphy and carbonate-siliciclastic mixing in a terminal Proterozoic foreland basin, Urusis Formation, Nama Group, Namibia. J. Sediment. Res..

[20] Kaufman AJ, Jacobsen SB, Knoll AH (1993). The Vendian record of Sr and C isotopic variations in seawater: Implications for tectonics and paleoclimate. Earth Planet. Sci. Lett..

[21] Gurich G (1930). Uber den Kuibisquartzit in Sudwest-afrika. Zeitschrift Dtsch. Geol. Gesellschaft.

[22] Germs GJB (1973). A reinterpretation of Rangea schneiderhoehni and the discovery of a related new fossil from the Nama Group, South West Africa. Lethaia.

[23] Narbonne GM, Saylor BZ, Grotzinger JP (1997). The youngest Ediacaran fossils from southern Africa. J. Paleontol..

[24] Germs GJB, Knoll AH, Vidal G (1986). Latest proterozoic microfossils from the Nama group, Namibia (south west Africa). Precambrian Res..

[25] Wood RA, Grotzinger JP, Dickson JAD (2002). Proterozoic Modular Biomineralized Metazoan from the Nama Group, Namibia. Science (80-.)..

[26] Jensen SM, Runnegar BN (2005). A complex trace fossil from the Spitskop Member (terminal Ediacaran–? Lower Cambrian) of southern Namibia. Geol. Mag..

[27] Buatois LA, Almond J, Mángano MG, Jensen S, Germs GJB (2018). Sediment disturbance by Ediacaran bulldozers and the roots of the Cambrian explosion. Sci. Rep..

[28] Poulton S, Canfield D (2005). Development of a sequential extraction procedure for iron: implications for iron partitioning in continentally derived particulates. Chem. Geol..

[29] Poulton SW, Krom MD, Raiswell R (2004). A revised scheme for the reactivity of iron (oxyhydr)oxide minerals towards dissolved sulfide. Geochim. Cosmochim. Acta.

[30] Poulton SW, Fralick PW, Canfield DE (2004). The transition to a sulphidic ocean, 1.84 billion years ago. Nature.

[31] Poulton SW, Raiswell R (2002). The low-temperature geochemical cycle of iron: From continental fluxes to marine sediment deposition. Am. J. Sci..

[32] Poulton SW, Canfield DE (2011). Ferruginous Conditions: A Dominant Feature of the Ocean through Earth’s History. Elements.

[33] Raiswell R (2008). Turbidite depositional influences on the diagenesis of Beecher’s Trilobite Bed and the Hunsrück Slate; sites of soft tissue pyritization. Am. J. Sci..

[34] Clarkson MO, Poulton SW, Guilbaud R, Wood R (2014). a. Assessing the utility of Fe/Al and Fe-speciation to record water column redox conditions in carbonate-rich sediments. Chem. Geol..

[35] Saylor BZ, Grotzinger JP (1996). Reconstruction of important Proterozoic-Cambrian boundary exposures through the recognition of thrust deformation in the Nama Group of southern Namibia. Commun. - Geol. Surv. Namibia.

[36] Wood R, Bowyer F, Penny A, Poulton SW (2018). Did anoxia terminate Ediacaran benthic communities? Evidence from early diagenesis. Precambrian Res..

[37] Nesbitt HW, Young GM (1982). Early Proterozoic climates and plate motions inferred from major element chemistry of lutites. Nature.

[38] Thompson J (2019). Development of a modified SEDEX phosphorus speciation method for ancient rocks and modern iron-rich sediments. Chem. Geol..

[39] Ruttenberg KC (1992). Development of a sequential extraction method for different forms of phosphorus in marine sediments. Limnol. Oceanogr..

[40] Turekian KK, Wedepohl KH (1961). Distribution of the Elements in Some Major Units of the Earth’s Crust. Geol. Soc. Am. Bull..

[41] Ingall E, Jahnke R (1994). Evidence for enhanced phosphorus regeneration from marine sediments overlain by oxygen depleted waters. Geochim. Cosmochim. Acta.

[42] Slomp CP, Thomson J, De Lange GJ (2002). Enhanced regeneration of phosphorus during formation of the most recent eastern Mediterranean sapropel (S1). Geochim. Cosmochim. Acta.

[43] Ruttenberg K, Berner R (1993). Authigenic apatite formation and burial in sediments from non-upwelling, continental margin environments. Geochim. Cosmochim. Acta.

[44] Reinhard CT (2017). Evolution of the global phosphorus cycle. Nature.

[45] Ingall ED, Bustin RM, Van Cappellen P (1993). Influence of water column anoxia on the burial and preservation of carbon and phosphorus in marine shales. Geochim. Cosmochim. Acta.

[46] Slomp CP, Epping EHG, Helder W, Van Raaphorst W (1996). A key role for iron-bound phosphorus in authigenic apatite formation in North Atlantic continental platform sediments. J. Mar. Res..

[47] Xiong Y (2019). Phosphorus cycling in Lake Cadagno, Switzerland: A low sulfate euxinic ocean analogue. Geochim. Cosmochim. Acta.

[48] Slomp, C. P., Van Der Gaast, S. J. & Van Raaphorst, W. Phosphorus binding by poorly crystalline iron oxides in North Sea sediments. **52**, 55–73 (1996).

[49] White AE, Spitz YH, Karl DM, Letelier RM (2006). Flexible elemental stoichiometry in Trichodesmium spp. and its ecological implications. Limnol. Oceanogr..

[50] März C, Poulton SW, Wagner T, Schnetger B, Brumsack HJ (2014). Phosphorus burial and diagenesis in the central Bering Sea (Bowers Ridge, IODP Site U1341): Perspectives on the marine P cycle. Chem. Geol..

[51] Slomp Caroline P., Mort Haydon P., Jilbert Tom, Reed Daniel C., Gustafsson Bo G., Wolthers Mariette (2013). Coupled Dynamics of Iron and Phosphorus in Sediments of an Oligotrophic Coastal Basin and the Impact of Anaerobic Oxidation of Methane. PLoS ONE.

[52] Schmitz, M. D. Radiogenic Isotope Geochronology. in *The Geological Time Scale 2012* (eds Gradstein, F. M., Ogg, J. G., Schmitz, M. D. & Ogg, G. M.) (Elsevier, 2012).

[53] Germs GJB, Gaucher C (2012). Nature and Extent of a Late Ediacaran (Ca. 547 Ma) Glacigenic Erosion Surface in Southern Africa. South African J. Geol..

[54] Gaucher, C., Frimmel, H. E. & Germs, G. J. B. Tectonic Events and Palaeogeographic Evolution of Southwestern Gondwana in the Neoproterozoic and Cambrian. in *Neoproterozoic to Cambrian Tectonics, Global Change and Evolution: a Focus on Southwestern Gondwana. Developments in Precambrian Geology* (eds Gaucher, C., Sial, A. N., Halverson, G. P. & Frimmel, H. E.) **16**, 295–316 (Elsevier, 2009).

[55] Cribb AT (2019). Increase in metazoan ecosystem engineering prior to the Ediacaran–Cambrian boundary in the Nama Group, Namibia. R. Soc. Open Sci..

[56] Sperling EA (2015). Oxygen, facies, and secular controls on the appearance of Cryogenian and Ediacaran body and trace fossils in the Mackenzie Mountains of northwestern Canada. Bull. Geol. Soc. Am..

[57] Hall M (2013). Stratigraphy, palaeontology and geochemistry of the late Neoproterozoic Aar Member, southwest Namibia: Reflecting environmental controls on Ediacara fossil preservation during the terminal Proterozoic in African Gondwana. Precambrian Res..

[58] Meyer M (2014). Interactions between Ediacaran animals and microbial mats: Insights from Lamonte trevallis, a new trace fossil from the Dengying Formation of South China. Palaeogeogr. Palaeoclimatol. Palaeoecol..

[59] Ling HF (2013). Cerium anomaly variations in Ediacaran-earliest Cambrian carbonates from the Yangtze Gorges area, South China: Implications for oxygenation of coeval shallow seawater. Precambrian Res..

[60] Cremonese L, Shields-Zhou GA, Struck U, Ling HF, Och LM (2014). Nitrogen and organic carbon isotope stratigraphy of the Yangtze Platform during the Ediacaran-Cambrian transition in South China. Palaeogeogr. Palaeoclimatol. Palaeoecol..

[61] Cai Y, Hua H, Schiffbauer JD, Sun B, Yuan X (2014). Tube growth patterns and microbial mat-related lifestyles in the Ediacaran fossil Cloudina, Gaojiashan Lagerstätte, South China. Gondwana Res..

[62] Tostevin R (2019). Uranium isotope evidence for an expansion of anoxia in terminal Ediacaran oceans. Earth Planet. Sci. Lett..

[63] Zhang F (2018). Extensive marine anoxia during the terminal Ediacaran Period. Sci. Adv..

[64] Zhang F (2019). Global marine redox changes drove the rise and fall of the Ediacara biota. Geobiology.

