# Supplementary material for: Regional nutrient decrease drove redox stabilisation and metazoan diversification in the late Ediacaran Nama Group, Namibia
Source: Sci Rep. 2020 Feb 10;10:2240. doi: 10.1038/s41598-020-59335-2 (PMC7010733; doi:10.1038/s41598-020-59335-2)
Supplement: Supplementary file 1 — Supplementary information. [file 41598_2020_59335_MOESM1_ESM.docx]

**Supplementary Material for:**

**“Regional nutrient decrease drove redox stabilisation and metazoan diversification in the late Ediacaran Nama Group, Namibia”**

Bowyer, F.T.^1+^*, Shore, A.J.^1^, Wood, R.A.^1^, Alcott, L.J.^2^, Thomas, A.L.^1^, Butler, I.B.^1^, Curtis, A.^1^, Hainanan, S.^3^, Curtis-Walcott, S.^4^, Penny, A.M.^5^, and Poulton, S.W.^2^

^1^University of Edinburgh, School of GeoSciences, James Hutton Road, Edinburgh, EH9 3FE, UK. ^2^ University of Leeds, School of Earth and Environment, Leeds, LS2 9JT, UK.  ^3^ Ministry of Mines and Energy, 6 Aviation Road, Private Bag 13297, Windhoek, Namibia
^4^ Department of Physics, Imperial College, London SW7 2AZ
^5^ Finnish Museum of Natural History, University of Helsinki, Jyrängöntie 2, 00560 Helsinki, Finland

+ current address: University of Leeds, School of Earth and Environment, Leeds, LS2 9JT, UK.

*email: [F.T.Bowyer@leeds.ac.uk](mailto:F.T.Bowyer@leeds.ac.uk).

1. **Analytical Protocols**

**Sample preparation**

Outcrop shale samples were taken with a minimum mass of ~40 g, weathered surfaces were removed and samples were divided in two with a diamond saw, washed and dried at 40°C. Samples with visible signs of alteration and/or veining were rejected at this point. Halved samples were crushed and pulverised to homogeneous powder (<60 µm) using a tungsten carbide jaw crusher and disc mill.

**Total digestion**

After ashing at 550°C for 8 hours, samples were quantitatively dissolved in trace metal grade HNO_3_, HF, and HClO_4_, heated in open PTFE cups and left to dry fully over a period of 24 hours before addition of H_3_BO_3_ to prevent the formation of Al complexes. Dry residues were then dissolved in concentrated HNO­_3_ and diluted with ultrapure 18MΩ H_2_O. Resultant solutions were analysed on a Varian Vista-Pro CCD simultaneous ICP-OES at the University of Edinburgh, Grant Institute with replicate analyses yielding a RSD of <3% (Table S3).

**Fe speciation**

Iron speciation analyses follow the method of (1). An initial leach targeting iron bound in carbonate phases (Fe_carb_) employed Na-acetate, buffered to pH 4.5 with acetic acid and agitated at 50°C for 48 hrs. This was followed by a 2hr iron oxide (Fe_ox_) extraction in Na-dithionite buffered to pH 4.8 and a final extraction of magnetite (Fe_mag_) with ammonium oxalate for 6 hrs. All steps of the sequential leach were performed at the Cohen Laboratories, Leeds University, School of Earth and Environment, and resultant solutions were analysed for Fe using a Thermo Scientific iCE-3000 series flame atomic absorption spectrometer with replicate extractions for each step yielding RSDs of <5% (Table S4).

The concentration of pyrite iron (Fe_py_) was determined through a boiling chromous chloride distillation with a pre-leach in boiling 6 M HCl for quantitative extraction of acid volatile sulfide (AVS) after the method of (2). Weight percent AVS and Fe_py_ were determined gravimetrically after stoichiometric precipitation of Ag_2_S.

**P speciation**

A modified version of the SEDEX method (3), attuned for the analysis of ancient sedimentary rocks (4) was used in the quantitative assessment of P phases. One major advantage of the modified sequential extraction procedure is the ability to target P bound in haematite and magnetite (4) in addition to authigenic, organic and detrital P.

**Method**

Approximately 0.15 – 0.19 g sample powder was subjected to the modified sequential extraction scheme (4). A graphical representation of the procedural protocol is shown in Figure S1. Following each step in the sequential leach, samples were centrifuged at 4000 rpm for 4 minutes and aliquots of 8 ml were taken from the supernatant solution for analysis. The remaining supernatant in the reaction tube was then decanted to waste and the unreacted sample was subjected to the following extraction step. Some steps in the procedure required additional washes to ensure quantitative extraction of targeted P. The P_Fe1_, P_mag_ and P_Fe2_ extraction steps were each followed by one wash in MgCl_2_ and the P_auth_ extraction was followed by one MgCl_2_ wash and one wash in ultrapure 18MΩ H_2_O if the absorbance of undiluted samples was greater than 0.1. The sodium citrate/-dithionite/acetic acid (CDA) reagent, used for the extraction of P_Fe2_, and sodium citrate/-bicarbonate/-dithionite (CBD) reagent, used for the extraction of P_Fe1_, were prepared on the day of use.

For the extraction of P_org_, samples were transferred to porcelain crucibles using 4-6 ml of MilliQ H_2_O, oven dried at 100°C and then ashed for 2 hours at 550°C in a Carbolite furnace. After cooling, samples were quantitatively transferred to the reaction tubes using 10% HCl. The samples were then agitated for 16 hours at 100 rpm.

Multiple stages in the procedure required P measurements via spectrophotometry using molybdate blue, whereby formation of phosphomolybdate in the presence of P was measured at a wavelength of 880 nm. Supernatant solutions from the P_Fe1_, P_mag_ and P_Fe2_ extractions were measured by ICP-OES in the Cohen Laboratories, School of Earth and Environment, University of Leeds. Solutions were spiked with an internal standard of 100 μl Co (100 ppm). Quality check solutions of 0 and 2 ppm P were run every 10 samples.


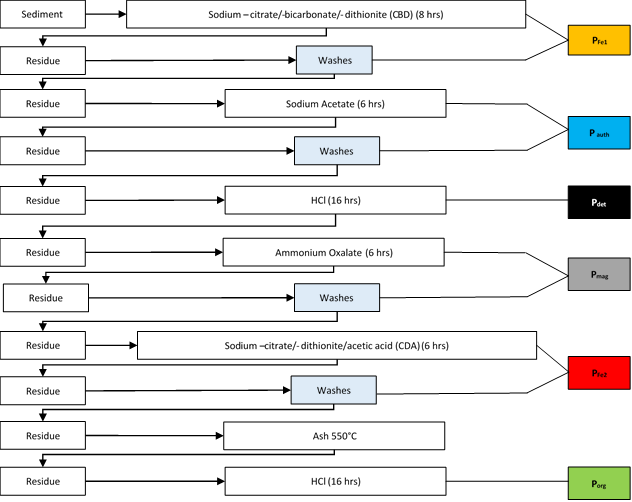


**Figure S1.** The modified P speciation protocol for ancient sediments utilised in this study.

**Critical assessment of P extraction**

Of the 47 samples selected for P speciation, 39 show recovery of >70% P_Tot_ (mean = 90.8%) following the sequential P extraction (Figure S2a). The sample with the lowest P recovery (OMK3/8, data not used in this study) also had the highest Al concentration (19 wt%). The negative correlation between P recovery and Al is a recognised complication in P studies of modern sediments^5^. However, there is no strong correlation between P_det_ (as a percentage of P_Tot_) and Al (Figure S2b) for samples with P_SUM_/P_Tot_ >0.7, suggesting that elevated Al was not a compounding issue for P recovery in these samples.

A specific complication in the analysis of ancient sedimentary rocks via the modified SEDEX method involves the potential transfer of P_auth_ to the P_det_ pool as a consequence of the post-depositional recrystallisation of authigenic apatite during burial diagenesis^5^. It has been noted that the detrital P content of modern continental margin sediments is 186 ± 21 ppm^6^, whereas modern oligotrophic settings are characterised by P_det_ in the range 62 – 310 ppm^7^. Samples from the Nama Group have P_det_ concentrations in the range 4.2 – 536.4 ppm (mean 208.7 ppm), with maximum values significantly greater than average P_det_ of modern shelf environments. This may suggest that a portion of extracted P_det_ represents burial recrystallisation of initially authigenic P. There is no significant correlation between P_reac_ (as a percentage of P_Tot_) and Al (r^2^ = 0.077, Figure S2c), implying negligible contamination of the P_auth_ pool by P_det_. Consequently, concentrations of P_auth_ (and by extension summed P_reac_) likely represent minimum values, whilst those of P_det_ represent maximum values, which reinforces our interpretation of elevated authigenic P contribution under oxic conditions in the Nama Group.


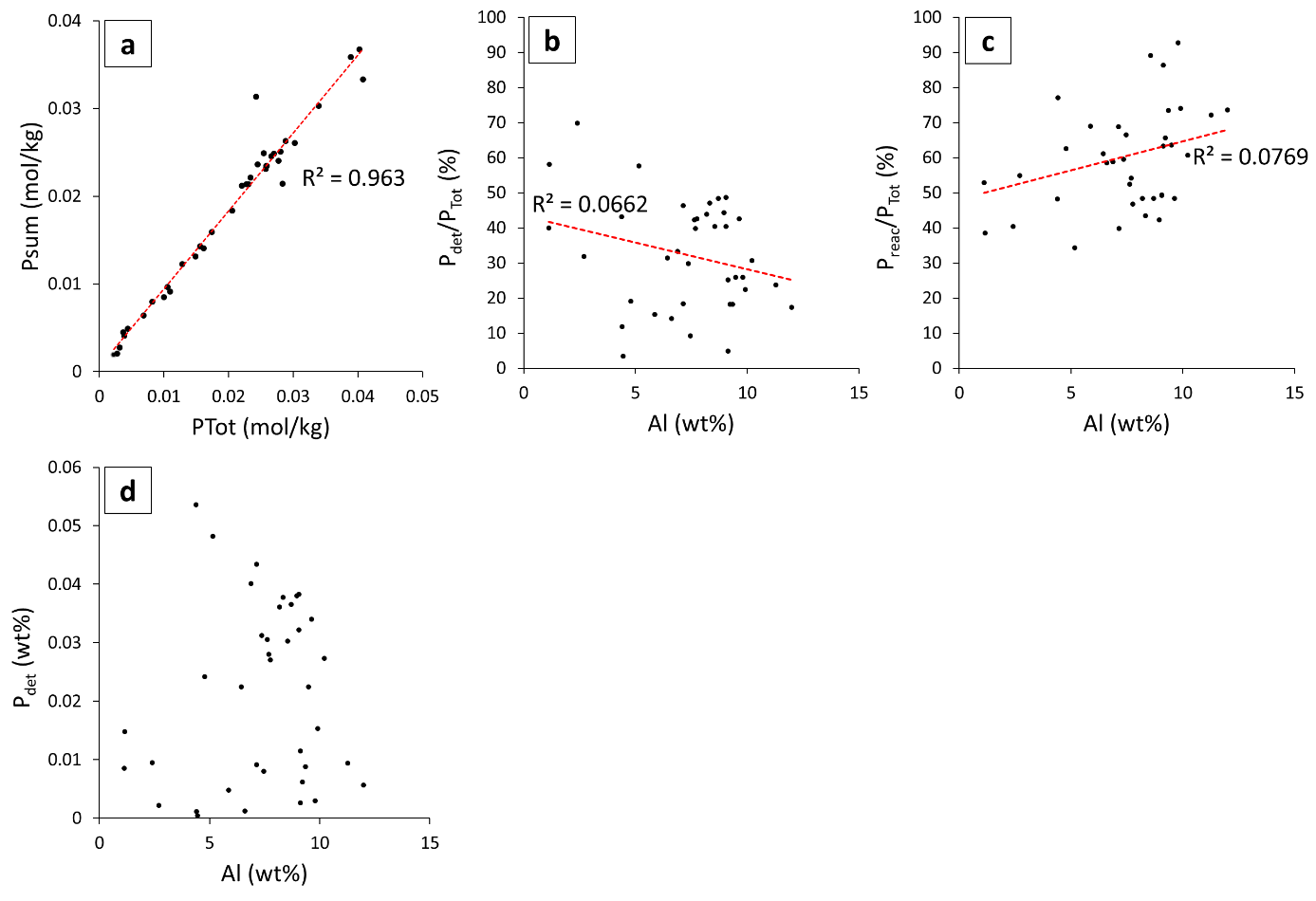


**Figure S2.** The relationships between extracted P phases and the detrital background (represented by the concentration of Al).

1. **Kuibis Subgroup inter-section Carbon isotope correlation**

With the purpose of analysing major shifts in the redox state of the Nama basin as a whole, recognising redox independence between the two sub-basins and directly relating the stratigraphic position of fossil occurrences, it was first necessary to confidently correlate relative sample position across the sub-basin divide. During deposition of the Schwarzrand Subgroup, major depositional sequences have been defined within both the Zaris and Witputs sub-basins that show increasing sub-basin connectivity nearing the Ediacaran-Cambrian boundary unconformity^8,9^. By contrast, deposits of the underlying Kuibis subgroup show distinct patterns of preserved systems tracts between the two sub-basins ^10,11^. Previous efforts at integrated regional section correlation, employing carbon isotope chemostratigraphy and detailed sequence stratigraphic mapping have proved highly successful in the Nama and Witvlei Groups^11,12^. Based on this framework, published δ^13^C_carb_ data^13,14^ for each studied section are used in construction of a composite δ^13^C_carb_ curve tied to major sequence boundaries in order to aid section correlation of the Kuibis subgroup across the Osis Arch (Figure S4). Additional data of the Huns Member^12^ are included for completion (Figure 2).

Throughout this discussion, reference will be made to sampled sections noted in Figure S3. The composite carbon isotope curve defined by (11) is characterised by four intervals of discrete and correlatable trends, summarised below. The first (Npg) interval is defined by negative δ^13^C_carb_ values as low as -6‰ to -7‰ that gradually recover to 0‰ in the lower Kuibis Subgroup^11^ (Figure S4). The Npg interval was recognised from postglacial stratigraphy of the Buschmannsklippe Formation in the Gobabis and Witvlei area, east of Windhoek (inset Figure S3). The rising limb of the Npg interval characterises overlying deposits of the Dabis Formation (Kuibis Subgroup) in both the Zaris and Witputs sub-basins. However, it is now thought likely that the unconformity separating Buschmannsklippe from Nama stratigraphy in the Gobabis-Witvlei area represents a major depositional hiatus (K.-H. Hoffmann pers. com.). As such, the negative δ^13^C excursion recorded in the Buschmansklippe Formation may instead correlate with strata immediately post-dating Marinoan-age glacial diamictites (e.g. Maieberg Formation of the Congo Craton, northwest Namibia^15^).

In the Witputs sub-basin, the Npg interval continues through sequence K1 and lower sequence K2 (Mara to Lower Kliphoek members) of (10). In the Zaris sub-basin, the Npg interval is recorded from the Kanies Member to the maximum flooding surface of the Lower Omkyk Member (OS1), with the notable exception of Farm Omkyk, where the transition to positive δ^13^C_carb_ occurs up to 30 m below the MFS of OS1^14^. The second (Pr) interval is defined by a rising limb from ~0‰ to maximum values in the range 4 to 5‰^11^. The apex of this trend is recorded in the Omkyk Member of the Zaris Formation (Kuibis Subgroup) and is followed by a gradual decline in δ^13^C_carb_ (Pf interval), culminating in a brief


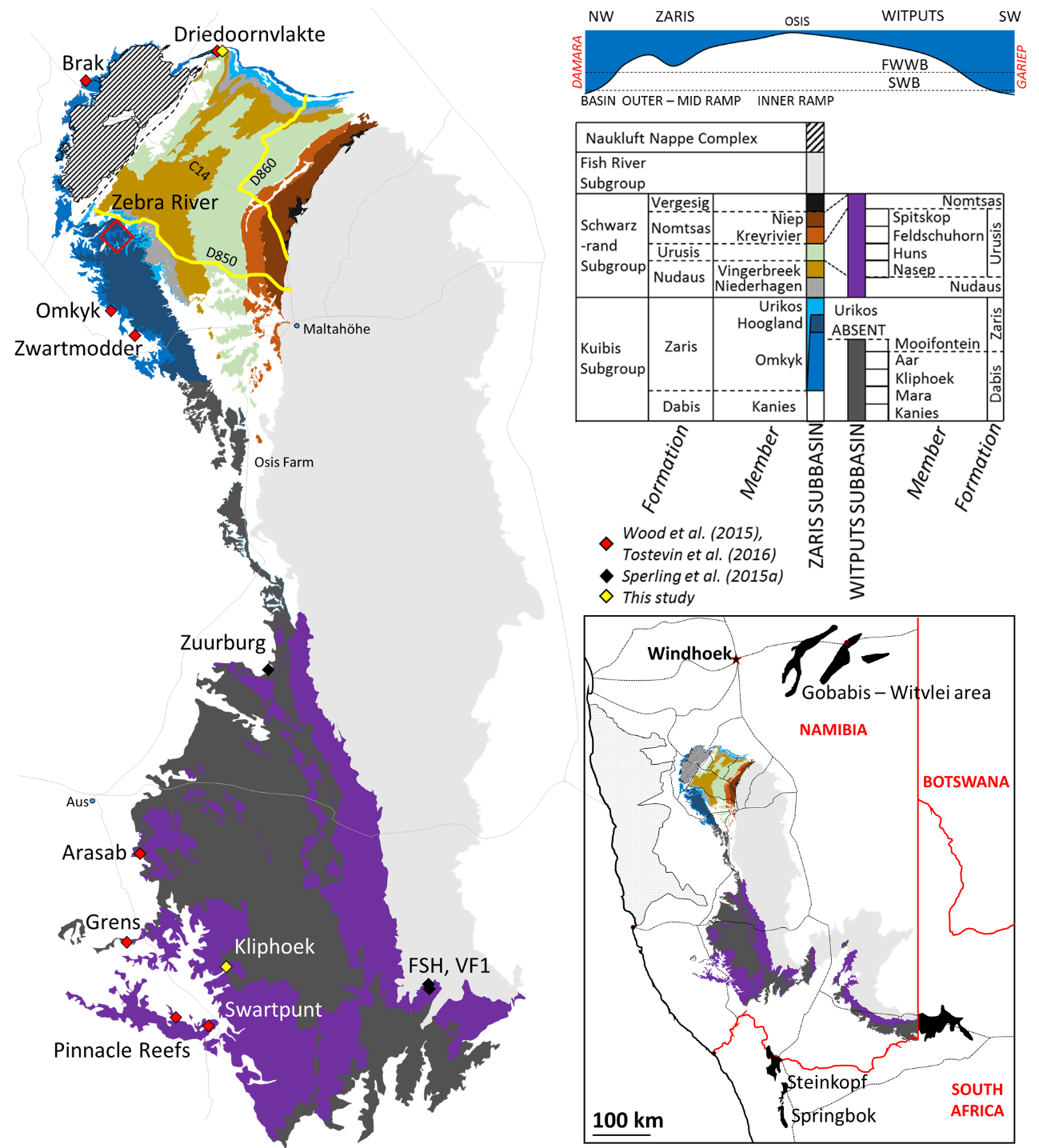


**Figure S3.** Map of Nama Group outcrop, showing section locations.

return to mildly negative values in the Upper Zaris Formation. The Pf interval, which largely corresponds to sequence K3 of (10), is notably absent from stratigraphy of the Witputs sub-basin due to non-deposition or erosion. The latter is evident in some areas where K2 is capped by an erosive unconformity, infilled by clastic sediment of the Nudaus Formation^11^. Carbonates of the overlying Schwarzrand Subgroup (Huns and Spitskop members of the Urusis Formation) record positive and relatively invariant δ^13^C_carb_ values (Figure 2)^12,14^ that conform to the globally recognised late Ediacaran positive carbon isotope plateau (EPIP)^16^.

Recently, regional carbon isotopic data published in (14), and additional data of (13) from the Driedoornvlakte carbonate platform have greatly increased the chemostratigraphic resolution of the Nama Group. Furthermore, Fe-speciation and δ^13^C_carb_ data reported by (14) were collected in tandem from sampled sections, thus allowing relative sample position to be approximated between sections across the sub-basin divide (Figure S4).

The most negative δ^13^C_carb_ values reported from Brak and Grens sections are lower than values reported from the Npg interval of (11) (Figure S4). The Kanies Member represents diachronous deposition upon a regional transgressive surface that unconformably separates Nama sediments from the underlying crystalline Proterozoic basement^8^. Chemostratigraphic correlation of two-dimensional stratigraphic sections requires consideration of the inherent variation in δ^13^C_carb_ with depth in the water column at which carbonate mineral precipitation occurred. Autotrophic C fixation in the photic zone preferentially incorporates the light isotope (^12^C) during organic matter formation, resulting in elevated ^13^C/^12^C of shallow waters. Organic matter remineralisation during downward transport in the water column liberates ^12^C and may therefore result in decreasing ^13^C/^12^C with depth until all organic matter has been oxidised (e.g. 17). Therefore, the most negative δ^13^C_carb_ recorded in deposits of the Dabis Formation may be expected in sections where the deepest facies are preserved. Carbon isotope values are also consistently depleted in some samples from lagoonal relative to open marine facies in the Nama Group^12^, and attributed to alteration during organic matter reoxidation. Whilst carbonate sediments of the deepest and oldest section in the Zaris sub-basin (outer ramp at Brak), do record highly negative δ^13^C_carb_, shallow inner and mid-ramp open marine carbonates immediately overlying the transgressive surface in the Witputs sub-basin at Arasab and Grens also record correspondingly negative values^14^.

At Brak, δ^13^C­_carb_ values fall in the lower Kanies Member from -1.68‰ to a nadir of-7.4‰. As such,


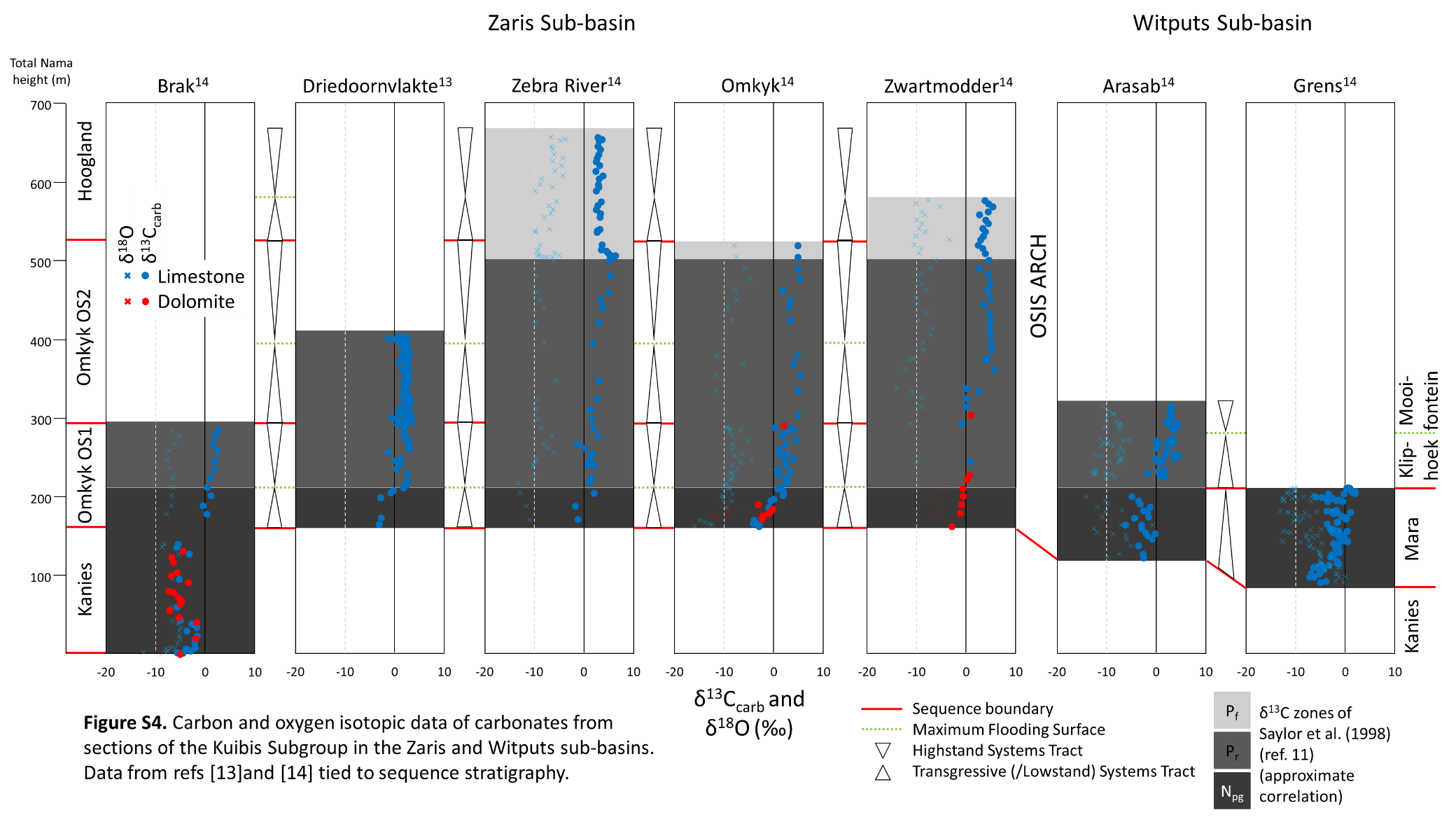


the lower Kanies Member at Brak is considered to represent the oldest transgressive unit of the classic Nama Group sampled to date and here the δ^13^C_carb_ nadir preserves the local expression of the Shuram – Wonoka excursion on the Kalahari Craton, as previously proposed^11,14^.

1. **Geological sampling sites**

Here, sampling transects were chosen to infill major stratigraphic gaps in published geochemical records of the Nama Group. These include down-dip deposits at the prominent carbonate platform locality of Driedoornvlakte, the Nudaus Formation of the Witputs sub-basin exposed on Farm Kliphoek and deposits of the Schwarzrand subgroup of the Zaris sub-basin. Published data are also incorporated, including Fe-speciation of carbonates and shales presented in (14) and Fe-speciation of shales presented in (18). When including these data, particular attention has been given to interpretations of relative palaeodepth between sections that, in some instances, warranted further outcrop study.

1. **Farm Zuurburg**

As previously noted, the Dabis Formation was deposited during regional transgression atop Proterozoic basement of the Kalahari Craton, with the oldest deposits and most complete sections preserved to the northwest and southwest in the Zaris and Witputs sub-basins, respectively^8^. Consequently, relative section thickness decreases with proximity to the Osis Arch and the Kalahari Craton. This is exemplified by the relative thicknesses of the Mara Member between sections at farms Grens (~110 m^14^), Arasab (~30 m^14^) and Zuurburg (11.2 m^18^) (Figure S3). Whilst strata at Farm Zuurburg have previously been interpreted to represent an ‘outer shelf’ environment^18^, we interpret the interbedded shales and micrites at this section in context of the regional Nama stratigraphy to represent deposition in a broad, shallow inner ramp environment that was subject to relatively low rates of deposition. This interpretation is consistent with a detailed sedimentological study of the Kanies and Mara members (reassigned to Kliphoek Member by [19]) on neighbouring Farms Wegkruip and Hansburg, which concluded a low-energy, storm-influenced marine/lagoonal environment of deposition for the fine-grained sediments in this area^20^. By contrast, Mara Member deposits at Farms Arasab and Grens are interpreted to represent deposition on the more distal inner ramp, where accommodation space was greater^14^. Transgressive onlap during deposition of the Mara Member saw evaporitic conditions at both Arasab and Grens replaced by deeper siliciclastics and carbonates that were deposited coeval with shallow Mara Member siliciclastics on the more proximal inner ramp at Zuurburg.

1. **Farm Omkyk**

The sampled section on Farm Omkyk has been interpreted to represent deposition in a deep to shallow inner ramp setting^14^. The lower ~73 m of section is composed of thinly bedded lime mudstone interbedded with turbiditic calcisiltite, grainstone and minor shale of the Lower Omkyk Member, deposited during a basin-wide marine transgression. The samples of the Lower Omkyk are herein assigned to the mid-ramp, consistent with sedimentary features that argue for a relatively deep environment of deposition below fair weather wave base but above storm wave base. The Upper Omkyk Member is characterised by a basal unit of interbedded green shale and sandstone overlain by dark limestone grainstone and minor thrombolites with thin dolomite interbeds. The shallow environment recorded by the Upper Omkyk Member is typified by shoaling cycles that demonstrate occasional inter-tidal deposition^14^ and is herein assigned to the distal inner ramp. At the base of the Upper Omkyk Member (24° 47.461'S, 16° 13.265'E), an ~20 cm thick resistant quartzite unit, interbedded with grainstone, contains specimens of *Palaeopasichnus* (Figure S5, personal observation), similar in morphology to features proposed to represent trace fossils with spreiten described from approximately the same stratigraphic level, further to the northwest on Farm Hauchabfontein^21^. Overlying thick bedded grainstones contain minor thrombolites, and abundant *Namacalathus* and *Cloudina* *hartmannae*^14^.


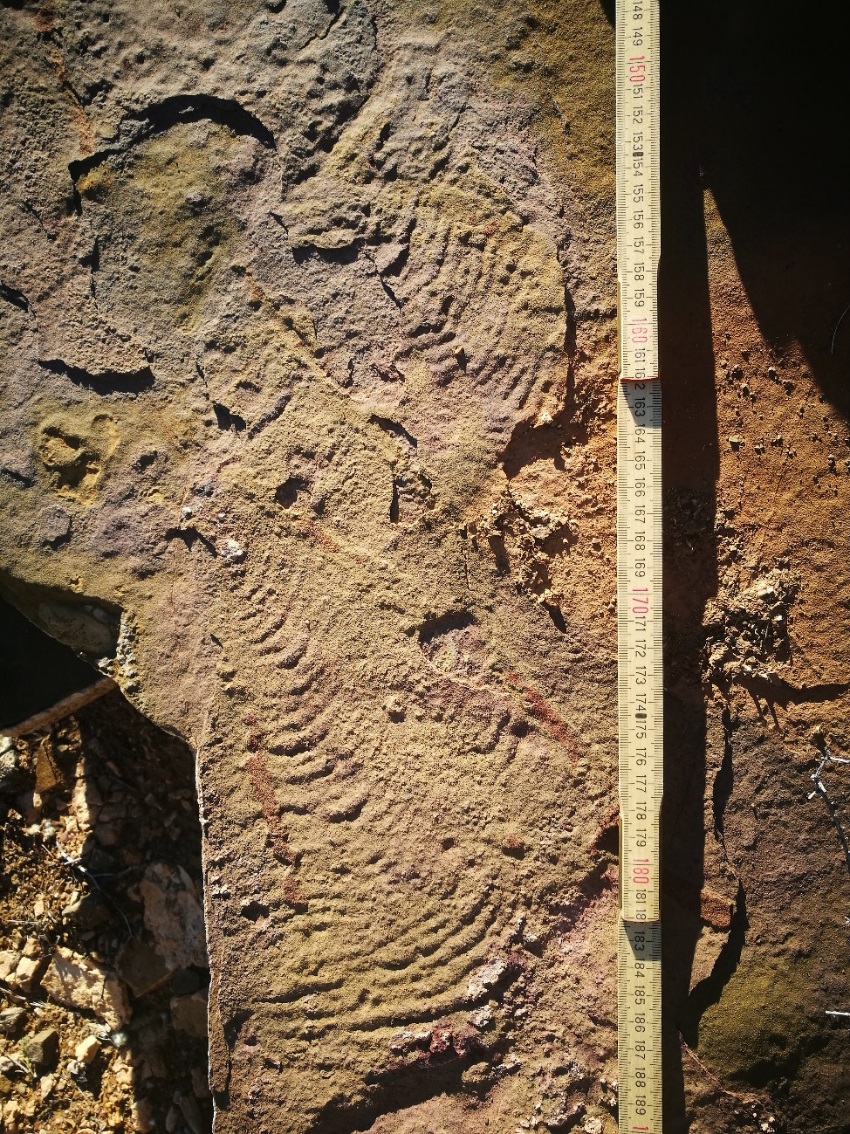


**Figure S5**. *Palaeopasichnus* exposed in the upper Omkyk Member on Farm Omkyk.

1. **Farm Driedoornvlakte**

Isolated carbonate platform deposits with capping microbial-metazoan pinnacle reefs grew in a mid-ramp position during deposition of the Omkyk Member and are today exposed on Farm Driedoornvlakte (Figure S6)^13,22–25^. *Cloudina* individuals are found throughout the upper Omkyk Member at this locality, however the mutually calcifying, reef-building ecology is restricted to the pinnacle cap of transgressive Unit 3m^8,22,24,26,27^. Previously published palaeoredox data from Farm Driedoornvlakte were restricted to carbonate reef material^14,28^. Here, the combination of low Fe_T_ (<0.5 wt%) accompanied by intermittently negative Ce/Ce* values have been interpreted as evidence for oxic conditions during reef growth^14,28^. The combination of these data and reef construction by


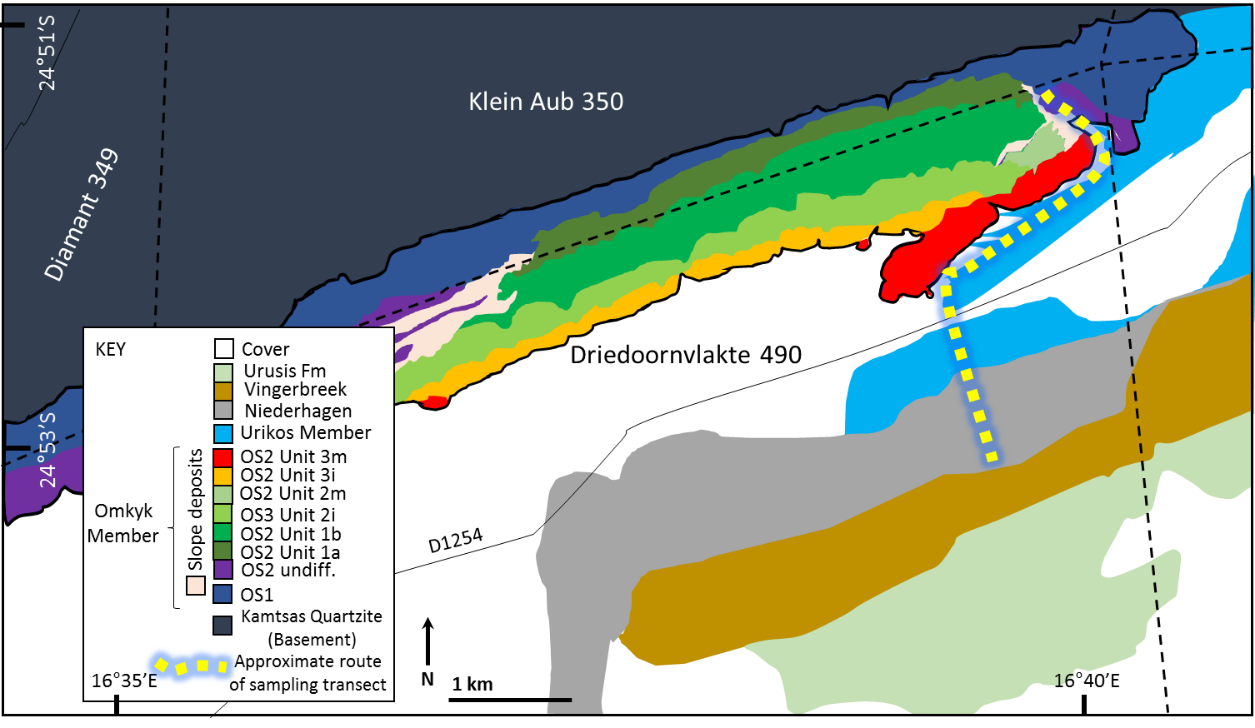


**
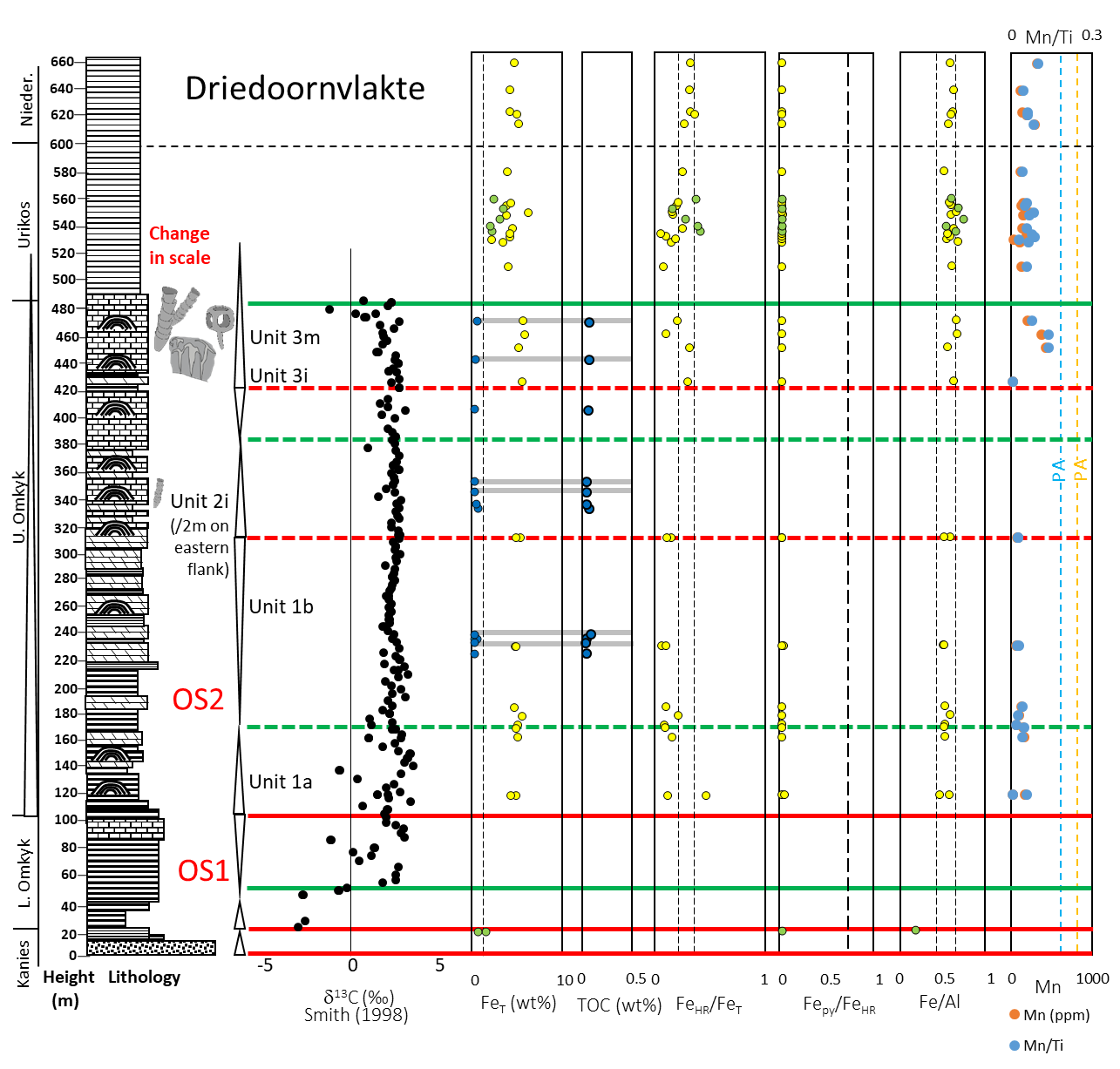
**

**
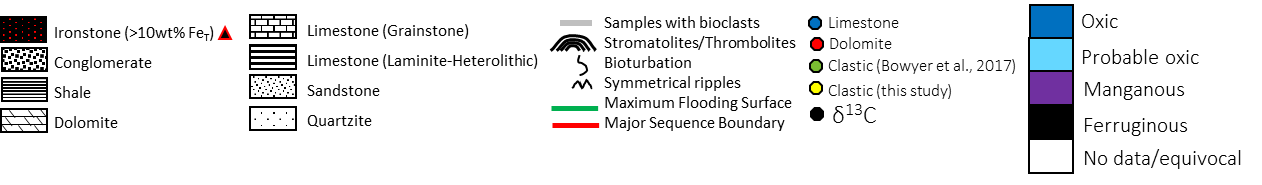
**

**Figure S6. Previous page (top).** Geological map of Driedoornvlakte reef and overlying siliciclastic deposits. Map redrawn after (^24^) and 1:250000 map of Rehoboth area (2316), Geological Survey of Namibia, Ministry of Mines and Energy.

**Figure S7.** **Previous page (bottom).** Stratigraphic log and geochemical data of composite section on Farm Driedoornvlakte (blue and green markers correspond to published data in [14] and [^29^] respectively). Section redrawn after (^24^) and (14). See above and Table S1 for legend. PA = Phanerozoic average values (after [^30^]).

*Cloudina* at this locality have supported the hypothesis that locally stable, long-lived oxia may have been a prerequisite for this unique ecological habit.

Carbonate clinoforms of the Omkyk Member interfinger with down-dip fine grained mudstones and shale at the northeast flank of the Driedoornvlakte carbonate platform (pink slope deposits on Figure S6)^24^. Structural dip of the carbonate platform and overlying stratigraphy varies between 40° and 45° to SSE, resulting in poor exposure of siliciclastic sediments of the overlying Urikos Member and Nudaus Formation across the planate topography to the south. For this study, a total of 31 shale samples were taken along a transect from the base of the Upper Omkyk Member Unit 1a, through Units 2 and 3 and, where exposed, from overlying shale of the Urikos Member and Niederhagen Member (lower Nudaus Formation) to the south of the carbonate platform (transect path in Figure S6). Siliciclastic samples of the Omkyk Member were collected from between clinoform terminations. Shale of the Omkyk and Urikos members was deposited below fair weather wave base and as such, these deposits represent the deepest sampled environment during growth of *Cloudina* at Driedoornvlakte^8,13,14,24,28,31^.

**Farm Driedoornvlakte results**

Compiled major element, Fe and P speciation data for the Driedoornvlakte bioherm are presented in Figure S7 and absolute values are provided in tables S3 – S5. During growth of the carbonate platform, Fe_T_ of down-dip shales is relatively invariant, with concentrations between 4.15 and 5.70 wt%. The stratigraphically lowest sample shows an anomalously elevated Fe_HR_/Fe_T_ value of 0.47 but overlying deposits are characterised by Fe_HR_/Fe_T_ and Fe/Al values of 0.06 – 0.33 and 0.45 – 0.64 respectively. The relative contribution of pyrite to the highly reactive iron pool is negligible throughout, with a maximum Fe_py_/Fe_HR_ value of 0.03. Shale samples of the overlying Urikos and Niederhagen members are characterised by Fe_T_ in the range 1.77 – 6.63 wt%, Fe_HR_/Fe­_T_ between 0.06 and 0.42 and Fe/Al from 0.49 to 0.71. These overlying sediments are similarly pyrite-deficient, with a maximum Fe_py_/Fe_HR_ of 0.01.

Iron speciation data of all siliciclastic samples equivalent to OS2 Units 1b – 3m firmly corroborate previous interpretations of locally stable oxia at Driedoornvlakte^14,28,29^. In this interval, 12 samples record oxic Fe-speciation values and two samples fall in the equivocal range of Fe_HR_/Fe_T_ = 0.22 – 0.38. Additionally, all samples show values of Fe/Al in the normal range for oxic Phanerozoic shale, between 0.42 – 0.64^32,33^.

The water column remained dominantly oxic during deposition of Urikos sediment following drowning of the Driedoornvlakte carbonate platform, however some evidence exists for intermittent deposition under fleeting ferruginous water column conditions in the middle Urikos Member. Two samples in this interval record moderate Fe_HR_ enrichment (Fe_HR_/Fe_T_ = 0.38 – 0.41) and one sample is typified by muted Fe_HR_ enrichment (Fe_HR_/Fe_­T_ = 0.28) but elevated Fe/Al (0.71). Regional sequence stratigraphic studies of the upper Omkyk Member confidently place deposits of the Urikos Member overlying Unit 3m at Driedoornvlakte in a down-dip position equivalent to upper Unit 3 – Urikos Member deposits in more proximal, shallower environments such as Zebra River^24,34^. This supports correlation of the possible anoxic episode overlying OS2 Unit 3m at Driedoornvlakte with anoxia at the maximum flooding surface of OS2 or the Hoogland Member at Zebra River^14,29^. These samples may thus record deposition at or near the major maximum flooding surface and proximity to ferruginous deep waters that persisted in the deeper outer ramp environment. However, the redox state of the outer ramp at this time remains unknown due to the lack of preserved outer ramp deposits of the upper Omkyk Member.

Two samples deposited subjacent to the carbonate reef of Unit 3m record the most elevated Mn/Ti values in the entire succession (Mn/Ti = 0.14) and yield Fe speciation data consistent with oxic or equivocal conditions. Average Mn/Ti of Phanerozoic marine shale is 0.185^30^ and as such, these values are relatively depleted, however the clear up-section increase in Mn concentration may be significant at Driedoornvlakte. Whilst Fe-speciation and Fe/Al are sensitive to iron reduction, E_H_ conditions that effectively oxidise Fe(II) remain conducive to Mn oxide reduction until a higher E_H_ threshold for Mn oxidation is achieved. As such, relatively elevated Mn in samples of Unit 3m, which record low Fe_HR_/Fe_T_ may be indicative of the precipitation of insoluble manganese oxides in fully oxygenated shallow waters. This is supported by relatively elevated values of Mn/Fe (0.006 – 0.009) in this interval compared to underlying and overlying deposits at Driedoornvlakte.

Early marine cements that precipitated directly within reef cavities of Unit 3m have been tentatively suggested to indicate a relatively rapid incursion of ferruginous conditions into the open *Cloudina* framework prior to disconnect from the water column by burial beneath sediment^35^. The apparent conflicting proxy records from oxic deposition recorded by Fe-speciation and anoxic precipitation of early cements may reflect: 1) the higher precipitation rate of marine cements primed to capture high frequency changes in redox, 2) that siliciclastic samples recording this short-lived ferruginous interval were missed as a consequence of poor exposure, or 3) that Fe-rich cements were precipitated during early anoxic diagenesis in open pore fluids. It is notable that the redox chemistry of shale sampled from immediately subjacent to the reef build-up of Unit 3m support deposition under E_H_ conditions adequate to fully oxidise ferrous iron and possibly manganese. At the maximum flooding surface however, the reef may have been subjected to ferruginous conditions which typified deeper waters and possibly resulted in the precipitation of Fe-rich, pore-lining isopachous high magnesium calcite cement that was later dolomitised^35^.

1. **Farm Kliphoek**

The Nudaus Formation represents a thick (>200 m) siliciclastic sedimentary succession of sandstone and shale that blankets deposits of the Zaris Formation across both the Zaris and Witputs sub-basins. Deposits of the Nudaus Formation have been largely neglected in previous redox studies. However, in addition to abundant tubular macrofossils^31,36^, the Nudaus Formation is host to fossils of soft bodied organisms including *Rangea* in the central – northern part of the Witputs sub-basin^37,38^.

In order to examine this gap in the redox record, 18 shale samples were taken from an approximately 220 m thick exposure of the Nudaus Formation, immediately overlying the undulate and locally erosive palaeotopography of the carbonate Mooifontein Member at Farm Kliphoek (Figure S8). As this section represents one of the most south-westerly exposures of the Nudaus Formation in the Witputs sub-basin, prograding shoreline deposits of the highstand systems tract (Niederhagen Member) are absent and deposits instead represent the distal, transgressive Vingerbreek Member^10,36,39^.

Olive green to dark red shales of the Vingerbreek Member (upper Nudaus Formation) on Farm Kliphoek are interpreted to represent deposition in a mid – outer ramp environment, with upward-coarsening shoaling sequences, the tops of which represent deposition above fair weather wave base^10,36,40,41^.

**Farm Kliphoek results**

Shale samples at the Kliphoek section have Fe_T_ in the range 4.94 – 29.77 wt% and Fe-speciation data are only included in the compilation for those with Fe_T_ <10 wt%. Samples with Fe_T_ >10 wt% are invariably characterised by elevated Fe/Al (up to 4.59) and these samples are herein categorised as ironstones. Extreme iron enrichments of up to 41.57 wt% have also previously been reported from some shallow water shales of the lower Aar Member^14^. Ironstones at Farm Kliphoek are interbedded with shale (Fe_T_ <10 wt%) exhibiting Fe_HR_/Fe_T_ in the range 0.10 – 0.67. Where Fe_HR_/Fe_T_ >0.38, then Fe/Al >0.64 and, as in all other analysed samples from the Nama Group, the contribution of pyrite is minimal (Fe_py_/Fe_HR_ <0.05). One sample (NUD-20) with an oxic Fe-speciation value (Fe_HR_/Fe_T_ = 0.21) at the top of the succession also records Fe enrichment from Fe/Al (0.73), which likely indicates Fe_HR_ depletion via transformation to poorly reactive sheet silicate iron (Fe_PR_) during early diagenesis^42,43^.

Iron speciation and major element concentrations at Farm Kliphoek are consistent with deposition under dominantly ferruginous conditions. However, samples with extreme Fe enrichment, alongside those showing low Fe­­_HR_/Fe_T_, may provide additional information on chemical variability in the local marine environment. Accumulation of sediments with elevated Fe_T_ in the range observed requires either, 1) early diagenetic recycling and build-up of iron oxide on the shallow shelf, or 2) a mechanism by which Fe^2+^ is rapidly and quantitatively removed from the anoxic water column at a rate greater than background deposition. In the first instance, extreme iron enrichments may have resulted from extensive and complex shallow marine sedimentary reworking via pore water sulfide oxidation coupled to iron reduction and re-oxidation. In this case, iron was likely supplied from both riverine input and intermittent shoaling of ferruginous deep waters into the oxic surface layer, however the aforementioned processes resulted in secondary augmentation of sedimentary iron concentrations. This mechanism may also account for a small number of extremely Fe-rich samples reported from the underlying lower Aar Member at Farm Arasab^14^.

**
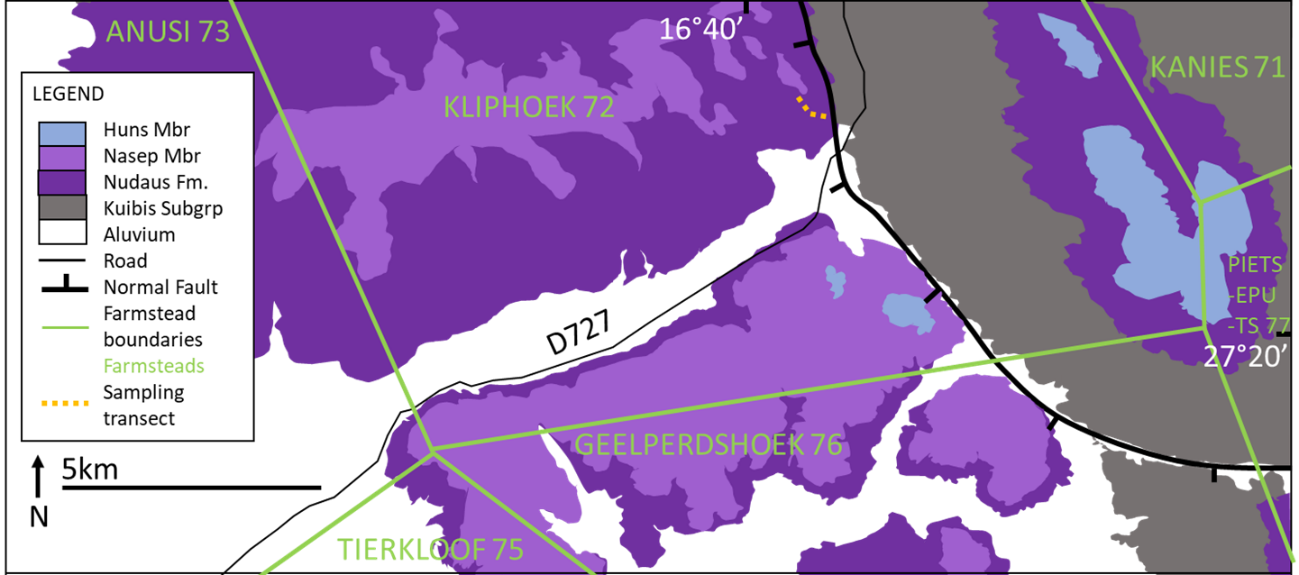

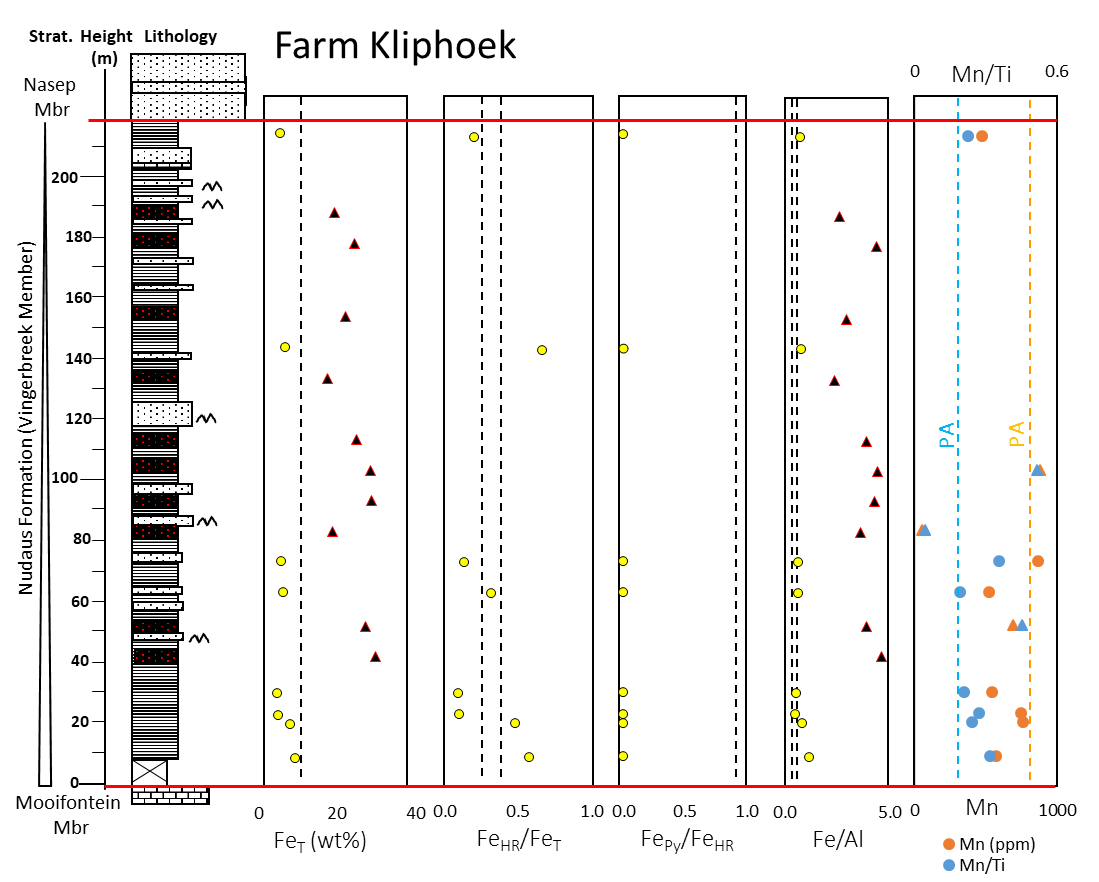

Figure S8 a)** (Top) Geological map of Nudaus Formation deposits at Farm Kliphoek showing position of sampling transect. Map drawn using a combination of (^44^, their Figure 5) and GoogleEarth, **b)** (Above) Stratigraphic log and accompanying chemical data of the Vingerbreek Member, Farm Kliphoek. See Figure S7 for figure legend. PA = Phanerozoic average.

Alternatively, hyperpycnal penetration of the redoxcline by dense, oxygenated riverine water may have intermittently promoted acute sedimentary Fe enrichment as a product of instantaneous water column Fe^2+^ oxidation. In this way, variable Fe concentrations throughout the section may either reflect parasequence-scale shoaling of the sedimentary environment or channel avulsion and associated changes in proximity to the oxidant source. A similar mechanism has been proposed for the genesis of Sturtian ironstones, whereby mixing of dense, oxic glacial meltwater permitted long-lived Fe oxide deposition of the Holowilena ironstone^45^.

The localised and short-lived return to ferruginous conditions in the Witputs sub-basin during deposition of the Nudaus Formation is associated with extreme values of CIA (up to 88%, see section 6) and shale deposited atop palaeovalleys of the ‘Vingerbreek unconformity’, known only from the Witputs sub-basin in the Klein Karas Mountains (and elsewhere) to the east of Farm Kliphoek^46^. This localised unconformity has been attributed to a regional glacio-eustatic change in sea-level and undoubtedly records an interval of enhanced continental denudation. Ferruginous beds are also associated with this possible glacial unconformity^46^. Palaeocurrent information demonstrates a distinct provenance for sediments of the Vingerbreek Member between the Zaris and Witputs sub-basins, with supply of detrital material to the Zaris sub-basin from the north and to the Witputs sub-basin from the east^8^ (Figure S13c). If the Vingerbreek unconformity is indeed glacial in origin, then extreme sedimentary Fe enrichments in deposits of the Vingerbreek Member may have arisen from enhanced chemical weathering and nutrient delivery associated with post-glacial transgression and rapid chemical weathering of glacial rock flour. Localised nutrient input, supplied by this short-lived sub-basin scale denudation event may help explain the protracted transition to stable, oxic mid-depths south of the Osis arch.

1. **Schwarzrand Subgroup north of Farm Osis**

Fossils of soft-bodied forms including *Shaanxilithes* (or, alternatively, *Palaeopasichnus*, Zhuravlev, A.Yu. pers. com.) and *Aspidella* have recently been reported from shallow marine siliciclastics of the lower Schwarzrand Subgroup north of Osis^47^. These finds have re-emphasised the importance of widely neglected Zaris sub-basin deposits for accurate palaeoenvironmental reconstruction of the Upper Nama Group across the entire Nama basin.

During deposition of the Schwarzrand Subgroup, gradual siliciclastic infill of the Zaris Sub-basin shifted the locus of carbonate sedimentation to the shallow marine environment south of the Osis arch^8,9^. The Schwarzrand Subgroup north of Osis covers an almost entirely planate areal expanse of approximately 8000 km^2^, however the combined lack of relief and burial beneath deeply weathered regolith results in a scarcity of vertically exposed sections. Where exposed, sedimentary features are commonly representative of prodeltaic and shoreline shallow marine deposition above storm wave

**
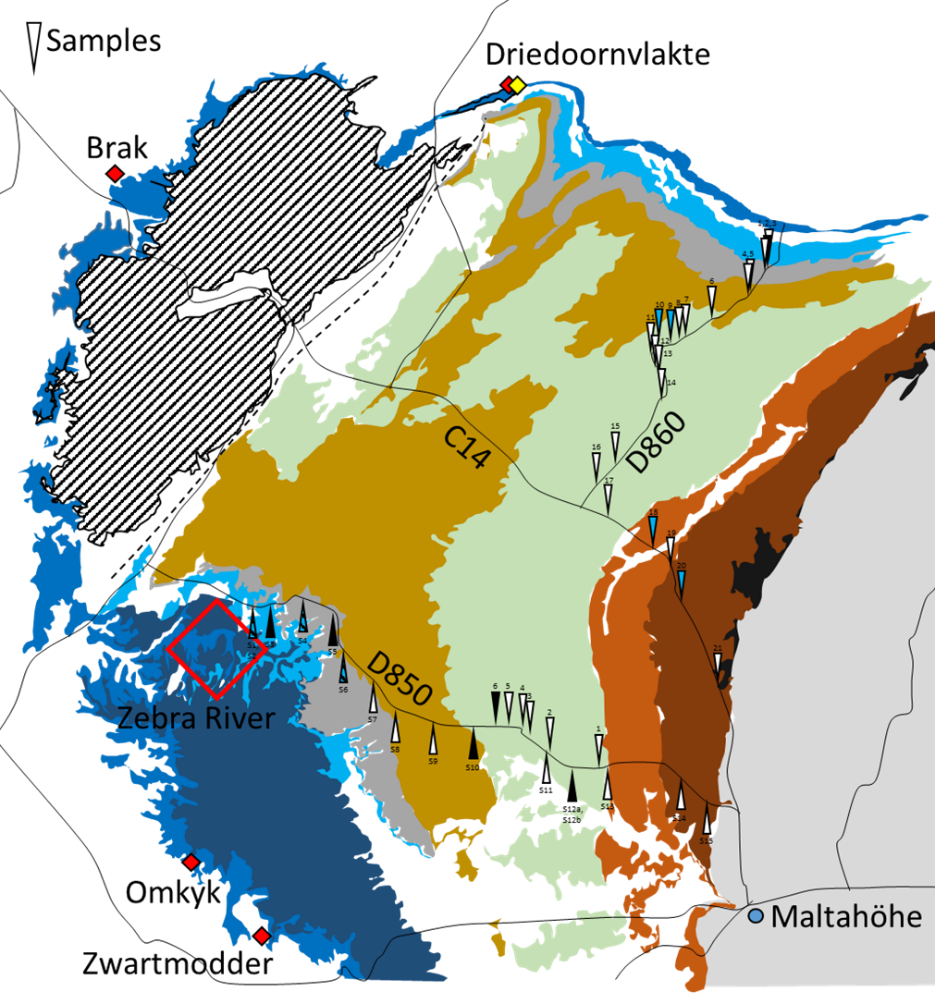
**

**Figure S9** Geological map of the Schwarzrand Subgroup north of Osis with relative positions of individual sampling localities. Map redrawn using a combination of GoogleEarth and geological maps (1:1000000, 1963, 1978, and revised 1980 editions, and map of Mariental area, 2416, Geological Survey of Namibia, Ministry of Mines and Energy).

base, frequently interrupted by channelized sandstones. However, some areas continue to record sedimentation in offshore settings of low – intermediate hydrodynamic energy, conducive to microbial mat growth (e.g. ^48^). Prior to sub-basin infill, relative palaeodepth is thought to have increased with distance north from the Osis Arch and west from the Kalahari Craton and as such, sequential exposure of higher stratigraphic units towards the southeast corresponds to a relative shallowing^9,49^.

Shale samples were collected along three road transects north of Osis (D860, C14 and D850, figures S9, S10) that bisect Member boundaries at known distances across a total sampling interval spanning the Urikos Member of the upper Kuibis Subgroup to the Niep Member (Nomtsas Formation) of the Schwarzrand Subgroup. The relative positions of sampling localities were measured by GPS and plotted using Google Earth overlain by the 1:250000 Geological Survey of Namibia (Ministry of Mines and Energy) map of Mariental (2416) and samples were placed into their relative stratigraphic position (figures S9, S10). This sampling was conducted with the aim of supplementing the preliminary redox study of (^29^) and as such, the combined dataset is discussed herein.

**Schwarzrand Subgroup results**

Diachronous deposition of Urikos Member sediments incrementally swamped shallow carbonate production of the Omkyk and overlying Hoogland members during long-lived transgression of the upper Zaris Formation in the Zaris sub-basin^50^. As such, the Urikos Member represents the structurally lowest unit and deepest palaeoenvironment sampled along these road transects. In samples of the Urikos Member, total Fe concentrations range from 3.56 to 6.68 wt% (mean = 5.11 wt%), Fe_HR_/Fe_T_ values are in the range 0.15 to 0.69 (mean = 0.37) and Fe/Al falls between 0.64 and 0.86 (mean = 0.73). Only where Fe_HR_/Fe­_T_ >0.27 is minor iron enrichment corroborated by Fe/Al >0.64.

The overlying Niederhagen and Vingerbreek members (Nudaus Formation) have Fe_T_ in the range 2.58 to 4.42 wt% and Fe_HR_/Fe_T_ between 0.22 and 0.45. However, of the four samples where Fe_HR_/Fe_T_ >0.38, only one shows significantly elevated Fe/Al >0.64. Where these proxies appear to disagree, Fe_HR_/Fe_T_ does not exceed 0.39, suggesting minor Fe_ox_ enrichment in a dominantly oxic water colum.

Urusis Formation samples have Fe­_T_ between 1.64 and 5.87 wt% and Fe_HR_/Fe_T_ between 0.14 and 0.42. Five samples with Fe_HR_/Fe­_T_ between 0.22 and 0.38 have elevated Fe/Al (0.67 – 0.78) that may imply late stage conversion of highly reactive to poorly reactive sheet silicate-bound iron.

Finally, distal fluvial to shallow marine sediments of the Nomtsas Formation (Kreyrivier and Niep members) have Fe_T_ between 2.50 and 5.23 wt%, Fe_HR_/Fe_T_  values of 0.21 – 0.30 and Fe/Al in the range 0.47 – 0.62. In the Nomtsas Formation, normal marine Fe/Al data corroborate low Fe_HR_/Fe_T_ (<0.22) in two samples and imply negligible water column Fe enrichment for the remaining three.

Mn/Ti and Mn/Al are almost all below average shale values^30^, however there is a notable shift in Mn concentrations (reflected in Mn/Ti and Mn/Al) up-section (Figures S10 and S11). Initially, samples are extremely depleted with respect to Mn, with a minimum concentration of 62 ppm (Mn/Ti = 0.052) in the Niederhagen Member and concentrations progressively increase to a maximum of 590 ppm (Mn/Ti = 0.21) in the lower Nomtsas Formation, before decreasing slightly to a final value of 418 ppm in the upper Nomtsas Formation. There is a positive correlation between Mn/Ti (r^2^ = 0.544), P/Ti (r^2^ = 0.564) and Fe/Ti (r^2^ = 0.648) in Zaris sub-basin samples of the Schwarzrand Subgroup, reflecting a dominantly detrital source for these elements. However, the positive up-section trends in Mn/Ti and P/Ti (Figure S11) and their negligible covariation with Ti/Al support a mechanism for relative sedimentary Mn and P enrichments (/retention) that are disconnected from detrital input and bulk contribution in clay minerals. This is further supported by a pronounced increase in Mn/Fe up-section through the Schwarzrand Subgroup, with no associated trend in Fe concentration or Fe/Al (see below).

Samples of the Schwarzrand Subgroup in the Zaris sub-basin display relatively invariant Fe_HR­_/Fe_T_, which are largely confined between the calibrated threshold ratios of Fe_HR_/Fe_T_ = 0.22 – 0.38. In the upper Schwarzrand subgroup, the Witputs sub-basin was freely-connected to the south and here Fe-**
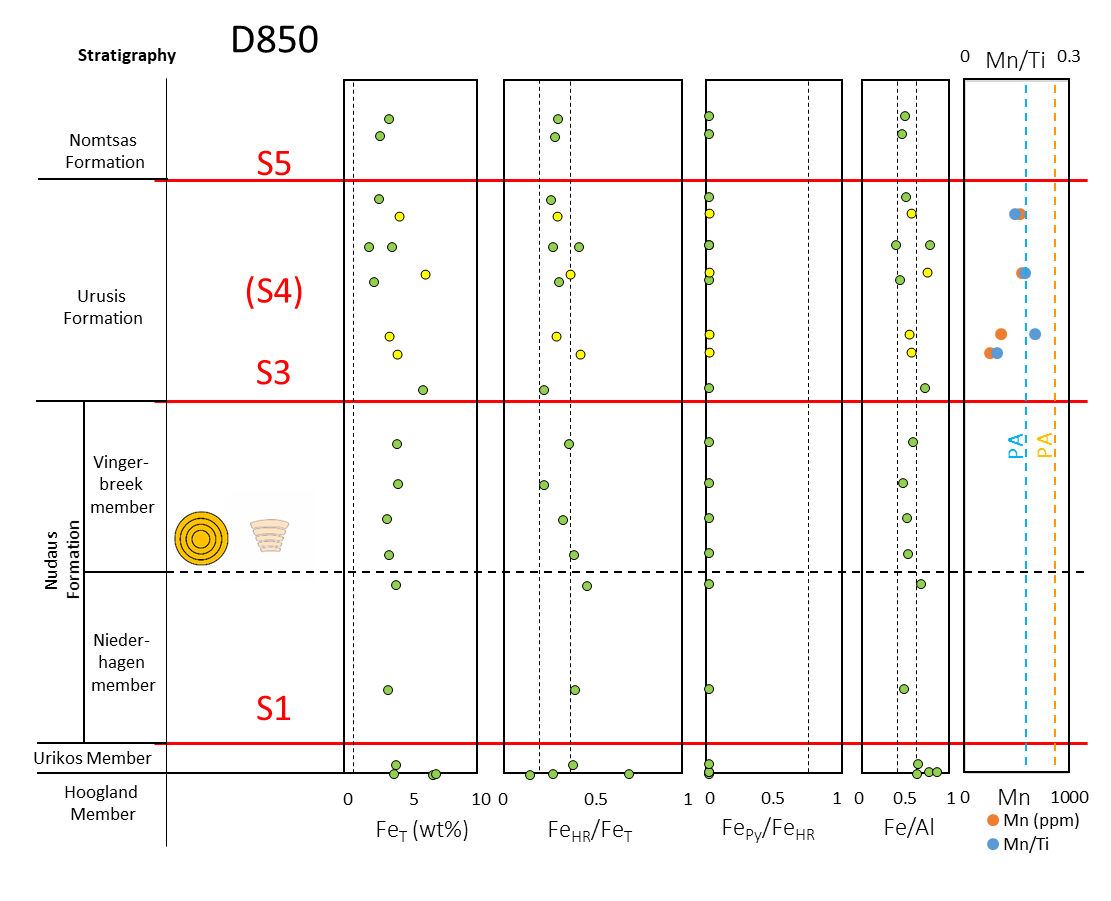

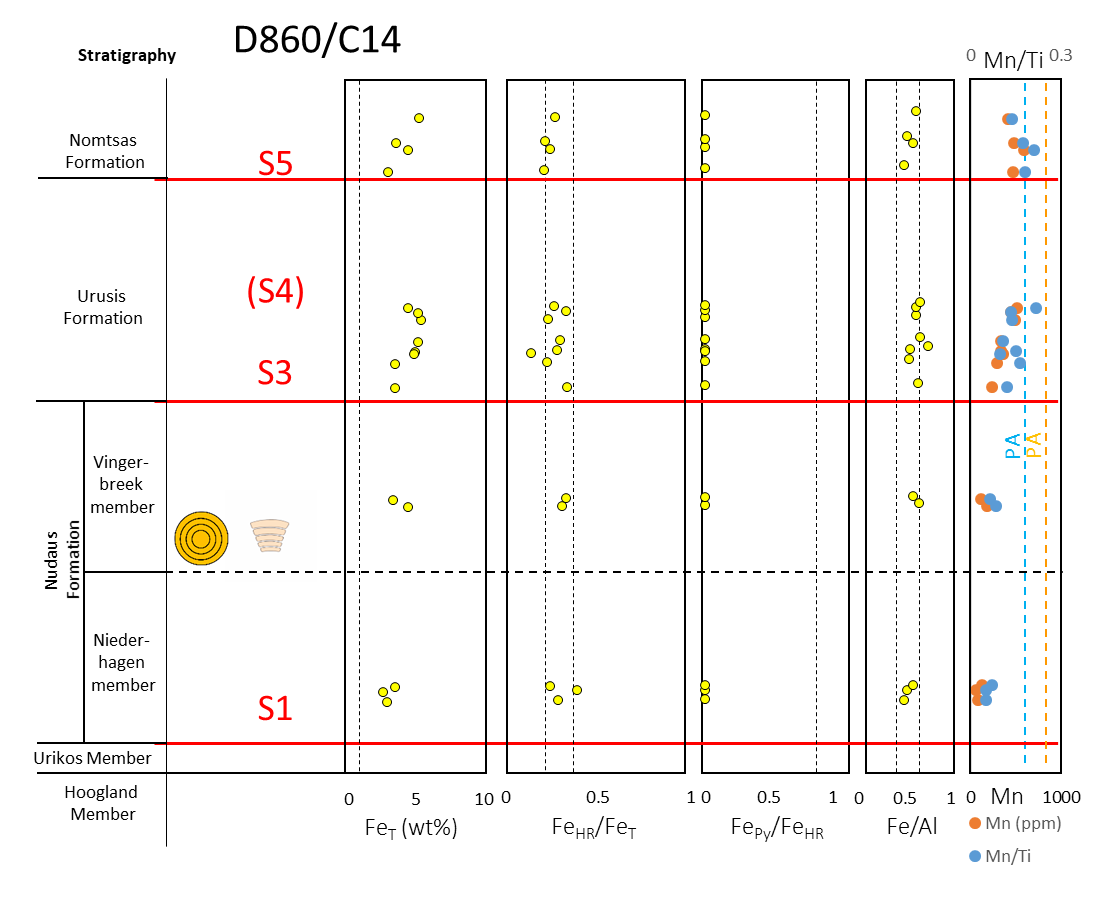
**

**Figure S10 (Previous page)** Schematic sections for samples collected along the **(a)** D850 and **(b)** D860/C14 road transects. Data from samples in green are published in (^29^) and those in yellow are new data. See Figure S7 and Table S1 for legend. for key to fossil symbols. PA = Phanerozoic average.

speciation data indicate unequivocally stable oxic water column conditions^14^. Contemporaneous shale interbeds of the Zaris sub-basin represent deposition in a mildly productive inner ramp environment with abundant siliciclastic biolaminites, organic walled microfossils, *Vendotaenia* and burgeoning evidence for primitive burrowing organisms^31,47,51,52^**.** Under lower Ediacaran atmospheric and marine oxygen concentrations, organic matter remineralisation in shallow, stagnant and moderately productive inner ramp environments may have promoted occasional pore water ferric (oxyhydr)oxide reduction and re-oxidation, leading to intermittently elevated Fe_HR_/Fe_T_. However, the observed positive trend in Mn/Ti (and Mn/Fe) towards an apex in the Schwarzrand Subgroup at values typical for Phanerozoic oxic shale (0.185^30^), supports a gradual increase in oxygenation (or decreased influence from anoxic deeper waters) to a level conducive to sedimentary Mn oxide retention.

An alternative interpretation of the combined data would view the majority of samples in the Schwarzrand Subgroup north of Osis that fall in the equivocal range of Fe_HR_/Fe_T_ (0.22 – 0.38), and the normal marine range for Fe/Al (0.44 – 0.66), as predominantly reflecting dilution of an anoxic signal by rapid deposition^32,33,53^. The inherent difficulty in disentangling these processes is particularly apparent during infill of the Zaris sub-basin in the lower Nomtsas Formation. However, the noted positive shift in Mn/Fe towards the top of the Schwarzrand Subgroup likely attests to trapping of manganese oxides, which became more efficient in the oxic portion of the water column up-section as a consequence of progressive shallowing of the depositional environment.


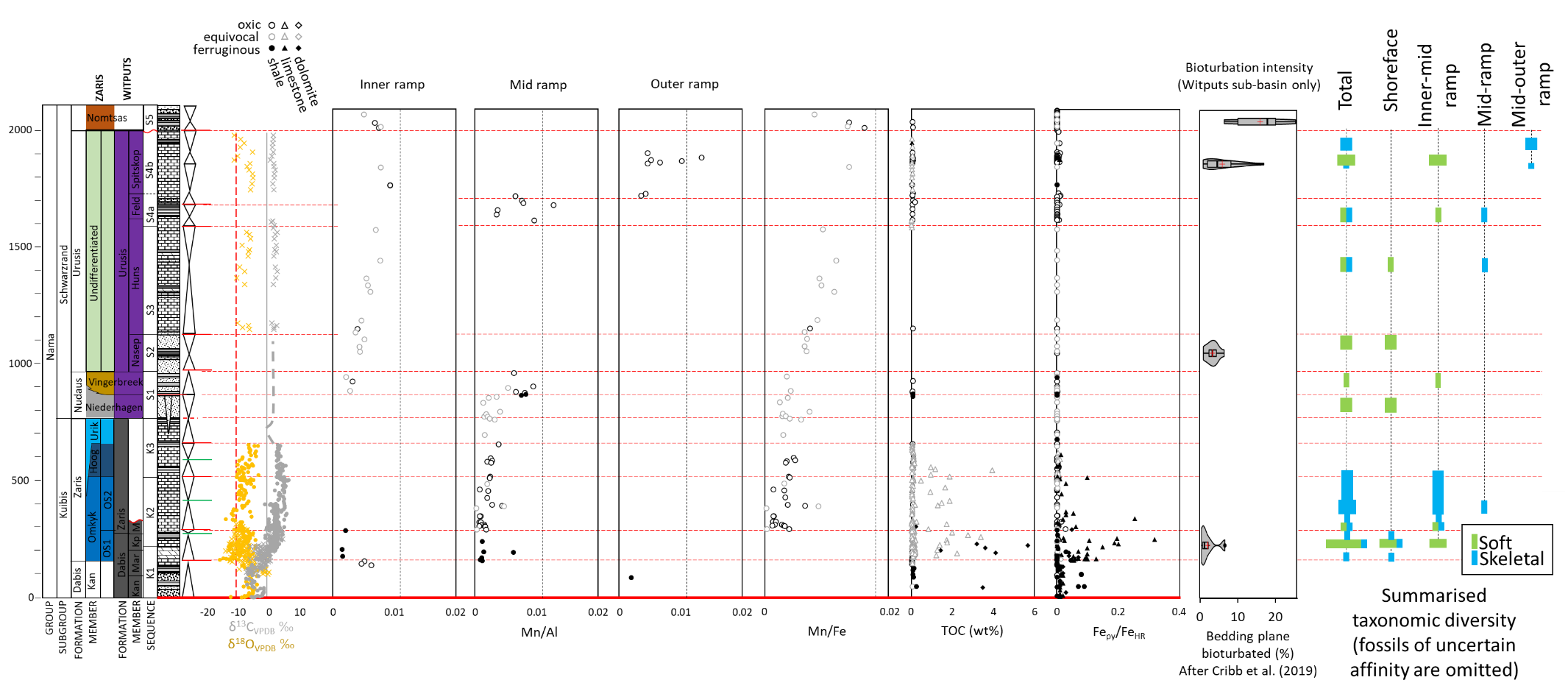


**Figure S11** Compiled data of Mn/Al, Mn/Fe, TOC and Fe_py_/Fe_HR_ for Nama Group samples. Symbols correspond to lithology and redox interpretation (after Fe-speciation). Scale conforms to composite Nama Group section of (11) (Their Figure 6A). Vertical dashed lines indicate Phanerozoic average compositions after (30). Symbols in composite carbon isotope profile conform to main text Figure 2. Summarised taxonomic diversity compiled in Table S1 and Figure S12. Violin plots of bioturbation intensity after (54).

1. **Biotic Distribution in the Nama Group**

There is a pivotal transition towards low and stable Fe_HR_/Fe_T_ from ~547 Ma and concomitant increase in bioturbation intensity (main text, Figure 2). The Kuibis Subgroup in the Witputs sub-basin (Figure S13a) yields abundant soft-bodied organisms of the classic Nama assemblage (Figure S12) including *Ernietta*, *Pteridinium*, *Rangea*, and *Ausia* in addition to *Aspidella*, *Namalia* (possibly synonymous with *Ernietta*) and the problematica *Buchholzbrunichnus* and *Orthogonium* (see table S1)^20,37,55–61^. *In situ* specimens are largely confined to wave-influenced shoreface sandstones. However, localised occurrences of *Rangea* and *Ernietta* have also been recorded from settings inferred as deeper inner- to mid-ramp (between fair-weather and storm wave base) in the Upper Kliphoek (Aar) Member^60,61^. Shallow carbonates of the Mara Member, coeval with fossiliferous clastics, locally host the earliest reported *Cloudina*^8^ and inner ramp limestone interbeds of the upper Kliphoek Member contain *Namacalathus* (Figure S12), where very low Fe_T_ (<0.5 wt%) has been suggested to represent probable oxygenated conditions suitable for opportunistic colonisation^14^. Organic walled microfossils, including the possible cyanobacterium *Bavlinella* *faveolata* are also reported from borehole material of the Kliphoek Member^31^.

In the Zaris sub-basin, the upper Kanies and lower Omkyk members are time-equivalent deposits to the Mara and Kliphoek members^11^ but have, to date, yielded no fossils (Figure S12). However, the upper Kuibis Subgroup of the Zaris sub-basin (Figure S13b) contains both *Cloudina* and *Namacalathus* in inner to mid ramp environments and large microbial-metazoan reefs grew unimpeded in persistently oxic mid-ramp positions^14,23,26,62^. *Palaeopasichnus* (see above) and one putative specimen of *Gaojiashania*^63^ are noted from the upper Omkyk and lower Hoogland members respectively. This interval is also characterised by the first appearance of possible complex surface traces in the Nama Group from inner to mid ramp facies^21^, although an ichnofossil affinity has been questioned^64^ and this specimen may instead represent a palaeopasichnid^21^. If the latter is true, then trace fossil diversity in the Kuibis Subgroup was extremely low and represented exclusively by simple horizontal traces of small size, constrained to intertidal to shallow subtidal siliciclastic facies^8,51,65,66^.

The Nudaus Formation in the Zaris sub-basin (Figure S13c) records deposition in a mildly productive, shallow inner ramp environment with abundant siliciclastic biolaminites^52^. Here, a low diversity assemblage contains examples of *Aspidella*, *Shaanxilithes* (or, alternatively, *Palaeopasichnus*), acritarchs and filamentous *Vendotaenia* (Figure S13c – d)^8,31,36,38,47,51^. In the Witputs sub-basin, shallow transgressive deposits of the Nudaus Formation (Niederhagen Member) contain *Rangea*^37^, whilst deeper facies of the conformable Vingerbreek Member are host only to simple, tubular macrofossils that superficially resemble *Vendotaenia* and organic walled microfossils including *Bavlinella* *faveolata* (Figures S13c)^8,36^.

No fossils are reported from Urusis Formation deposits in the Zaris sub-basin. This, however, may largely be a consequence of sampling bias due to relatively poor exposure. By contrast, Urusis Formation deposits in the Witputs sub-basin contain body fossils of *Nasepia*, *Swartpuntia*, *Pteridinium*, *Aspidella*, possible *Bradgatia*, and biomineralising *Cloudina* and *Namacalathus*^14,67,68^. Additionally, the lower Schwarzrand Subgroup of the Witputs sub-basin records a notable increase in average bioturbation intensity as represented by the percentage of bedding plane bioturbated^54^, and here, Urusis Formation deposits include the earliest treptichnids^69–71^ and localised occurrences of complex sediment bulldozing habits^72^. As with examples from the Kuibis Subgroup, soft-bodied and trace fossils of the Urusis Formation are commonly preserved in shallow, coarse clastics representing shoreface deposits. However, the most distal fossiliferous section in the upper Spitskop Member (Farm Swartpunt) represents a succession of mid to outer ramp facies within which variably-transported coarse delta-front sandstones contain soft-bodied forms^67,68,73,74^. At Swartpunt, complex trace fossils (*Streptichnus narbonnei*) are recorded from siliciclastics interbedded with layers of thin, outer shelf limestone that contain *in situ* *Cloudina* and *Namacalathus*^14,70^.

**
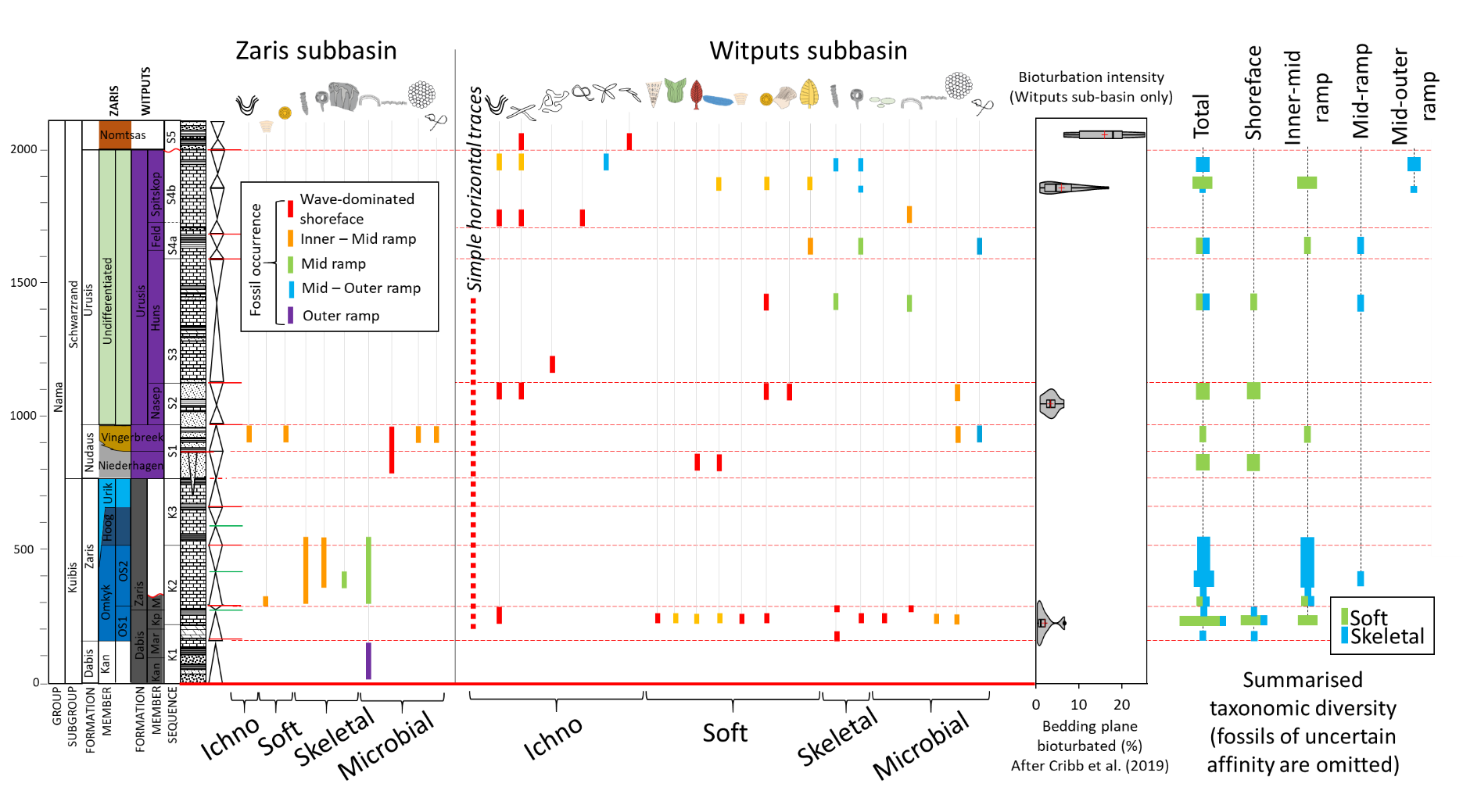
**

**Figure S12** Published Nama Group fossil occurrences. Violin plots of bioturbation intensity after (^54^). Fossils of uncertain affinity are omitted (full details in Table S1).

**
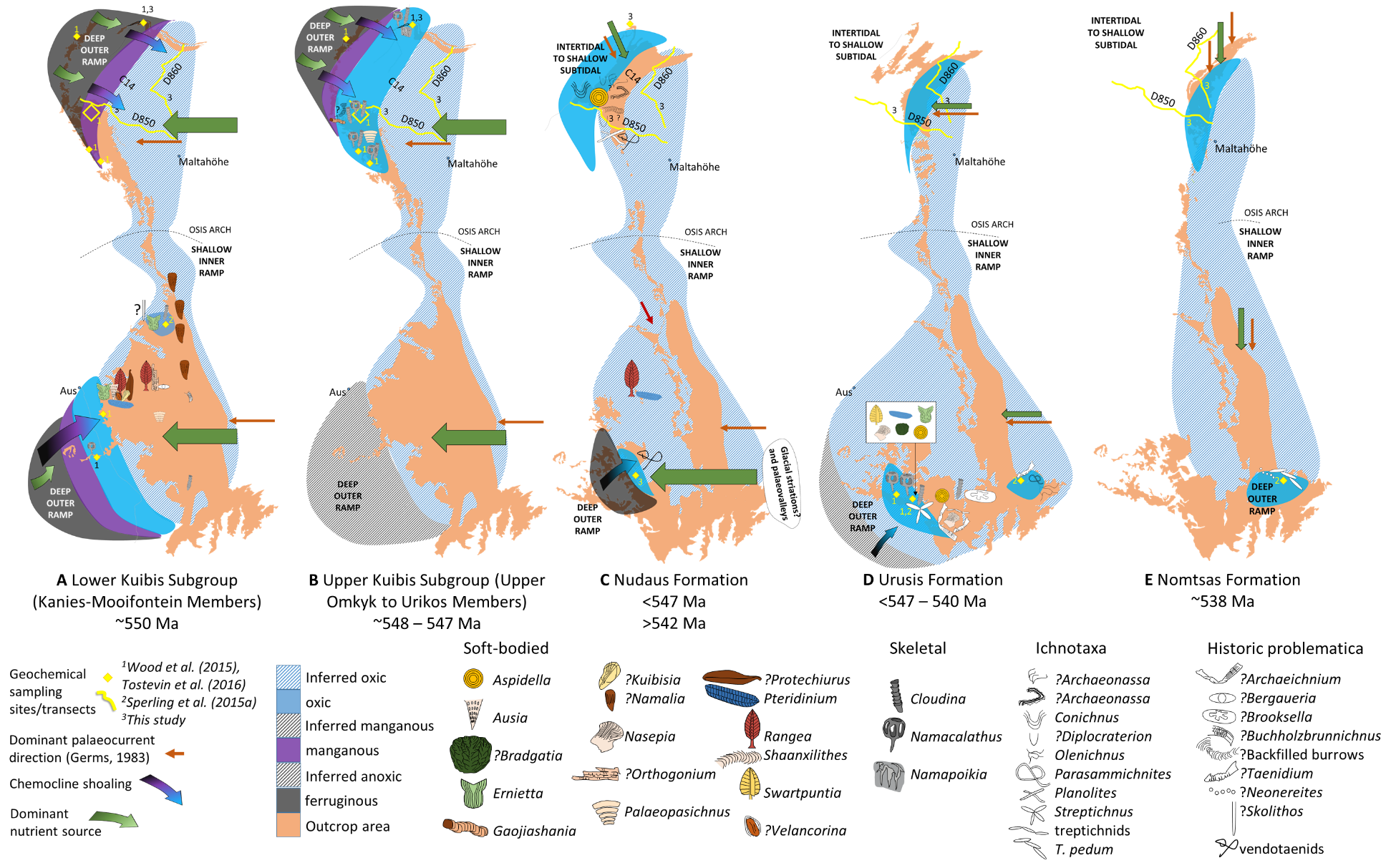
**

**Figure S13** (Previous page) Schematic diorama representing the plan-view evolution of redox in the Nama Group, Namibia, over ~12 Myr showing the evolution of redox, and distribution and diversity of biota. Fossil occurrence information and uncertainties provided in Table S1. Please note, question marks denote high degrees of uncertainty in fossil affinity (see Table S1).

1. **Nama Group palaeoecology**

Figure S12 shows the distribution of fossil forms recorded in the Nama Group (detailed in Table S1). The vast majority of Ediacaran macrofossils cannot yet be confidently assigned to the Metazoa^75–77^ and therefore the assumption that significant dissolved oxygen was required by many representatives of the Nama assemblage is questionable. This is exemplified by recent biomarker evidence supporting a colonial cyanobacterium affinity for *Nemiana*^78^ (a junior synonym of *Beltanelliformis*^79^), a former candidate metazoan. Indeed, of all the organisms represented in the Ediacaran body fossil record only a handful share attributes representative of animal-grade complexity, most notably the motile bilaterians *Kimberella* and *Dickinsonia* both of which are restricted to siliciclastics of the White Sea assemblage^80–83^, which is thought to precede the Nama assemblage with a degree of overlap. Whilst likely animal representatives, including the calcifying forms *Cloudina*, *Namacalathus* and *Namapoikia*, may have required at least moderate levels of oxygen to sustain a skeletonising growth habit^14,28,84–86^, inferring habitable oxygen thresholds for the enigmatic soft-bodied assemblage preserved in Nama sediments may currently be considered phylogenetically unsubstantiated. However, the persistent spatial separation of anoxic deep waters from habitable ecospace implied by the fossil distribution of soft-bodied forms in the Nama Group may support the inference that at least minor oxygen concentrations were a metabolic requirement of these organisms. Of all soft-bodied fossils in the Nama record, only *Pteridinium*, *Rangea*, *Ausia* and one tentative specimen interpreted as *Gaojiashania* have been proposed to represent possible stem-group animals and, even then, phylogenetic interpretations remain highly controversial^63,87–90^. By contrast, it is widely acknowledged that active metabolisms maintained by bilaterian ichnofossil trace makers are more robustly interpreted as having required elevated levels of dissolved O_2_ ^71,84,85,91^.

The rise of a widespread sediment-disturbing life habit may also have had negative implications for pre-existing benthic and immobile suspension feeding fauna, largely associated with a loss of stable substrate and obstruction of filter feeding appendages^72^. It has previously been proposed that the possible semi-infaunal life habit exploited by some organisms of the Nama assemblage, including *Ernietta* (/*Namalia*) and *Pteridinium* may thus have been largely abandoned as a consequence of the significant substrate impact of emerging bioturbators^57,92,93^. This would be particularly pertinent following the development of shallow-infaunal burrowing such as that represented by the recently-described *Parasammichnites pretzeliformis* in lower Spitskop Member sediments of the Witputs sub-basin^72^.

It is tempting to consider the additional impact of P retention associated with the rise of sediment-disrupting organisms. Caution has been justly advocated in basing interpretations of evolutionary patterns in bioturbation intensity solely upon published occurrence information^94^. A detailed, systematic sampling approach may improve the resolution of the ichnofossil record in the Nama Group through quantitative characterisation of ichnofabric index^95^ and bedding plane bioturbation index^96^. In the Schwarzrand Subgroup, trace fossil occurrences are spatially limited and often represented by discreet burrows and shallow mixed layer depths (max 0.5 cm), likely equating to an average ichnofabric index (ii) no greater than 2^94,95^. Localised shallow marine siliciclastic bedding planes of the lower Spitskop Member host the earliest recorded sediment bulldozing traces (*P. pretzeliformis*)^72^ and similar facies of the overlying early Cambrian Nomtsas Formation host abundant treptichnid traces where, in rare instances, bedding planes show an (ii) of up to 5, indicating episodically intense sediment mixing^71^. In fact, a recent study concerned with the Nama Group ichnofossil record has quantitatively shown a progressive increase in mean bioturbation intensity (as a function of % bedding plane bioturbated) from 1.94% in the Kliphoek Member, 3.39% in the Nasep Member, 5.83% in the Spitskop Member, and culminating in a mean value of 16% for studied sediments of the Nomtsas Formation^54^. Despite this notable increase in average bioturbation intensity in the Nama Group, the potential influence of bioirrigation and mixing-induced sedimentary phosphorus retention on local water column productivity remains unclear and may benefit from further quantitative assessment.

1. **The Chemical Index of Alteration**

Chemical weathering plays a major role in controlling the mineralogical composition of fine grained siliciclastic rocks and is therefore a key metric in understanding compositional changes in their major element distribution, particularly in relation to the regional supply of nutrients from the continent and the maturity of terrigenous clay. During chemical weathering, labile elements are preferentially removed, resulting in elevated ratios of immobile elements (Al, Ti) to alkalis (Na, Ca, K)^97^. The chemical index of alteration (CIA)^97^ has long been used to assess the degree of chemical weathering required for an observed suite of major elements and is derived as:

CIA = [Al_2_O_3_/(Al_2_O_3_ + CaO* + Na_2_O + K_2_O)] x 100

When calculating CIA, the CaO concentration in silicates (CaO*) is evaluated by correcting for Ca contribution in carbonate and apatite^97^. Available P_2_O_5_ data was used in order to first correct for Ca in apatite (CaO_corr_ = CaO – 10/3 x P_2_O_5_, concentrations in mol%)^98,99^. Where resultant CaO_corr_ in moles was less than Na_2_O, CaO_corr_ was used as CaO*. Conversely, where CaO_corr_ > Na_2_O, the CaO* value was determined through equivalence with the average CaO/Na_2_O ratio of the sample set^99,100^.

Additional concern surrounds the diagenetic accumulation of K via potassic metasomatism, which is corrected for by projecting observed K excess onto a theoretical weathering trend in A-CN-K (Al_2_O_3_ – [CaO* + Na_2_O] – K_2_O) space^98,99,101–103^. Nama Group strata have undergone no greater than

**
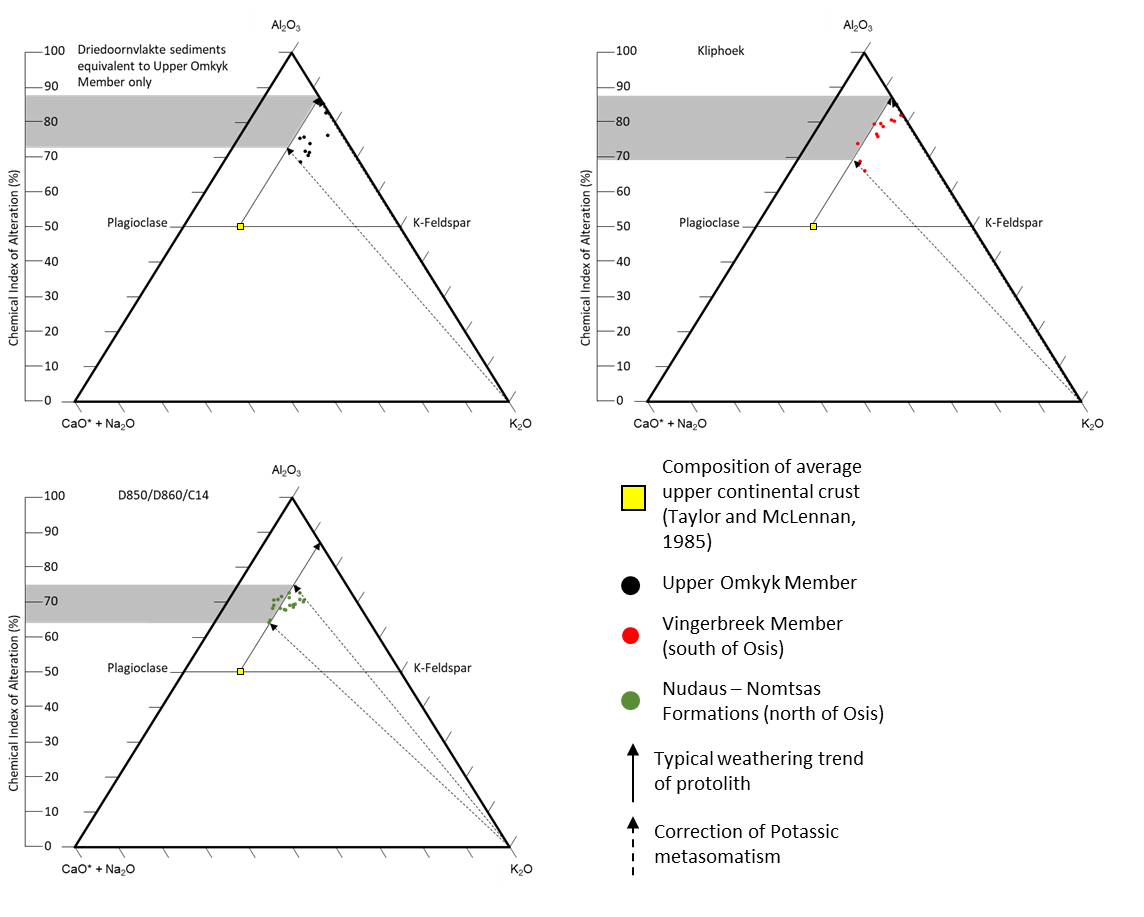

Figure S14.** Calculated values of the chemical index of alteration and the relative positions of sampled areas in A-CN-K space.

zeolite facies metamorphism (thermal alteration in the range 170 - 200°C, [^31^]), therefore deviation from the weathering trend toward the K_2_O apex likely reflects K contributed during clay diagenesis. The resulting range of CIA values fall between ~59 – 88% with the highest values confined to the Kuibis and lower Schwarzrand subgroups as previously observed (Figure S14)^103^. The overlapping range of CIA values in samples of the Upper Omkyk Member at Farm Driedoornvlakte and the Vingerbreek Member at Farm Kliphoek reflect their provenance from the Kalahari craton to the present east^8^. By contrast, the narrower range and lower average value of samples of the Schwarzrand Subgroup of the Zaris sub-basin are likely a product of sediment input from the less heavily weathered protolith to the north in addition to that subsequently supplied from the east (upper Schwarzrand Subgroup), consistent with published palaeocurrent and heavy mineral data^8,103^.

| **Table S1**: Nama Group fossil information and occurrence catalogue | | | | | | |
| --- | --- | --- | --- | --- | --- | --- |
| **Ichnofossils and putative body fossils. Body fossils of uncertain affinity (UA) are not included in summarised taxonomic diversity estimates of figures 2, S11 or S12.** | | | | | | |
| **Symbol** | **ID (red: not included in diversity compilation)** | **Notable occurrences** | **Stratigraphic Position** | **Sedimentological context** | **References** | **Affinity/Notes** |
| **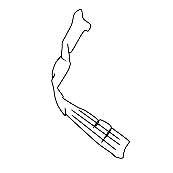** | *Archaeichnium* | Witputs sub-basin, Farms Arimas and Groendorn (near Ham River). Zaris sub-basin, Farm Harughas. | Lower and middle Huns Member. Vingerbreek Member. | Wave-rippled fine sandstone with siliciclastic biolaminites. Transgressive sequence. Interpreted as moderate energy between fair weather and storm wave base. | ^48,69,104–106^ | **UA.** Probable tubular body fossil^105^ |
| 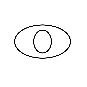 | *Bergaueria* | Witputs sub-basin, Farm Kuibis | Kiphoek/Urikos Member | Grey, slightly scoured sandstone bed. Transgressive sequence. Moderate energy environment. | ^51^ | **UA.** May be *Intrites* in the Nama Group^107^. Probable MISS^108^. |
| **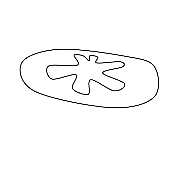** | *Brooksella* | Witputs sub-basin, Farm Huns. | Nasep Member. Urusis Formation. | Plaster cast only. Algal reticulate patter noted on specimen. Shallow intertidal environment of deposition. Farm Huns. | ^51^ | **UA.** Possible pseudo- fossil after (^109^). If *Brooksella*, *then* possible sponge after (^110^) |
| **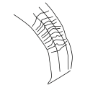** | *Buchholzbrunnichnus*  *kroeneri* | Witputs sub-basin, Farm Buchholzbrunn | Kliphoek/Urikos Member |  | ^58^ | **UA.** Reinterpreted as body fossil of unknown affinity after (^70^). **Possible *Corumbella*?** |
| **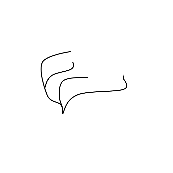** | Possible *Chondrites* | Zaris sub-basin, Farm Kuderup | Vingerbreek Member | Muddy tidal facies. | ^51^ | **UA**. Possible *Archaeonassa* after (^70^) or coinciding horizontal traces after (^109^) |
| **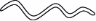** | *Cochlichnus* | Witputs sub-basin, Farm Arimas | Lower Huns Member | Sandstone-mudstone. Inner-mid ramp. | ^70^ |  |
| **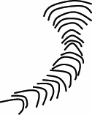** | Complex backfilled burrows | Zaris sub-basin, Hauchabfontein Farm | Upper Omkyk Member | Quartz-rich sandstone lenses within trough cross-bedded limestone. | ^21^ | **UA.** Problematic interpretation as ichnofossil^64^. Possible *Palaeopasichnus*^21^. |
| 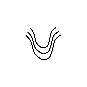 | *Conichnus* | Zaris sub-basin, Road transect (D850 and D855), Witputs sub-basin, Farm Arimas | Vingerbreek Member, Huns Member | Fine grained, rippled sandstone. Shallow subtidal. | ^47,70^ |  |
|  | Curved burrows | Zaris sub-basin, east of Farm Driedoornvlakte | ‘Basal clastic member of the Schwarzrand Formation’ (Urikos Member of Zaris Formation) | Mid-ramp limestone. | Plate 1, Figure 4 of (^106^) | **UA. Specimens described and figured are *Namacalathus***. |
| 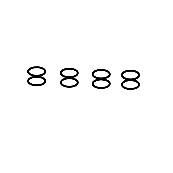 | *Diplichnites* | Witputs sub-basin,  Farm Vergelee (bordering Sonntagsbrunn to SE) | Nomtsas Formation | Sandy tidal facies. | ^51^ |  |
| 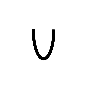 | *Diplocraterion* | Zaris sub-basin, Farm Haruchas, Farm Berghoek, Farm Kuderup | Niederhagen and Vingerbreek members | Sandy tidal facies. | ^38,51^ | **UA.** Possibly more simple than *Diplocraterion*^25,70^ |
|  | *Helminthoidichnites* | Zaris sub-basin, Farm Urusis.  Witputs sub-basin, Farms Swartpunt, Arimas, Canyon Roadhouse | Zaris sub-basin: Urusis Formation. Witputs sub-basin: Nasep Member, Spitskop Member |  | ^54^ | Described in (^54^) |
| 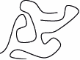 | *Helminthopsis* | Witputs sub-basin, Farms Arimas, Swartpunt | Lower Huns Member, (Sepcifically in Nasep and Spitskop members of Witputs sub-basin) | Sandstone-mudstone. Inner-mid ramp. | ^54,70^ | Described in (^54^) |
| 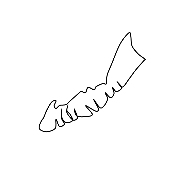 | *Muensteria isp.* | Witputs sub-basin, Farm Arimas, Farm Sonntagsbrunn | Nasep Quartzite Member (/lower Huns Member), Feldschuhhorn Member | Sandstone-mudstone. Inner-mid ramp. | ^106^ | **UA. Synonymous with *Taenidium* and *Scolecocoprus*. Probable cast of *Cloudina***^109^ |
| 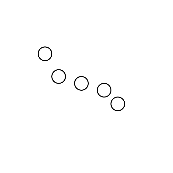 | *Neonereites* | Witputs sub-basin, Farm Vergelee (bordering Sonntagsbrunn to SE) | Nomtsas Formation | Muddy tidal | ^51^ | **UA.** Two proposed ichnospecies: *uniserialis* and *biserialis*. However, reinterpreted as either *Archaeonassa*^70,111^ or possible body fossil of unknown affinity^109^. |
| 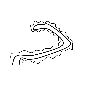 | *Nereites* | Zaris sub-basin, Farm Haruchas | Vingerbreek Member | Shallow marine | ^51^ | ***Archaeonassa*-type trace fossil**^109^ |
| 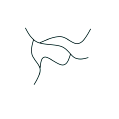 | *Olenichnus* | Witputs sub-basin, Farm Holoog | Huns Member | Sandstone-mudstone. Inner-mid ramp. | ^70^ |  |
| 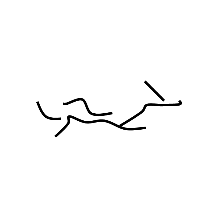 | *Palaeophycus* | Witputs sub-basin, Farm Holoog | Nasep Member | Fine- to medium-grained sandstone. Shallow marine/muddy tidal deposits. | ^112^ |  |
| 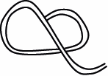 | *Parasammichnites pretzeliformis* | Witputs sub-basin, 1.2 km ESE of Koelkrans camp, Fish River Canyon | Lower Spitskop Member | Current-ripple, cross-laminated, micaceous, very fine-grained silty sandstone. Shallow subtidal dune complex. | ^54,72^ | Bilaterian bull-dozing trace. |
| 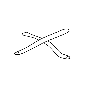 | *Planolites* | Zaris sub-basin. Witputs sub-basin, Farms Holoog, Swartpunt and Camp Koelkrans | Niederhagen Member. Nasep Member, Spitskop Member. Kreyrivier Member of Nomtsas Formation. | Sandy tidal facies. | ^38,51,54,112^ |  |
| 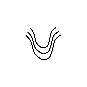 | Plug-shaped burrows | Zaris sub-basin, Farms Berghoek, Haruchas, Spider Ridge, Neuras  Witputs sub-basin, Farms Koelkrans, Kuibis, Hansburg, Canyon Roadhouse, Swartpunt | Zaris sub-basin: Niederhagen and Vingerbreek members. Witputs sub-basin: Kliphoek, Nasep and Spitskop members |  | ^54^ | Detailed description provided in (54) |
| 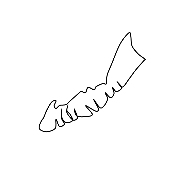 | *Scolecocoprus isp.*  *Scolicia isp.* | Witputs sub-basin, Farm Arimas, Farm Sonntagsbrunn | Nasep Quartzite Member (/lower Huns Member), Feldschuhhorn Member | Fine- to medium-grained sandstone. Shallow marine/muddy tidal deposits. | ^106^ | **UA.** Synonymous with *Muensteria* and *Taenidium*. Probable cast of *Cloudina*^109^ |
| 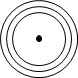 | Scratch circles | Witputs sub-basin, Farm Swartpunt and Vioolsdrif area of northwest South Africa | Spitskop Member,  Vingerbreek Member | Grey-green siltstone. Offshore to nearshore depositional setting between fair weather and storm wave base. | ^113^ |  |
| 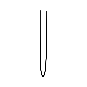 | *Skolithos* | Zaris sub-basin, Farm Grootplaas. Witputs sub-basin, Farms Geinochas, Holoog | Fish River Subgroup, Rosenhof Member. Kliphoek Member.  Urusis Formation, Nasep Member. | Coarse fluvial to tidal facies. | ^51,106,112,114^ | **UA.** Uncertain affinity in Kliphoek Member, probable body fossil^65^. *Planolites* in Nasep Member^69,109^. |
| 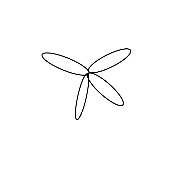 | *Streptichnus narbonnei* | Witputs sub-basin, Farm Swartpunt | Spitskop Member (medium scale seq 18 of [^73^]) | Mid-outer ramp. Occurs interbedded with outer-ramp thin-bedded limestone ~20m above fossiliferous siliciclastic transgressive systems tract. | ^70^ |  |
| 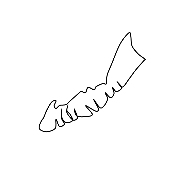 | *Taenidium* | Witputs sub-basin, Farm Arimas, Farm Sonntagsbrunn | Nasep Quartzite Member (/lower Huns Member), Feldschuhhorn Member | Fine- to medium-grained sandstone. Shallow marine/muddy tidal deposits. | ^106^ | **UA.** Synonymous with *Muensteria* and *Scolecocoprus*. Probable cast of *Cloudina*^109^ |
| 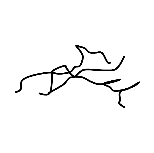 | *Torrowangea rossei* | Witputs sub-basin, Farm Holoog | Urusis Formation, Nasep Member | Fine- to medium-grained sandstone. Shallow marine/muddy tidal deposits. | ^112^ |  |
| 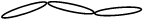 | *Treptichnus* isp*.* (Treptichnids) | Witputs sub-basin, Farms Arimas and Swartpunt, and Canyon Roadhouse | Nasep Member, Basal Huns Member, Spitskop Member | Shallow-marine, current and  wave-influenced siliciclastic  strata | ^54,69^ | Treptichnid after [70] |
| 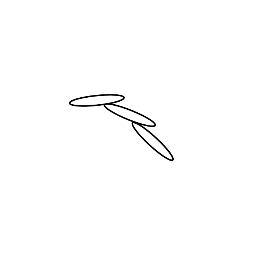 | *Treptichnus pedum* | Witputs sub-basin, Farm Sonntagsbrunn,  Farm Vergelee (bordering Sonntagsbrunn to SE) (reported in [51] as Germs pers. com.) | Nomtsas Formation (VF2 of [^71^]) | Fine- to medium-grained sandstone. Shallow marine/muddy tidal deposits. | ^71,106,112,115^ |  |
| 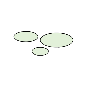 | *Beltanelliformis* | Witputs sub-basin, Farm Grens, Farm Aar | Kliphoek Member |  | ^14,60,69^ | *Nemiana* and *Hagenetta* synonymised with *Beltanelliformis*^25,79^. Cyanobacteria after molecular biomarker analyses^78^. |
| 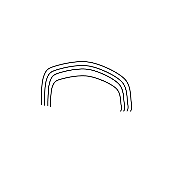  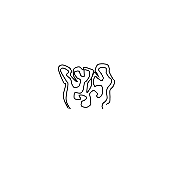 | Stromatolites and thrombolites | Zaris sub-basin, Farms Zebra River, Donkergange, and Driedoornvlakte Witputs sub-basin, Farm Swartkloofberg | Lower Spitskop Member, Huns Member, Omkyk Member, Mooifontein Member | Abundant throughout Nama succession in inner to outer ramp. Attain greatest diameter and height in deep inner ramp and mid ramp position. | ^24,34,106,116,117^ |  |
| 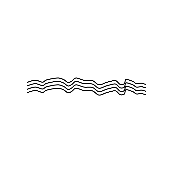 | Microbially induced sedimentary structures (MISS) | Zaris sub-basin, (D850), Farm Haruchas, Farm Bullsport. Witputs sub-basin, Farm Aar. | Niederhagen Member, Vingerbreek Member, Kliphoek Member. | Fine grained, rippled sandstone. Shallow subtidal. | ^47,48,52,60^ | e.g. Biolaminites in siliciclastic deposits, inferred microbial textures in shallow water limestones |
| 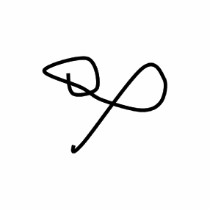 | *Vendotaenia antiqua*, tubular problematica and algal fossils | Zaris sub-basin: Farm Nutupsdrift, borehole. Witputs sub-basin: Tses Borehole and  Farms Kliphoek, Aar and Sonntagsbrunn. | Vingerbreek (Zaris and Witputs sub-basins) and Feldschuhhorn (Witputs sub-basin) members. Algal fossils: Farm Aar. | *Vendotaenia* and tubular problematica described from mid-outer ramp shales. Algal fossils described by (^118^) from clay-rich layers of the upper Aar Member include putative *Glomulus filamentum*, *Eoholinia fruticulosa* and *Tyrasotaenia podolica*. This assemblage suggests temporal equivalence with the upper Kotlin of the Russian Platform^118^. | ^31,36,71,118^ | Below FWWB in the Nama succession. |
| 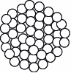 | Possible cyanobacteria, including *Bavlinella faveolata* | Zaris sub-basin, Nutupsdrift borehole. Witputs sub-basin, Tses borehole | Urusis Formation (Witputs). Vingerbreek Member (both sub-basins). Kuibis Subgroup (Witputs) |  | ^31^ | Possible cyanobacterial sheaths, *Chuaria circularis* and *Bavlinella faveolata*^119^ |

| **Body Fossils** | | | | | | |
| --- | --- | --- | --- | --- | --- | --- |
| **Soft-bodied** | | | | | | |
| **Symbol** | **ID (red: omitted from compilation)** | **Notable occurrences** | **Stratigraphic Position** | **Sedimentological context** | **References** | **Affinity/Notes** |
| 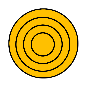 | *Aspidella*, *Paramedusium*, *Cyclomedusa* | Zaris sub-basin, Road transect (D850 and D855). Witputs sub-basin, Farms Groendorn (Grondorner) and Swartpunt | Vingerbreek Member.  Spitskop Member. Huns Member. (?)Nasep Member. Kliphoek Member. | Vingerbreek Member: fine-grained, symmetrical rippled sandstone above fair weather wave base. Spitskop Member: float in laminated siltstone/mudstone facies. | ^47,68,69,106,114^ | Junior synonyms of *Aspidella* after (^120^) |
| 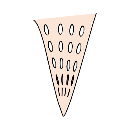 | *Ausia fenestrata* | Witputs sub-basin, Farm Plateau. | Kliphoek Member | Coarse quartz-rich sandstone. | ^114,121^ | Possible tunicate? |
| 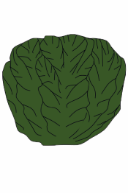 | *Bradgatia* | Witpus sub-basin, Farm Swartpunt | Upper Spitskop Member. | Coarse sandstone facies | ^68^ | **UA.** Putative occurrence. Uncertain affinity. Provisional assignment. |
| 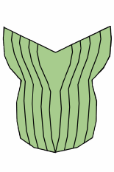 | *Ernietta* | Witputs sub-basin, Farm Plateau, Farm Aar, Farm Wegkruip, Farm Hansburg  Farm Swartpunt. | Upper Kliphoek (Aar) Member (reassignment of Farm Hansburg samples to Kliphoek Member after [^19^]),  Spitskop Member | Spitskop Member: Float in fine sandstone. Kliphoek Member: quartz arenite with syneresis cracks, injection structures, current ripples and hummocky cross-stratification. Transgressive grey-green shale with siltstone and fine sandstone event beds. Interpreted as muddy mid-ramp below fair weather wave base. | ^19,20,56,61,68,122,123^ | Provisional assignment to *Ernietta* for one partial specimen recovered at Swartpunt after (^68^). This specimen is considered of uncertain affinity **(UA)** for the purpose of the summarised taxonomic diversity herein. |
| 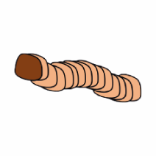 | *Gaojiashania* | Zaris sub-basin, Farm Donkergange. | Basal Hoogland Member. | Fine micaceous sandstone. Transgressive systems tract. | ^63^ | Known from one specimen. Possible palaeopasichnid. |
| 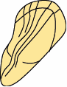 | *Kuibisia glabra* | Witputs sub-basin, Farm Plateau | Dabis Formation (Kliphoek Member?) | Kuibis quartzite. Slightly micaceous quartzite. | ^121^ | **UA**. Possibly *Ernietta*^124^ or external surface of *Ausia*^125^ |
| 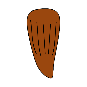 | *Namalia villiersiensis* | Witputs sub-basin, multiple unspecified localities from Aus-Buchholzbrunn to Helmeringhausen | Kliphoek Member | Kuibis quartzite. Orthoquartzite | ^57,92^ |  |
| 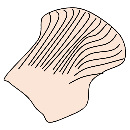 | *Nasepia altae* | Witputs sub-basin, Farm Swartpunt, Farm Arimas. | Spitskop Member, Nasep Member. | Spitskop Member specimen in medium-coarse sandstone. | ^37,68^ | *Nasepia* in Spitskop Member represents the provisional assignment of a partial specimen^68^. This specimen is considered of uncertain affinity **(UA)** for the purpose of the summarised taxonomic diversity herein. |
| 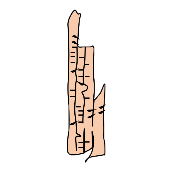 | *Orthogonium parallelum* | Witputs sub-basin, Farm Kuibis | Kliphoek Member | Quartz-rich sandstone(?) | ^38,55,114,126,127^ | **UA.** Only specimen lost in WW2 |
| 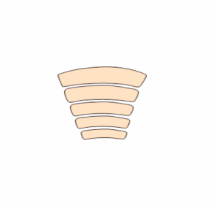 | *Palaeopasichnus* | Witputs sub-basin.  Zaris sub-basin, Farm Omkyk. | Kliphoek Member. Base of Upper Omkyk Member. | Weathering-resistant quartzite. | Witputs sub-basin:  unspecified locality^69^. Zaris sub-basin: Farm Omkyk (own observation). | *Palaeopasichnus* at base of OS2, Farm Omkyk. Occurs at top of ~20cm thick quartzite bed. Carbonate δ^13^C isotope and sequence stratigraphy supports equivalence with (/slightly younger than) Aar Member of Witputs sub-basin. Probable protozoan body fossil after (^128^) |
| 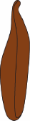 | *Protechiurus edmondsi* | Witputs sub-basin, Farm Plateau | Dabis Formation, Kliphoek Member? | Kuibis quartzite. Slightly micaceous quartzite. | ^129^ | **UA.** Dubiofossil^124^(?) or proto-chordate^114,125^(?) |
| 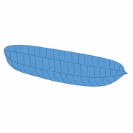 | *Pteridinium* | Witputs sub-basin, Farm Swartpunt, 25km north of Farm Helmeringhausen (Kosos?), Farm Aar. | Spitskop Member, Vingerbreek Member, Niederhagen Member, Upper Kliphoek (Aar) Member. | Spitskop Member: Float and some *in situ* specimens in medium-coarse sandstone. Kliphoek Member: quartzarenite containing syneresis cracks, injection structures, current ripples and hummocky cross-stratification. Transgressive grey-green shale with siltstone and fine sandstone event beds. Interpreted as muddy mid-ramp below fair weather wave base. | ^25,38,55,61,68,92,104,126,127,130–132^ |  |
| 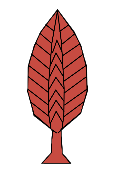 | *Rangea* | Witputs sub-basin, Farms Kuibis, Vrede, Aar and Chamis. | Niederhagen Member, Kliphoek Member. | Kliphoek Member: quartzarenite containing syneresis cracks, injection structures, current ripples and hummocky cross-stratification. Transgressive grey-green shale with siltstone and fine sandstone event beds. Interpreted as muddy mid-ramp below fair weather wave base. Fossils occur within hummocky cross-stratified, fine-grained quartz sandstone caps of gutter casts on Farm Aar. | ^25,37,55,59–61,126^ | Possible ctenophore^87^ |
| 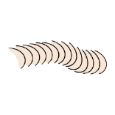 | *Shaanxilithes* | Zaris sub-basin, Road transect (D850). Farm Neuras. | Vingerbreek Member. | Siltstones and fine-grained sandstones | ^47^ | Possible misidentification of *Palaeopasichnus* |
| 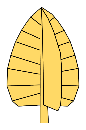 | *Swartpuntia* | Witputs sub-basin, Farm Swartpunt. | Spitskop Member, Feldschuhhorn Member. | Spitskop Member: Float in medium and coarse (cross-bedded) sandstone, and fine sandstone with ripple cross-laminations. One specimen recorded potentially *in situ*. | ^68,69,130^ |  |
| 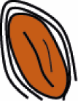 | *Velancorina martina* | Witputs sub-basin, Farms Aar and Schakalskuppe | Kuibis Formation (Kliphoek Member) | Quartz-rich sandstone(?) | ^56,125^ | **UA.** Phylum Petalonamae |

| **Skeletal** | | | | | | |
| --- | --- | --- | --- | --- | --- | --- |
| **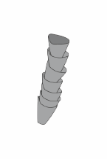** | *Cloudina* | Zaris sub-basin, Farms Zwartmodder, Omkyk, Zebra River, Donkergange, Driedoornvlakte. Witputs sub-basin, Farm Swartpunt, Farm Mooifontein, Farm Uitsig, Vioolsdrif region (South Africa) | Spitskop Member, Huns Member, Hoogland Member, Upper Omkyk Member, Mooifontein Member, Mara Member | Inner and mid ramp limestones throughout Nama succession. Thin-bedded outer ramp limestones at Farm Swartpunt (Spitskop Member). | ^8,22,25,27,38,117,133^ | Two possible species in Namibia: *hartmannae* and *riemkeae* but based on growth mode not size distribution^22,133^. Probable total group cnidarian. |
| **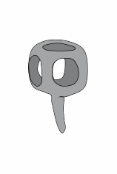** | *Namacalathus* | Zaris sub-basin, Farms Zwartmodder, Omkyk, Zebra River, Driedoornvlakte. Witputs sub-basin, Farms Swartpunt, Swartkloofberg and Grens | Spitskop Member, Feldschuhhorn Member, Upper Omkyk Member and Kliphoek Member | Inner to mid-ramp facies in Kuibis Subgroup. Mid-ramp facies in pinnacle reef locality (Feldschuhhorn Member flooding surface and TST atop Huns Member). Outer ramp, thinly bedded limestone in upper Spitskop Member HST (at summit of Swartpunt section – own observation). | ^14,86,115,134^ | Putative lophophorate^135^ |
| **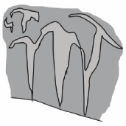** | *Namapoikia* | Zaris sub-basin, Farm Driedoornvlakte | Upper Omkyk Member (OS2) | Only documented occurrence from mid-ramp carbonate platform on Farm Driedoornvlakte. Occupies syn-sedimentary fissures (neptunian dikes) that formed during transgression of Upper Omkyk Member, Unit 3m. | ^62,136^ | Probable poriferan^136^ |

**References**

1. Poulton, S. & Canfield, D. Development of a sequential extraction procedure for iron: implications for iron partitioning in continentally derived particulates. *Chem. Geol.* **214**, 209–221 (2005).

2. Canfield, D. E., Raiswell, R., Westrich, J. T., Reaves, C. M. & Berner, R. a. The use of chromium reduction in the analysis of reduced inorganic sulfur in sediments and shales. *Chem. Geol.* **54**, 149–155 (1986).

3. Ruttenberg, K. C. Development of a sequential extraction method for different forms of phosphorus in marine sediments. *Limnol. Oceanogr.* **37**, 1460–1482 (1992).

4. Thompson, J. *et al.* Development of a modified SEDEX phosphorus speciation method for ancient rocks and modern iron-rich sediments. *Chem. Geol.* **524**, 383–393 (2019).

5. März, C., Poulton, S. W., Wagner, T., Schnetger, B. & Brumsack, H. J. Phosphorus burial and diagenesis in the central Bering Sea (Bowers Ridge, IODP Site U1341): Perspectives on the marine P cycle. *Chem. Geol.* **363**, 270–282 (2014).

6. Ruttenberg, K. & Berner, R. Authigenic apatite formation and burial in sediments from non-upwelling, continental margin environments. *Geochim. Cosmochim. Acta* **57**, 991–1007 (1993).

7. Slomp, C. P. *et al.* Coupled Dynamics of Iron and Phosphorus in Sediments of an Oligotrophic Coastal Basin and the Impact of Anaerobic Oxidation of Methane. *PLoS One* **8**, (2013).

8. Germs, G. J. B. Implications of a sedimentary facies and depositional environmental analysis of the Nama Group in South West Africa/Namibia. *Spec. Publ. Geol. Soc. South Africa* **11**, 89–114 (1983).

9. Gresse, P. G. & Germs, G. J. B. The Nama foreland basin: sedimentation, major unconformity bounded sequences and multisided active margin advance. *Precambrian Res.* **63**, (1993).

10. Saylor, B., Grotzinger, J. P. & Germs, G. Sequence stratigraphy and sedimentology of the Neoproterozoic Kuibis and Schwarzrand Subgroups (Nama Group), southwestern Namibia. *Precambrian Res.* **73**, 153–171 (1995).

11. Saylor, B. Z., Kaufman, A. J., Grotzinger, J. P. & Urban, F. A composite reference section for terminal proterozoic strata of southern Namibia. *J. Sediment. Res.* **68**, 1223–1235 (1998).

12. Kaufman, A. J., Hayes, J. M., Knoll, A. H. & Germs, G. J. B. Isotopic compositions of carbonates and organic carbon from upper Proterozoic successions in Namibia: stratigraphic variation and the effects of diagenesis and metamorphism. *Precambrian Res.* **49**, 301–327 (1991).

13. Smith, O. A. Terminal Proterozoic Carbonate Platform Development: Stratigraphy and Sedimentology of the Kuibis Subgroup (ca. 550-548 Ma), Northern Nama Basin, Namibia. (Massachusetts Institute of Technology, 1998).

14. Wood, R. A. *et al.* Dynamic redox conditions control late Ediacaran metazoan ecosystems in the Nama Group, Namibia. *Precambrian Res.* **261**, 252–271 (2015).

15. Halverson, G. P., Hoffman, P. F., Schrag, D. P., Maloof, A. C. & Rice, a. H. N. Toward a Neoproterozoic composite carbon-isotope record. *Geol. Soc. Am. Bull.* **117**, 1181 (2005).

16. Zhu, M. Y., Babcock, L. E. & Peng, S. C. Advances in Cambrian stratigraphy and paleontology: Integrating correlation techniques, paleobiology, taphonomy and paleoenvironmental reconstruction. *Palaeoworld* **15**, 217–222 (2006).

17. Broecker, W. S. & Peng, T.-H. Tracers in the sea. *Geochim. Cosmochim. Acta* **47**, 1336 (1982).

18. Sperling, E. A. *et al.* Statistical analysis of iron geochemical data suggests limited late Proterozoic oxygenation. *Nature* **523**, 451–454 (2015).

19. Gibson, B. M. *et al.* Gregarious suspension feeding in a modular Ediacaran organism. *Sci. Adv.* **5**, 1–10 (2019).

20. Bouougri, E. H., Porada, H., Weber, K. & Reitner, J. Sedimentology and Palaeoecology of Ernietta-Bearing Ediacaran Deposits in Southern Namibia: Implications for Infaunal Vendobiont Communities. in *Advances in stromatolite geobiology* (eds. Reitner, J., Quéric, N.-V. & Arp, G.) **131**, 473–506 (Springer Berlin Heidelberg, 2011).

21. Macdonald, F. A., Pruss, S. B. & Strauss, J. V. Trace Fossils with Spreiten from the Late Ediacaran Nama Group, Namibia: Complex Feeding Patterns Five Million Years Before the Precambrian–Cambrian Boundary. *J. Paleontol.* **88**, 299–308 (2014).

22. Germs, G. J. B. New shelly fossils from Nama Group, South West Africa. *American Journal of Science* **272**, 752–761 (1972).

23. Grotzinger, J. P. Facies and paleoenvironmental setting of Thrombolite-Stromatolite Reefs , Terminal Proterozoic Nama Group ( ca . 550-543 Ma ), central and southern Namibia. *Commun. Geol. Surv. Namib* **12**, 251–264 (2000).

24. Adams, E. W., Schroder, S., Grotzinger, J. P., McCormick, D. S. & Anonymous. Digital reconstruction and stratigraphic evolution of a microbial-dominated, isolated carbonate platform (terminal Proterozoic, Nama Group, Namibia). *J. Sediment. Res.* **74**, 479–497 (2004).

25. Grant, S. W. F. Shell structure and distribution of Cloudina, a potential index fossil for the terminal Proterozoic. *American Journal of Science* **290 A**, 261–294 (1990).

26. Penny, A. M. *et al.* Ediacaran metazoan reefs from the Nama Group, Namibia. *Science (80-. ).* **344**, 1504–1506 (2014).

27. Wood, R. & Curtis, A. Extensive metazoan reefs from the Ediacaran Nama Group, Namibia: The rise of benthic suspension feeding. *Geobiology* **13**, 112–122 (2015).

28. Tostevin, R. *et al.* Low-oxygen waters limited habitable space for early animals. *Nat. Commun.* **7**, 12818 (2016).

29. Bowyer, F., Wood, R. A. & Poulton, S. W. Controls on the evolution of Ediacaran metazoan ecosystems: A redox perspective. *Geobiology* **15**, 516–551 (2017).

30. Turekian, K. K. & Wedepohl, K. H. Distribution of the Elements in Some Major Units of the Earth’s Crust. *Geol. Soc. Am. Bull.* **72**, 175–192 (1961).

31. Germs, G. J. B., Knoll, A. H. & Vidal, G. Latest proterozoic microfossils from the Nama group, Namibia (south west Africa). *Precambrian Res.* **32**, 45–62 (1986).

32. Lyons, T. W. & Severmann, S. A critical look at iron paleoredox proxies: New insights from modern euxinic marine basins. *Geochim. Cosmochim. Acta* **70**, 5698–5722 (2006).

33. Raiswell, R. *et al.* Turbidite depositional influences on the diagenesis of Beecher’s Trilobite Bed and the Hunsrück Slate; sites of soft tissue pyritization. *Am. J. Sci.* **308**, 105–129 (2008).

34. Adams, E. W. *et al.* Digital characterization of thrombolite-stromatolite reef distribution in a carbonate ramp system (terminal Proterozoic, Nama Group, Namibia). *Am. Assoc. Pet. Geol. Bull.* **89**, 1293–1318 (2005).

35. Wood, R., Bowyer, F., Penny, A. & Poulton, S. W. Did anoxia terminate Ediacaran benthic communities? Evidence from early diagenesis. *Precambrian Res.* **313**, 134–147 (2018).

36. Cohen, P. A. *et al.* Tubular Compression Fossils from the Ediacaran Nama Group, Namibia. *J. Paleontol.* **83**, 110–122 (2009).

37. Germs, G. J. B. A reinterpretation of Rangea schneiderhoehni and the discovery of a related new fossil from the Nama Group, South West Africa. *Lethaia* **6**, 1–9 (1973).

38. Germs, G. J. B. The Neoproterozoic of southwestern Africa, with emphasis on platform stratigraphy and paleontology. *Precambrian Res.* **73**, 137–151 (1995).

39. Grotzinger, J. P. & Miller, R. M. The Nama Group. in *The Geology of Namibia, Vol. 2* (ed. Miller, R. M.) 13.229-13.272 (Geological Survey of Namibia, 2008).

40. Saylor, B. Z. Sequence stratigraphic and chemostratigraphic constraints on the evolution of the terminal Proterozoic to Cambrian Nama Basin, Namibia. (Massachusetts Institute of Technology, 1996).

41. Grotzinger, J. P. Stratigaphy, facies, and paleoenvironmental setting of a terminal Proterozoic carbonate ramp, Nama Group (550-543 Ma), Namibia. in *Johannesburg, South Africa, 16th International Sedimentological Congress, Field Guide* 71 (2002).

42. Poulton, S. W. & Raiswell, R. The low-temperature geochemical cycle of iron: From continental fluxes to marine sediment deposition. *Am. J. Sci.* **302**, 774–805 (2002).

43. Cumming, V. M., Poulton, S. W., Rooney, A. D. & Selby, D. Anoxia in the terrestrial environment during the late Mesoproterozoic. *Geology* **41**, 583–586 (2013).

44. Saylor, B. Z., Poling, J. M. & Huff, W. D. Stratigraphic and chemical correlation of volcanic ash beds in the terminal Proterozoic Nama Group, Namibia. *Geol. Mag.* **142**, 519 (2005).

45. Lechte, M. A. & Wallace, M. W. Sedimentary and tectonic history of the Holowilena Ironstone, a Neoproterozoic iron formation in South Australia. *Sediment. Geol.* **329**, 211–224 (2015).

46. Germs, G. J. B. & Gaucher, C. Nature and Extent of a Late Ediacaran (Ca. 547 Ma) Glacigenic Erosion Surface in Southern Africa. *South African J. Geol.* **115**, 91–102 (2012).

47. Darroch, S. A. F. *et al.* A mixed Ediacaran-metazoan assemblage from the Zaris Sub-basin, Namibia. *Palaeogeogr. Palaeoclimatol. Palaeoecol.* **459**, 198–208 (2016).

48. Noffke, N., Knoll, A. H. & Grotzinger, J. P. Sedimentary Controls on the Formation and Preservation of Microbial Mats in Siliciclastic Deposits: A Case Study from the Upper Neoproterozoic Nama Group, Namibia. *Palaios* **17**, 533–544 (2002).

49. Germs, G. J. B. & Gresse, P. . The foreland basin of the Damara and Gariep orogens in Namaqualand and southern Namibia: stratigraphic correlations and basin dynamics. *South African J. Geol.* **94**, 159–169 (1991).

50. Dibenedetto, S. & Grotzinger, J. P. Geomorphic evolution of a storm-dominated carbonate ramp ( c. 549 Ma), Nama Group, Namibia. *Geol. Mag.* **142**, 583 (2005).

51. Crimes, T. P. & Germs, G. J. B. Trace Fossils from the Nama Group (Precambrian-Cambrian) of Southwest Africa (Namibia). *J. Paleontol.* **56**, 890–907 (1982).

52. Bouougri, E. H. & Porada, H. Siliciclastic biolaminites indicative of widespread microbial mats in the Neoproterozoic Nama Group of Namibia. *J. African Earth Sci.* **48**, 38–48 (2007).

53. Raiswell, R. O. B. *et al.* The Iron paleoredox proxies: A guide to the pitfalls, problems and proper practice. *Am. J. Sci.* **318**, 491–526 (2018).

54. Cribb, A. T. *et al.* Increase in metazoan ecosystem engineering prior to the Ediacaran–Cambrian boundary in the Nama Group, Namibia. *R. Soc. Open Sci.* **6**, 190548 (2019).

55. Gurich, G. Uber den Kuibisquartzit in Sudwest-afrika. *Zeitschrift Dtsch. Geol. Gesellschaft* **82**, 637 (1930).

56. Pflug, H. D. Neue fossilreste aus den NamaSchichten in Sudwest-Afrika. *Paläontologische Zeitschrift* **40**, 14–25 (1966).

57. Germs, G. J. B. Discovery of a New Fossil in the Nama System, South West Africa. *Nature* **219**, 53–54 (1968).

58. Germs, G. J. B. Possible Sprigginid Worm and a New Trace Fossil from the Nama Group, South West Africa. *Geology* **1**, 69 (1973).

59. Jenkins, R. J. F. The Enigmatic Ediacaran (Late Precambrian) Genus Rangea and Related Forms. *Paleobiology* **11**, 336–355 (1985).

60. Vickers-Rich, P. *et al.* Reconstructing Rangea: new discoveries from the Ediacaran of southern Namibia. *J. Paleontol.* **87**, 1–15 (2013).

61. Hall, M. *et al.* Stratigraphy, palaeontology and geochemistry of the late Neoproterozoic Aar Member, southwest Namibia: Reflecting environmental controls on Ediacara fossil preservation during the terminal Proterozoic in African Gondwana. *Precambrian Res.* **238**, 214–232 (2013).

62. Wood, R. A., Grotzinger, J. P. & Dickson, J. A. D. Proterozoic Modular Biomineralized Metazoan from the Nama Group, Namibia. *Science (80-. ).* **296**, 2383–2386 (2002).

63. Smith, E. F., Nelson, L. L., Tweedt, S. M., Zeng, H. & Workman, J. B. A cosmopolitan late ediacaran biotic assemblage: New fossils from Nevada and Namibia support a global biostratigraphic link. *Proc. R. Soc. B Biol. Sci.* **284**, 20170934 (2017).

64. Buatois, L. A. & Mángano, M. G. Ediacaran Ecosystems and the Dawn of Animals. in *The Trace-Fossil Record of Major Evolutionary Events* (eds. Mángano, M. G. & Buatois, L. A.) **40**, 27–72 (Springer, 2016).

65. Crimes, T. P. & Fedonkin, M. A. Biotic changes in platform communities across the precambrian Phanerozoic boundary. *Riv. Ital. di Paleontol. e Stratigr.* **102**, 317–332 (1996).

66. Aceñolaza, G. F., Germs, G. J. B. & Aceñolaza, F. G. Trace Fossils and the Agronomic Revolution at the Neoproterozoic-Cambrian Transition in Southwest Gondwana. in *Neoproterozoic-Cambrian Tectonics, Global Change and Evolution: a focus on southwestern Gondwana. Developments in Precambrian Geology* (eds. Gaucher, C., Sial, A. N., Halverson, G. P. & Frimmel, H. E.) **16**, 339–347 (Elsevier, 2009).

67. Narbonne, G. M., Saylor, B. Z. & Grotzinger, J. P. The youngest Ediacaran fossils from southern Africa. *J. Paleontol.* **71**, 953–967 (1997).

68. Darroch, S. A. F. *et al.* Biotic replacement and mass extinction of the Ediacara biota. *Proc. R. Soc. B Biol. Sci.* **282**, 20151003 (2015).

69. Jensen, S., Saylor, B. Z., Gehling, J. G. & Germs, G. J. B. Complex trace fossils from terminal Proterozoic of Namibia. *Geology* **28**, 143–146 (2000).

70. Jensen, S. M. & Runnegar, B. N. A complex trace fossil from the Spitskop Member (terminal Ediacaran–? Lower Cambrian) of southern Namibia. *Geol. Mag.* **142**, 561–569 (2005).

71. Wilson, J. P. *et al.* Deep-Water Incised Valley Deposits At the Ediacaran-Cambrian Boundary in Southern Namibia Contain Abundant Treptichnus Pedum. *Palaios* **27**, 252–273 (2012).

72. Buatois, L. A., Almond, J., Mángano, M. G., Jensen, S. & Germs, G. J. B. Sediment disturbance by Ediacaran bulldozers and the roots of the Cambrian explosion. *Sci. Rep.* **8**, 1–9 (2018).

73. Saylor, B. Z. Sequence stratigraphy and carbonate-siliciclastic mixing in a terminal Proterozoic foreland basin, Urusis Formation, Nama Group, Namibia. *J. Sediment. Res.* **73**, 264–279 (2003).

74. Saylor, B. Z. & Grotzinger, J. P. Reconstruction of important Proterozoic-Cambrian boundary exposures through the recognition of thrust deformation in the Nama Group of southern Namibia. *Commun. - Geol. Surv. Namibia* **11**, 1–12 (1996).

75. Xiao, S. & Laflamme, M. On the eve of animal radiation: phylogeny, ecology and evolution of the Ediacara biota. *Trends Ecol. Evol.* **24**, 31–40 (2009).

76. Droser, M. L. & Gehling, J. G. The advent of animals : The view from the Ediacaran. *Proc. Natl. Acad. Sci.* **112**, 4865–4870 (2015).

77. Muscente, A. D., Boag, T. H., Bykova, N. & Schiffbauer, J. D. Environmental disturbance, resource availability, and biologic turnover at the dawn of animal life. *Earth-Science Rev.* **177**, 248–264 (2017).

78. Bobrovskiy, I., Hope, J. M., Krasnova, A., Ivantsov, A. & Brocks, J. J. Molecular fossils from organically preserved Ediacara biota reveal cyanobacterial origin for Beltanelliformis. *Nat. Ecol. Evol.* **2**, 437–440 (2018).

79. Ivantsov, A. Y., Gritsenko, V. P., Konstantinenko, L. I. & Zakrevskaya, M. A. Revision of the problematic Vendian macrofossil Beltanelliformis (=Beltanelloides, Nemiana). *Paleontol. J.* **48**, 1415–1440 (2014).

80. Fedonkin, M. A. & Waggoner, B. M. The Late Precambrian fossil Kimberella is a mollusc-like bilaterian organism. *Nature* **388**, 868–871 (1997).

81. Ivantsov, A. Y. New reconstruction of Kimberella, problematic Vendian metazoan. *Paleontol. J.* **43**, 601–611 (2009).

82. Evans, S. D., Droser, M. L. & Gehling, J. G. Highly regulated growth and development of the Ediacara macrofossil Dickinsonia costata. *PLoS One* **12**, 1–15 (2017).

83. Hoekzema, R. S., Brasier, M. D., Dunn, F. S. & Liu, A. G. Quantitative study of developmental biology confirms Dickinsonia as a metazoan. *Proc. R. Soc. B Biol. Sci.* **284**, 20171348 (2017).

84. Sperling, E. A. *et al.* Oxygen, ecology, and the Cambrian radiation of animals. *Proc. Natl. Acad. Sci.* **110**, 13446–13451 (2013).

85. Sperling, E. A., Knoll, A. H. & Girguis, P. R. The Ecological Physiology of Earth’s Second Oxygen Revolution. *Annu. Rev. Ecol. Evol. Syst.* **46**, 215–235 (2015).

86. Penny, A. M. *et al.* Intraspecific variation in an Ediacaran skeletal metazoan : Namacalathus from the Nama Group , Namibia. *Geobiology* **15**, 81–93 (2017).

87. Dzik, J. Possible ctenophoran affinities of the Precambrian ‘sea-pen’ Rangea. *J. Morphol.* **252**, 315–334 (2002).

88. Weber, B., Steiner, M. & Zhu, M. Y. Precambrian-Cambrian trace fossils from the Yangtze Platform (South China) and the early evolution of bilaterian lifestyles. *Palaeogeogr. Palaeoclimatol. Palaeoecol.* **254**, 328–349 (2007).

89. Fedonkin, M. A., Vickers-Rich, P., Swalla, B. J., Trusler, P. & Hall, M. A new metazoan from the Vendian of the White Sea, Russia, with possible affinities to the ascidians. *Paleontol. J.* **46**, 1–11 (2012).

90. Meyer, M. *et al.* Three-dimensional microCT analysis of the Ediacara fossil Pteridinium simplex sheds new light on its ecology and phylogenetic affinity. *Precambrian Res.* **249**, 79–87 (2014).

91. Chang, A. J., Chronis, N., Karow, D. S., Marletta, M. A. & Bargmann, C. I. A Distributed Chemosensory Circuit for Oxygen Preference in C. elegans. *PLoS Biol.* **4**, e274 (2006).

92. Grazhdankin, D. & Seilacher, A. Underground Vendobionta from Namibia. *Palaeontology* **45**, 57–78 (2002).

93. Bottjer, D. J., Hagadorn, J. W. & Dornbos, S. Q. The Cambrian Substrate Revolution. *GSA Today* **10**, 1–7 (2000).

94. Tarhan, L. G. The early Paleozoic development of bioturbation—Evolutionary and geobiological consequences. *Earth-Science Rev.* **178**, 177–207 (2018).

95. Droser, M. L. & Bottjer, D. J. A Semiquantitative Field Classification of Ichnofabric. *J. Sediment. Res.* **56**, 5–6 (1986).

96. Miller, M. F. & Smail, S. E. A Semiquantitative Field Method for Evaluating Bioturbation on Bedding Planes. *Palaios* **12**, 391–396 (1997).

97. Nesbitt, H. W. & Young, G. M. Early Proterozoic climates and plate motions inferred from major element chemistry of lutites. *Nature* **299**, 715–717 (1982).

98. Fedo, C. M., Nesbitt, H. W. & Young, G. M. Unraveling the effects of potassium metasomatism in sedimentary rocks and paleosols, with implications for paleoweathering conditions and provenance. *Geology* **23**, 921–924 (1995).

99. Zhai, L., Wu, C., Ye, Y. & Zhang, S. Fluctuations in chemical weathering on the Yangtze Block during the Ediacaran-Cambrian transition: implications for paleoclimatic conditions and the marine carbon cycle. *Palaeogeogr. Palaeoclimatol. Palaeoecol.* **490**, 280–292 (2018).

100. McLennan, S. M. Weathering and Global Denudation. *J. Geol.* **101**, 295–303 (1993).

101. Tosca, N. J. *et al.* Clay mineralogy, organic carbon burial, and redox evolution in Proterozoic oceans. *Geochim. Cosmochim. Acta* **74**, 1579–1592 (2010).

102. Johnston, D. T. *et al.* Searching for an oxygenation event in the fossiliferous Ediacaran of northwestern Canada. *Chem. Geol.* **362**, 273–286 (2013).

103. Blanco, G. *et al.* Provenance and paleogeography of the Nama Group (Ediacaran to early Palaeozoic, Namibia): Petrography, geochemistry and U-Pb detrital zircon geochronology. *Precambrian Res.* **187**, 15–32 (2011).

104. Glaessner, M. F. Zur Kenntnis der Nama-Fossilien Südwest-Africa. *Ann. Naturhistor. Mus. Wien.* **66**, 113–120 (1963).

105. Glaessner, M. F. Re-examination of Archaeichnium, a fossil from the Nama Group. *Ann. he South African Museum* **74**, 335–342 (1978).

106. Germs, G. J. B. Trace Fossils from the Nama Group, South-West Africa. *J. Paleontol.* **46**, 864–870 (1972).

107. Crimes, T. P. Trace Fossils. in *The Precambrian-Cambrian Boundary* (eds. Cowie, J. W. & Brasier, M. D.) 166–185 (Clarendon Press, Oxford, 1989).

108. Menon, L. R., McIlroy, D. & Brasier, M. D. ‘ Intrites ’ from the Ediacaran Longmyndian Supergroup, UK: a new form of microbially-induced sedimentary structure (MISS) . *Geol. Soc. London, Spec. Publ.* **448**, 271–283 (2016).

109. Jensen, S., Droser, M. L. & Gehling, J. G. A Critical Look at the Ediacaran Trace Fossil Record. in *Neoproterozoic geobiology and paleobiology* (eds. Kaufman, A. J. & Xiao, S.) 115–157 (Springer, 2006).

110. Ciampaglio, C. N., Babcock, L. E., Wellman, C. L., York, A. R. & Brunswick, H. K. Phylogenetic affinities and taphonomy of Brooksella from the Cambrian of Georgia and Alabama, USA. *Palaeoworld* **15**, 256–265 (2006).

111. Jensen, S. The Proterozoic and Earliest Cambrian Trace Fossil Record; Patterns, Problems and Perspectives. *Integr. Comp. Biol.* **43**, 219–228 (2003).

112. Geyer, G. & Uchman, A. Ichnofossil assemblages from the Nama Group (Neoproterozoic-Lower Cambrian) in Namibia and the Proterozoic-Cambrian boundary problem revisited. *Beringeria Spec. Issue* **2**, 175–202 (1995).

113. Jensen, S. *et al.* Scratch circles from the Ediacaran and Cambrian of Arctic Norway and Southern Africa, with a review of scratch circle occurrences. *Bull. Geosci.* **93**, 287–304 (2018).

114. Pickford, M. H. L. Review of the Riphean , Vendian and early Cambrian palaeontology of the Otavi and Nama Groups, Namibia. *Commun. Geol. Surv. Namibia* **10**, 57–81 (1995).

115. Grotzinger, J. P., Bowring, S. A., Saylor, B. Z. & Kaufman, A. J. Biostratigraphic and Geochronologic Constraints on Early Animal Evolution. *Science (80-. ).* **270**, 598–604 (1995).

116. Grotzinger, J. P., Watters, W. A. & Knoll, A. H. Calcified metazoans in thrombolite-stromatolite reefs of the terminal Proterozoic Nama Group, Namibia. *Paleobiology* **26**, 334–359 (2000).

117. Penny, A. . *et al.* Ediacaran metazoan reefs from the Nama Group, Namibia. *Science (80-. ).* **344**, 1504–1506 (2014).

118. Leonov, M. V. & Fedonkin, M. A. Discovery of the first macroscopic algal assemblage in the Terminal Proterozoic of Namibia , southwest Africa. *Commun. Geol. Surv. Namib* **14**, 87–93 (2009).

119. Mansuy, C. & Vidal, G. Late Proterozoic Brioverian microfossils from France: taxonomic affinity and implications of plankton productivity. *Nature* **302**, 606–607 (1983).

120. Gehling, J. G. The first named Ediacaran body fossil Aspidella terranovica. *Palaeontology* **43**, 427–456 (2000).

121. Hahn, G. & Pflug, H. Polypenartige Organismen aus dem Jung-Prakambrium (Nama-Gruppe) von Namibia. *Geol. Palaeontol.* **19**, 1–13 (1985).

122. Dzik, J. Organic membranous skeleton of the Precambrian metazoans from Namibia. *Geology* **27**, 519–522 (1999).

123. Elliott, D. A. *et al.* Ernietta from the late Edicaran Nama Group, Namibia. *J. Paleontol.* **90**, 1017–1026 (2016).

124. Runnegar, B. N. Proterozoic fossils of soft-bodied metazoans (Ediacaran faunas). in *The Proterozoic Biosphere: A multidisciplinary study* (eds. Schopf, J. W. & Klein, C.) 999–1007 (Cambridge University Press, 1992).

125. Fedonkin, M. A., Gehling, J. G., Grey, K., Narbonne, G. M. & Vickers-Rich, P. *The rise of animals: evolution and diversification of the kingdom Animalia*. (Johns Hopkins University Press, 2007).

126. Gurich, G. Die bislang altesten Spuren von Organismen in Sudafrika. *Int. Geol. Congr. S. Africa* **15**, 670–680 (1930).

127. Gurich, G. Die Kuibis-Fossillien der Nama-Formation von Sudwest-Afrika. *Palaontologische Zeitschrift* **15**, 137–154 (1933).

128. Antcliffe, J. B., Gooday, A. J. & Brasier, M. D. Testing the protozoan hypothesis for Ediacaran fossils: A developmental analysis of Palaeopascichnus. *Palaeontology* **54**, 1157–1175 (2011).

129. Glaessner, M. F. An echiurid worm from the Late Precambrian. *Lethaia* **12**, 121–124 (1979).

130. Narbonne, G. M., Saylor, B. Z. & Grotzinger, J. P. The youngest Ediacaran fossils from southern Africa. *J. Paleontol.* **71**, 953–967 (1997).

131. Elliott, D. A., Vickers-Rich, P., Trusler, P. & Hall, M. New evidence on the taphonomic context of the Ediacaran Pteridinium. *Acta Palaeontol. Pol.* **56**, 641–650 (2011).

132. Pflug, H. D. Zur fauna der Nama-Schichten in Sudwest-Afrika; I. Pteridinia, bau und systematische zugehorigkeit. *Palaeontogr. Abteilung* **134**, 226–262 (1970).

133. Wood, R. *et al.* Flexible and responsive growth strategy of the Ediacaran skeletal Cloudina from the Nama Group, Namibia. *Geology* **45**, 259–262 (2017).

134. Watters, W. A. Digital Reconstructions of Fossil Morphologies, Nama Group, Namibia. (Massachusetts Institute of Technology, 2000).

135. Zhuravlev, A. Y., Wood, R. A. & Penny, A. M. Ediacaran skeletal metazoan interpreted as a lophophorate. *Proc. R. Soc. B Biol. Sci.* **282**, 1–10 (2015).

136. Wood, R. & Penny, A. Substrate growth dynamics and biomineralization of an Ediacaran encrusting poriferan. *Proc. R. Soc. B Biol. Sci.* **285**, 20171938 (2018).

**Table S2**: Farms with outcrop of the classic Nama Group. Given codes are a key to Figures S15a-g and do not correspond to official Farm numbers.

| **Number** | **Farms  (South of Osis)** | **Number** | **Farms  (North of Osis)** |
| --- | --- | --- | --- |
| 1 | Binz | 1 | Namakorabis |
| 2 | Blutputz | 2 | Guisis |
| 3 | Maguams | 3 | Abbabis |
| 4 | Chamchawib | 4 | Wagner |
| 5 | Blenheim | 5 | Steizenfels |
| 6 | Saraus | 6 | Nauzerus |
| 7 | Kosos | 7 | Nuwedam |
| 8 | Wittmanshaar | 8 | Diamant |
| 9 | Dabis | 9 | Klein Aub |
| 10 | Volkerust | 10 | Abendruhe |
| 11 | Auas | 11 | Die Vake |
| 12 | Helmeringhausen | 12 | Arbeid Adek |
| 13 | Auas sud | 13 | Aais |
| 14 | Kunjas | 14 | Gneisab |
| 15 | Goais | 15 | Remhoogte |
| 16 | Corunna | 16 | Noab |
| 17 | Rotterdam | 17 | Ebenhout |
| 18 | Frisgewaagd | 18 | Oos |
| 19 | Gamochas | 19 | Tsabisis 340 |
| 20 | Karadaus | 20 | Blasskranz |
| 21 | Mooifontein | 21 | Driedoornvlakte |
| 22 | Stockdale | 22 | Cambells Aub |
| 23 | Nabibis | 23 | Dikdoorn |
| 24 | Hoamoed | 24 | Diep Rivier |
| 25 | Nuweplaas | 25 | Weltevrede 402 |
| 26 | Ausis | 26 | Tsams West |
| 27 | Chamis sud | 27 | Tsams Ost |
| 28 | Soetdoring | 28 | Naukluft |
| 29 | Tiras | 29 | Bullsport |
| 30 | Neisip | 30 | Kambes |
| 31 | Twyfel | 31 | Rietoog |
| 32 | Wegkruip | 32 | Rooidrai |
| 33 | Zuurberg | 33 | Gannavlak |
| 34 | Dreylingen | 34 | Kobas |
| 35 | Bossie | 35 | Klein Angous |
| 36 | Untersee | 36 | Khos |
| 37 | Tokio | 37 | Ghaap |
| 38 | Pronksberg | 38 | Gavind |
| 39 | Duinsig | 39 | Safneck |
| 40 | Aris | 40 | Sand Rivier |
| 41 | Hansburg | 41 | Schlip |
| 42 | Umub | 42 | Slangkolk |
| 43 | Oberndorf | 43 | Blauputz |
| 44 | Olams | 44 | Rondebos |
| 45 | Ganikeis (/Geinochas) | 45 | Chauchab |
| 46 | Radfordsputs and Bree-rivier | 46 | Auchabmund |
| 47 | Carolinahof | 47 | Gaibes |
| 48 | Dorpsgrood (Townlands) and Bethanie | 48 | Zaugab |
| 49 | Wasserfall | 49 | Aries |
| 50 | Dorpsig | 50 | Draaihoek |
| 51 | Augustfelde | 51 | Vingerbreek |
| 52 | Kwessieputs | 52 | Kanaams |
| 53 | Sonderwater | 53 | Arurueis |
| 54 | Vrede | 54 | Kreyrivier |
| 55 | Kloofland | 55 | Fahlhuk |
| 56 | Klipdrif | 56 | Niep |
| 57 | Florsheim | 57 | Schlip Mundung |
| 58 | Hunsruck | 58 | De Hoop |
| 59 | Schakalskuppe | 59 | Kamaseb |
| 60 | Weltevrede | 60 | Herma |
| 61 | Kuibis nord | 61 | Nooitverwag |
| 62 | Nord Klein | 62 | Gamis Nord |
| 63 | Riverside | 63 | Gamis Ost |
| 64 | Schwarzkuppe | 64 | Gamis |
| 65 | Soromaas | 65 | Corabasin |
| 66 | Gangeis | 66 | Verlos |
| 67 | Plateau | 67 | Namtsis |
| 68 | Geelschaap | 68 | Swartkobus |
| 69 | Doorns | 69 | Goabosoah |
| 70 | Kalkfontein | 70 | Van Wyk |
| 71 | Kuibis sud | 71 | Wes Heitzwasies |
| 72 | Macduffs Castle | 72 | Arusis |
| 73 | Buchholz west | 73 | Varkboseh |
| 74 | Buchholzbrunn | 74 | Dirichas |
| 75 | Isaaksbrunn | 75 | Schadeck |
| 76 | Kosis | 76 | Lomoenputs |
| 77 | Schnepfenrivier | 77 | Ubusis |
| 78 | Aar | 78 | Neu Onis |
| 79 | Tsachanabis | 79 | Felseneck |
| 80 | Shanghai | 80 | Goede Hoop |
| 81 | Akam | 81 | Urikos |
| 82 | Uitviug | 82 | Neuras |
| 83 | Brackwasser | 83 | Onis |
| 84 | Simplon | 84 | Kyffhauser |
| 85 | Kesslersbrunn | 85 | Haruchas |
| 86 | Kanas | 86 | Kuderup |
| 87 | Kaalvlakte | 87 | Nudaus |
| 88 | Arasab | 88 | Lahnstein |
| 89 | Hoogland | 89 | Urusis |
| 90 | Kalkrand | 90 | Nomtsas |
| 91 | Nastom | 91 | Narobmund |
| 92 | Harris | 92 | Auros West |
| 93 | Aukum | 93 | Hauchabfontein |
| 94 | Heigums | 94 | Donker Gange |
| 95 | Koppie | 95 | Zebra River |
| 96 | Rietputs | 96 | Friedland |
| 97 | Hudab West | 97 | Kamkas |
| 98 | Irene | 98 | Kameelberg |
| 99 | West Feldschuhhorn | 99 | Gorges |
| 100 | Sandverhaar | 100 | Karab |
| 101 | Feldschuhhorn Ost | 101 | Marlon Reitz |
| 102 | Vogelstrausskluft | 102 | Kabib |
| 103 | Arulot | 103 | Sandhof |
| 104 | Kokerboomkloof | 104 | Wildpark |
| 105 | Sabidas (west side of farm used to be Raraugabib) | 105 | Ober Packriem |
| 106 | Diamantpoort | 106 | Gluckauf |
| 107 | Haaswater | 107 | Odette |
| 108 | Waldsee | 108 | Poburke |
| 109 | Tweespruit | 109 | Mooirivier |
| 110 | Grens | 110 | Hoogland |
| 111 | Pockenbank | 111 | Theronsterg |
| 112 | Ruspunt | 112 | Hoheacht |
| 113 | Rooiberg | 113 | Niederhagen |
| 114 | Rooipunt | 114 | Nutupsdrift |
| 115 | Geigoab | 115 | Namseb |
| 116 | Totem | 116 | Townlands - Maltahohe |
| 117 | Inachab | 117 | Sandpforte |
| 118 | Anib | 118 | Kampe |
| 119 | Anusi | 119 | Omkyk |
| 120 | Kliphoek | 120 | Zwartmodder |
| 121 | Kanies | 121 | Uitkoms |
| 122 | Blauputs | 122 | Kalkhugel |
| 123 | Nuichas | 123 | Halifax |
| 124 | Kaalberg | 124 | Grootplaats |
| 125 | Sandykop | 125 | Dawet |
| 126 | Swartkloofberg | 126 | Kachauchab |
| 127 | Swartpunt | 127 | Karichab |
| 128 | Tierkloof | 128 | Breckhorn |
| 129 | Geelperdshoek | 129 | Zaris |
| 130 | Piet-se Puts | 130 | Steinfeld |
| 131 | Nuwerus | 131 | Bergplaas |
| 132 | Abos | 132 | Noib |
| 133 | Quaggaspoort | 133 | Plattfontein |
| 134 | Huns | 134 | Christiania |
| 135 | Hope | 135 | Burgsdorf |
| 136 | Zaracheibis | 136 | Garub |
| 137 | Churutabis - Sonntagsbrunn | 137 | Hohenfelde |
| 138 | Nord Witputs | 138 | Nam |
| 139 | Sud Witputs | 139 | Gorab |
| 140 | Aub | 140 | Keerom |
| 141 | Kolke | 141 | Aukens |
| 142 | Zebrafontein | 142 | Portjes |
| 143 | Arimas | 143 | Tourlosie |
| 144 | Uitsig | 144 | Grootfontein 91 |
| 145 | Augurabis | 145 | Moreson |
| 146 | Leyerbreek | 146 | Haseweb |
| 147 | Einaug | 147 | Shakalslust |
| 148 | Gabasis Ost | 148 | Nord Schwarzrand |
| 149 | Louisenhohe | 149 | Nooitgedag |
| 150 | Steinfeld | 150 | Rooiberg |
| 151 | Narnbaes | 151 | Nord Kleinfontein |
| 152 | Aikanes | 152 | Kleinfontein |
| 153 | Altdorn 32 | 153 | Eisenach |
| 154 | Arep | 154 | Aandrus |
| 155 | Nordeck | 155 | Sandrucken |
| 156 | Autsas | 156 | Ellsenhof |
| 157 | Zukous | 157 | Naudaus |
| 158 | Hoas Nord | 158 | Rooiberg Sud |
| 159 | Hoas | 159 | Schwarzkuppe |
| 160 | Wolfsschlucht | 160 | Zugspitze |
| 161 | Karenhork | 161 | Wartburg |
| 162 | Schwarzkupper | 162 | Amhub |
| 163 | Stamprivier | 163 | Stubbenkammer |
| 164 | Holoog |  |  |
| 165 | Tsawisis |  |  |
| 166 | Grundorn |  |  |
| 167 | Holoogberg |  |  |
| 168 | Karios |  |  |
| 169 | Geiaus |  |  |
| 170 | Altdorn 3 |  |  |
| 171 | Kanebis |  |  |
| 172 | Frankfurt |  |  |
| 173 | Rotegab |  |  |
| 174 | Mooiplaats |  |  |
| 175 | Weltevrede 302 |  |  |
| 176 | Bergzicht |  |  |
| 177 | Goedgevonder |  |  |
| 178 | Geis |  |  |
| 179 | Kanus |  |  |
| 180 | Spes Bona |  |  |
| 181 | Nanzes |  |  |
| 182 | Bismarckaue |  |  |
| 183 | Liebenrust |  |  |
| 184 | Genadenda |  |  |
| 185 | Us |  |  |
| 186 | Realkoppe |  |  |
| 187 | Maradas |  |  |
| 188 | Groenrivier |  |  |
| 189 | Hauchanas |  |  |
| 190 | Vredenhof |  |  |
| 191 | Uitkomst |  |  |
| 192 | Duurdrift Nord |  |  |
| 193 | Ariams |  |  |
| 194 | Stinkdorn |  |  |
| 195 | Garub |  |  |
| 196 | Nukois |  |  |
| 197 | Kralkuft |  |  |
| 198 | Garis |  |  |
| 199 | Sandmund |  |  |
| 200 | Kachena |  |  |
| 201 | Krantzberg |  |  |
| 202 | Geltsanes |  |  |
| 203 | Sandmodder |  |  |
| 204 | Naauwpoort |  |  |
| 205 | Goa-ab |  |  |
| 206 | Khorrobees |  |  |
| 207 | Amas |  |  |
| 208 | Kubub West |  |  |
| 209 | Kubub Ost |  |  |
| 210 | Nieuwefontein |  |  |
| 211 | Oab |  |  |
| 212 | Karob |  |  |
| 213 | Uheib |  |  |
| 214 | Tzamab Groendorn |  |  |
| 215 | Vlissingen |  |  |
| 216 | Austerlitz |  |  |
| 217 | Kentucky |  |  |
| 218 | Bockiesbank Ost |  |  |
| 219 | Elansdraai |  |  |
| 220 | Heiragabis |  |  |
| 221 | Blyderverwacht |  |  |
| 222 | Jericho |  |  |
| 223 | Platrand |  |  |
| 224 | Border |  |  |
| 225 | Witkop |  |  |
| 226 | Nababis |  |  |
| 227 | Nabas |  |  |
| 228 | Ukamas |  |  |
| 229 | Nakop |  |  |

**Nama Group Farm Map (Figures S15a -S15g)**The following maps were redrawn using a combination of GoogleEarth, Microsoft Powerpoint and numerous Namibian geological maps (1:1000000, 1963, 1978, and revised 1980 editions, 1:250000 Mariental 2416, and Rehoboth 2316, Geological Survey of Namibia, Ministry of Mines and Energy)


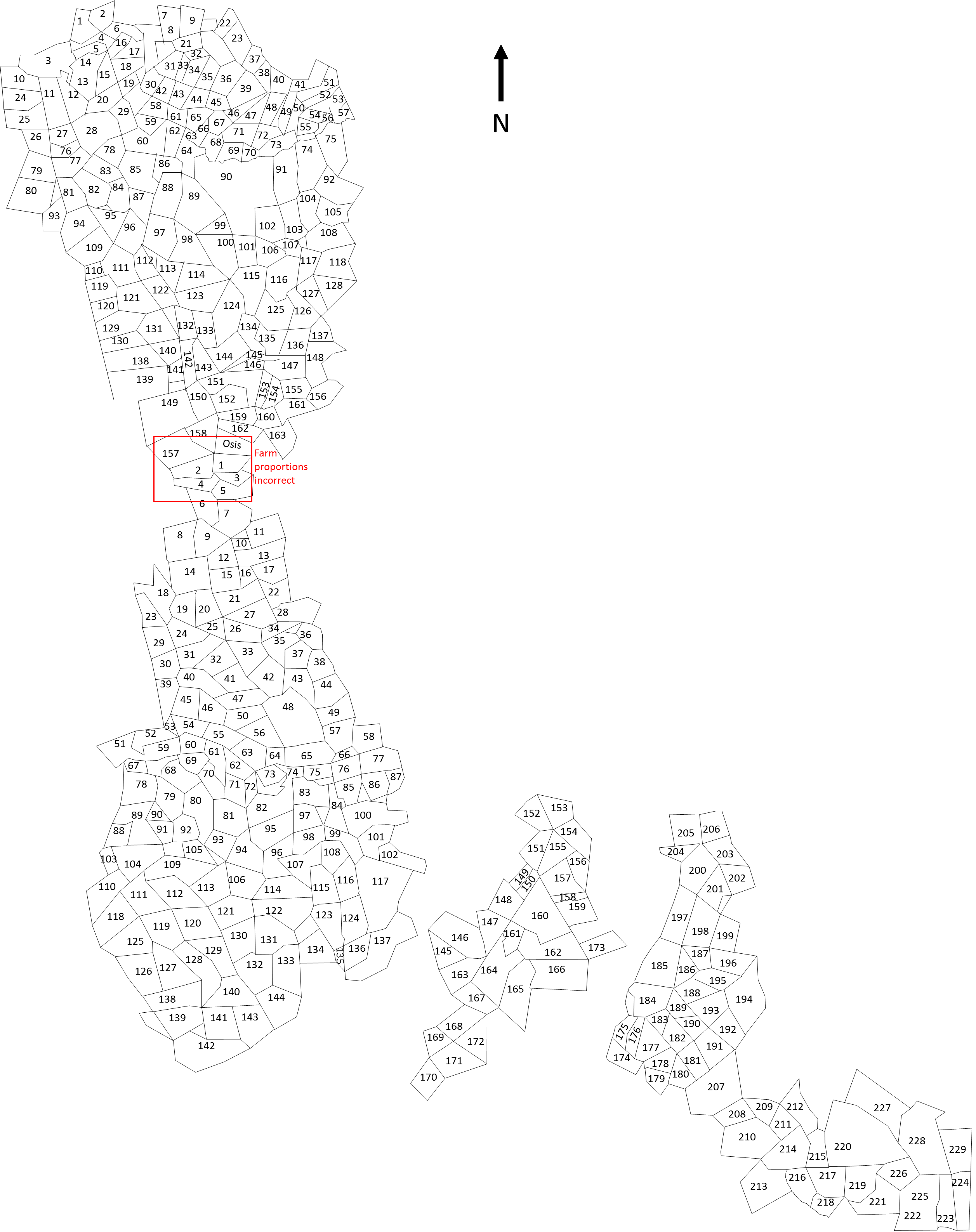


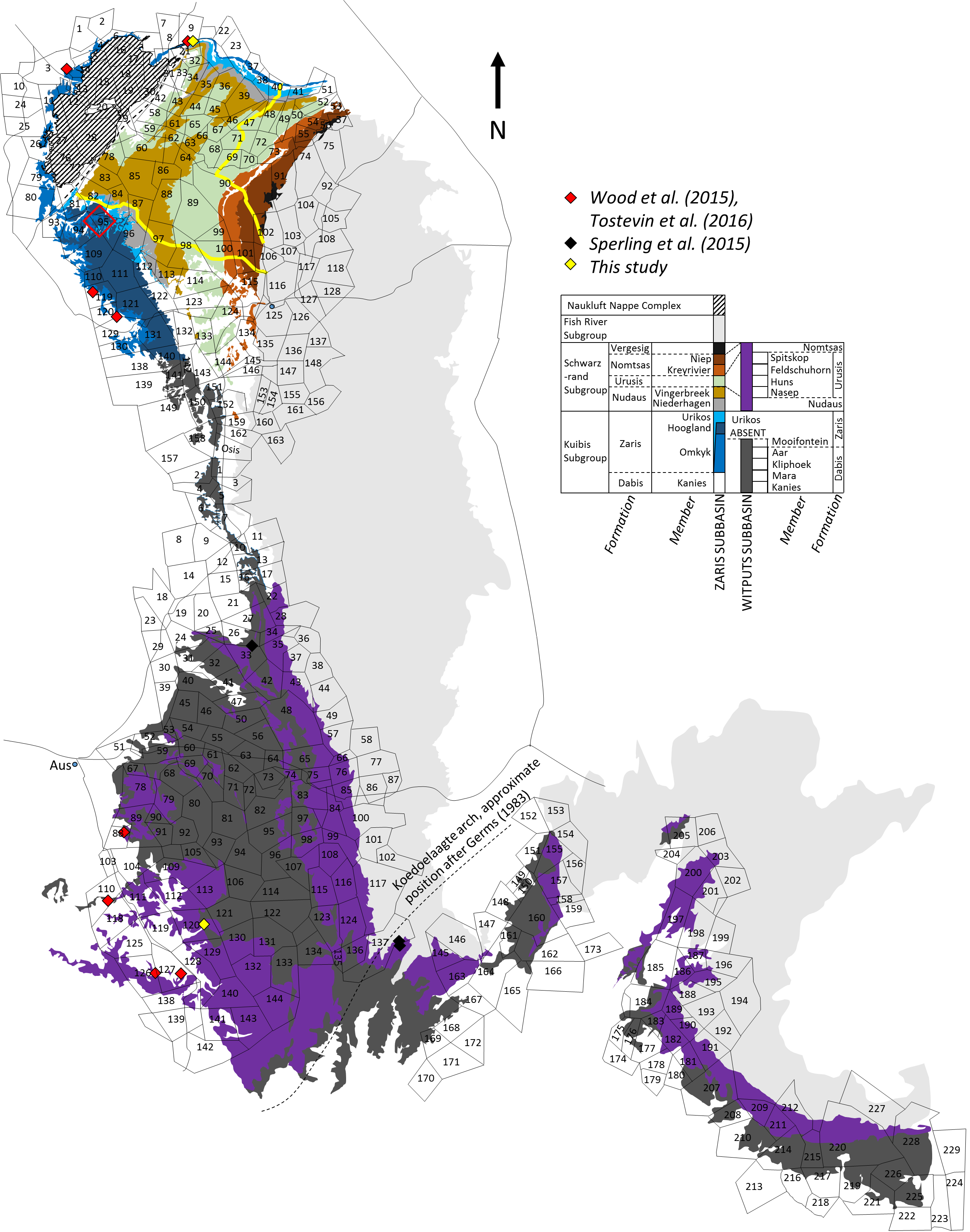


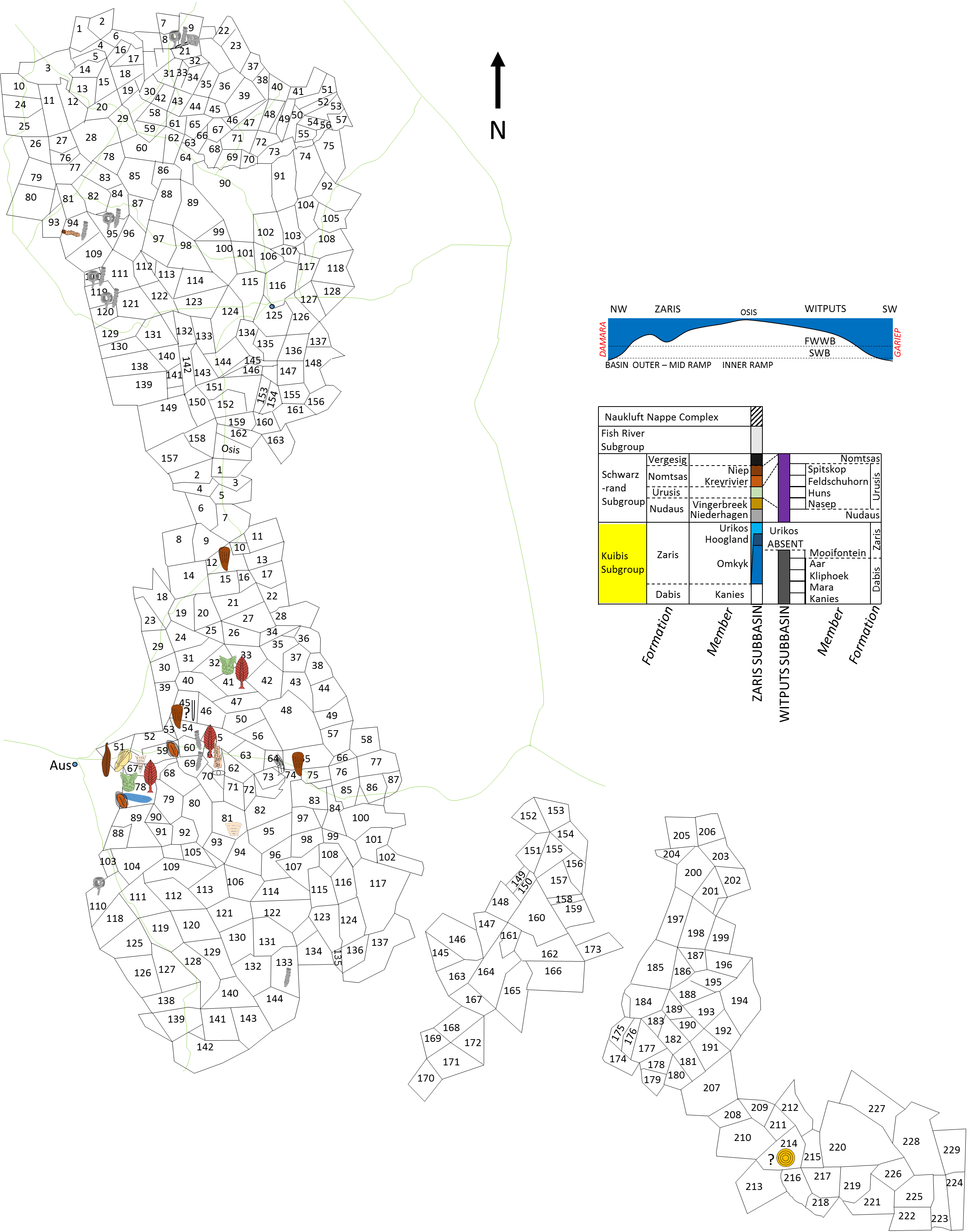


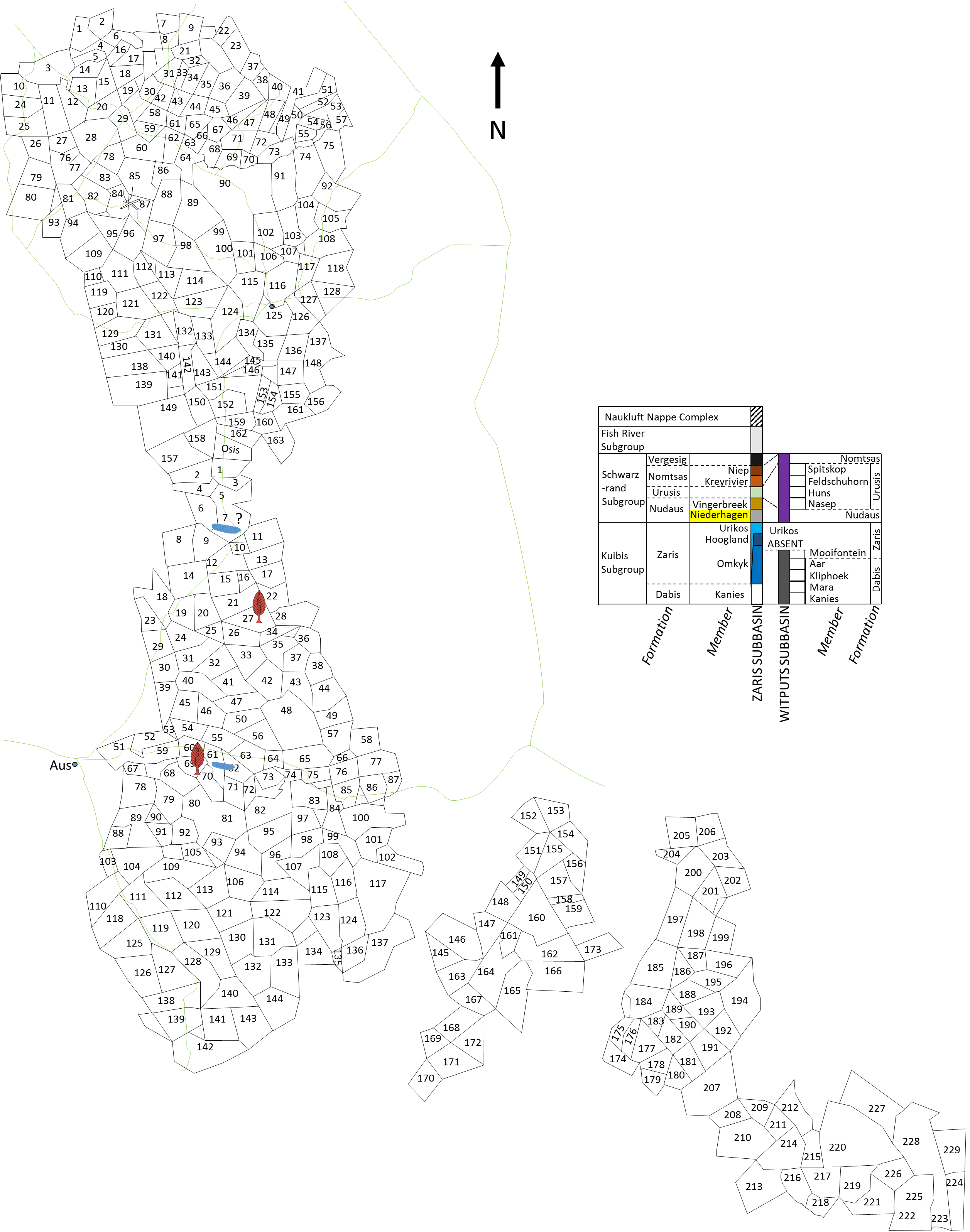


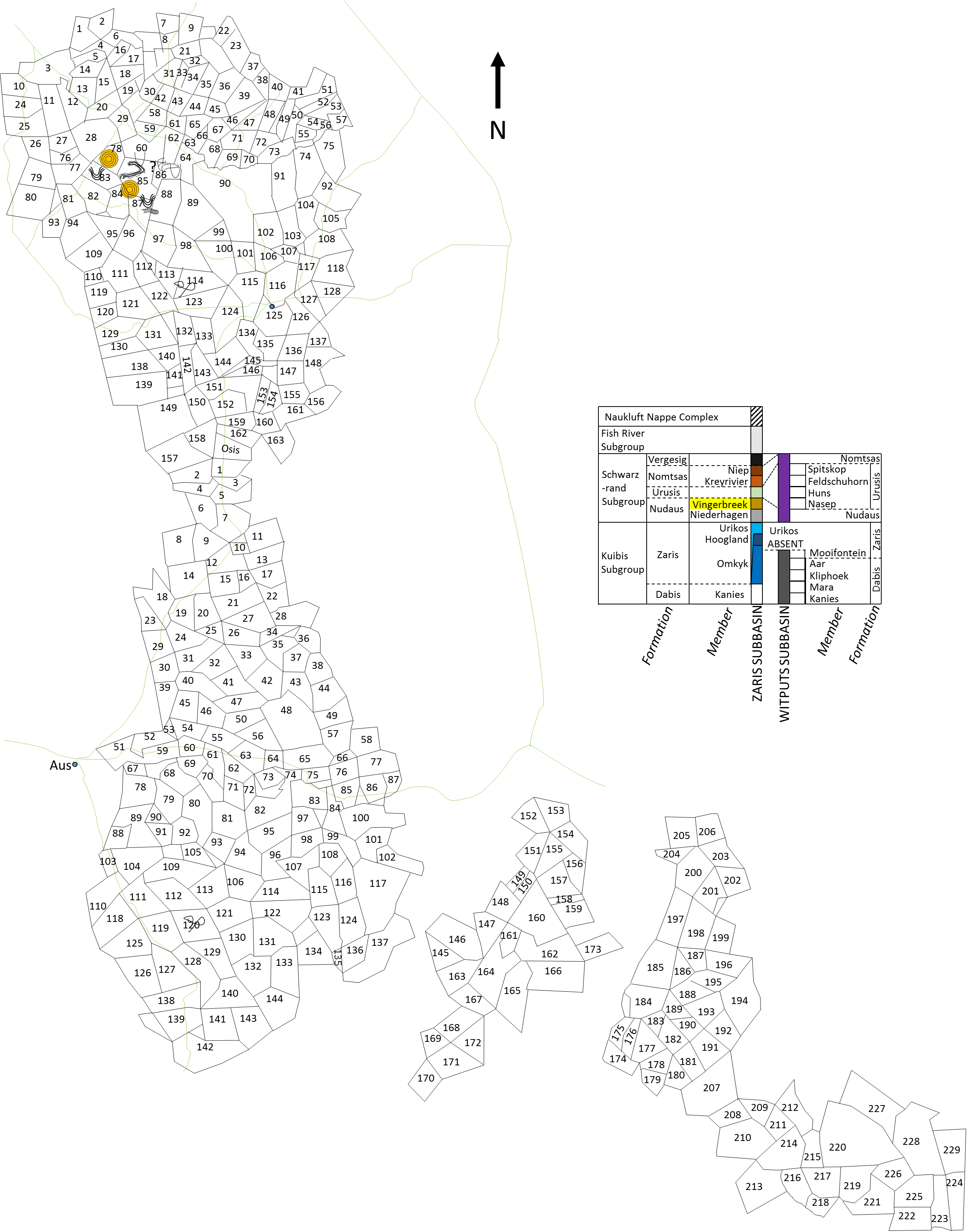


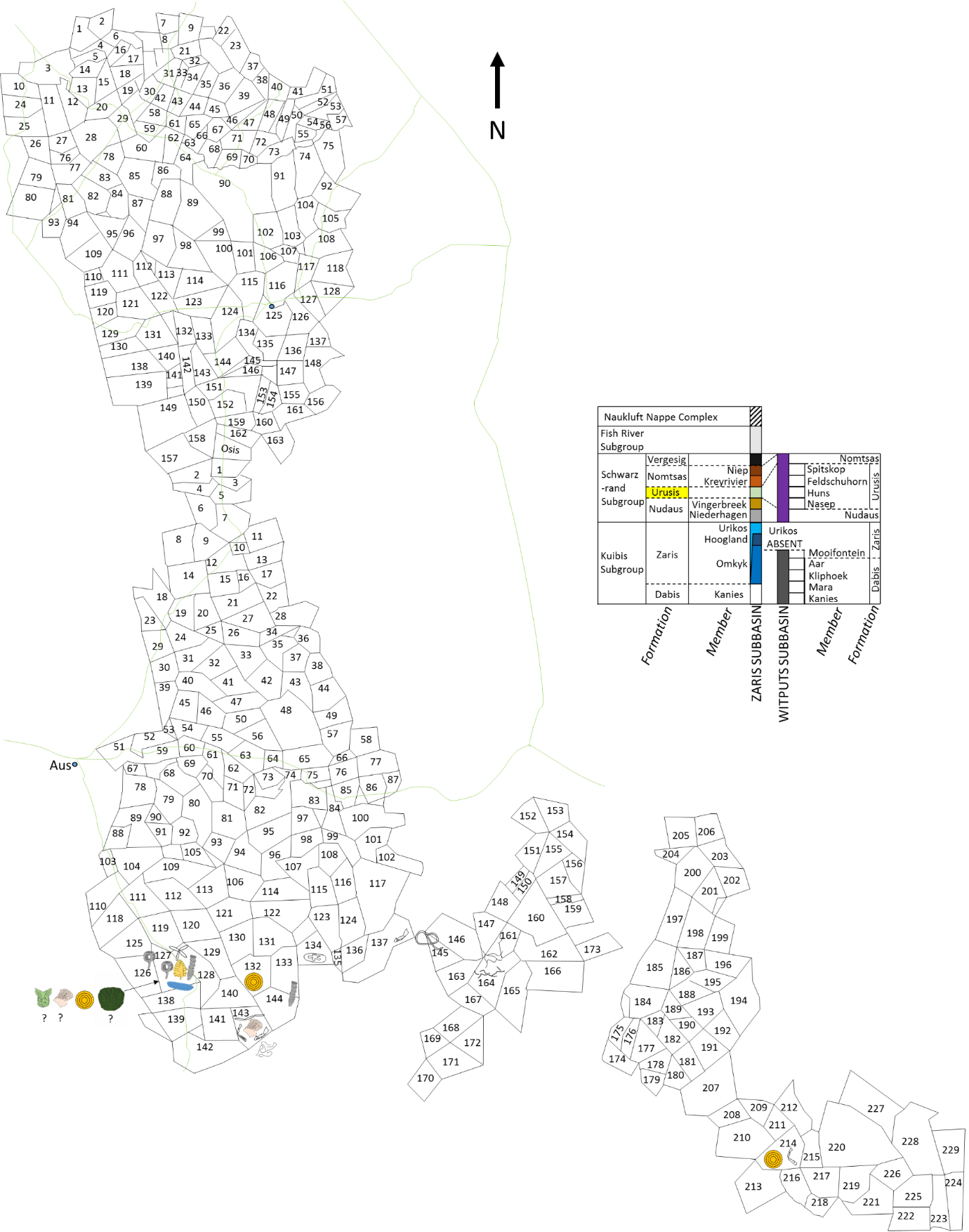


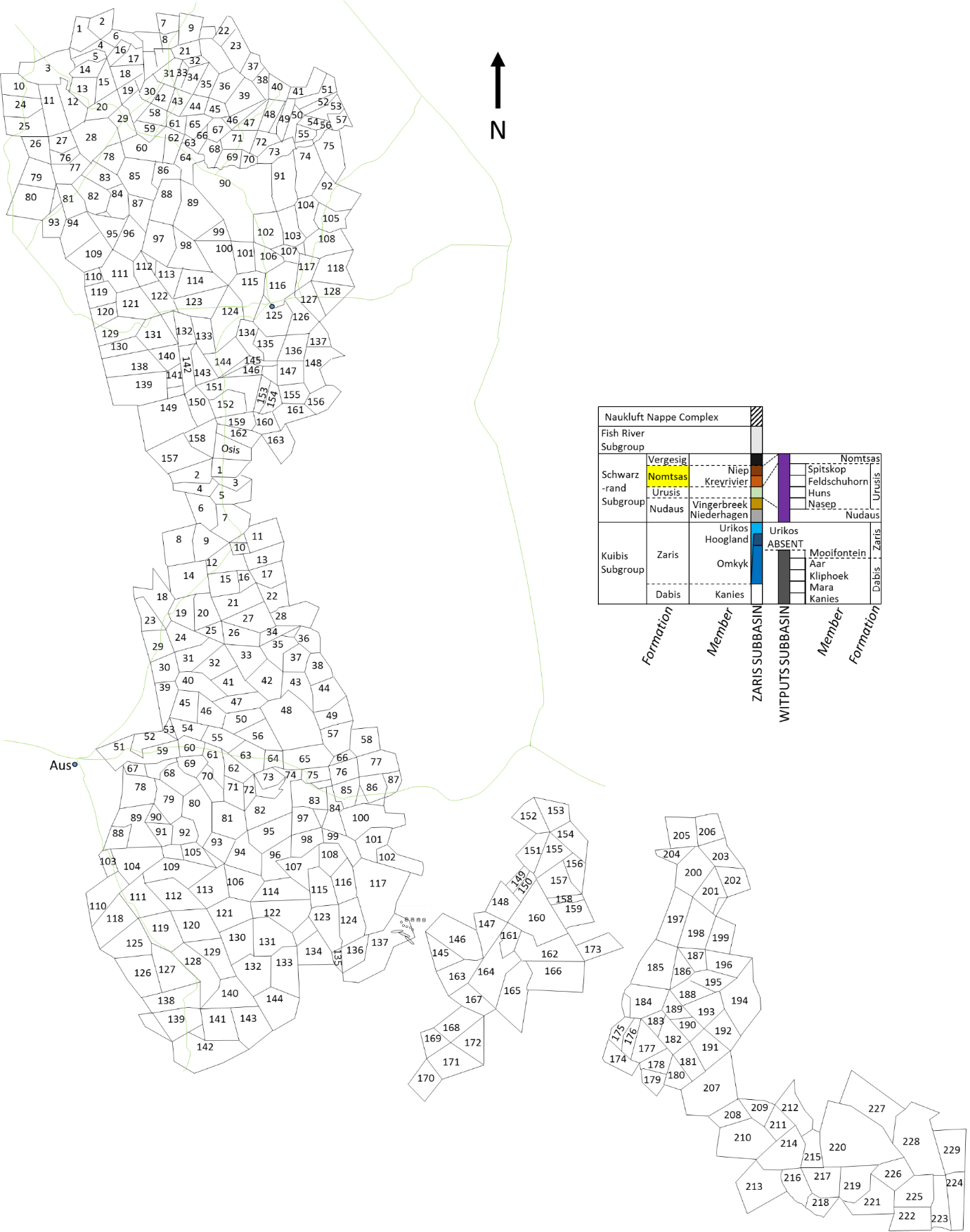


**Table S3:** Major element data. Full data spreadsheet, incorporating published data and rescaled sample position is available upon request.

| **Sample ID** | **Section height (m)** | **Total height (m)** | **Ramp position** | **Al (wt%)** | **Ca (wt%)** | **Fe_T_ (wt%)** | **K (wt%)** | **Mn (ppm)** | **Na (wt%)** | **P (ppm)** | **Ti (ppm)** | **TOC (wt%)** |
| --- | --- | --- | --- | --- | --- | --- | --- | --- | --- | --- | --- | --- |
| **Farm Swartpunt, 27°28'25.98"S, 16°41'45.12"E** | | | | | | | | | | | | |
| ***Schwarzrand Subgroup, Urusis Formation, Spitskop Member*** | | | | | | | | | | | | |
| SWP2/12 | 87.24 | 1915 | Outer | 7.10 |  |  |  | 318 |  | 922 |  | 0.08 |
| SWP2/10 | 78.44 | 1895 | Outer | 6.44 |  |  |  | 857 |  | 715 |  | 0.07 |
| SWP2/8 | 74.74 | 1885 | Outer | 8.55 |  |  |  | 438 |  | 750 |  | 0.08 |
| SWP2/7 | 72.64 | 1880 | Outer | 8.18 |  |  |  | 830 |  | 825 |  | 0.09 |
| SWP2/6 | 70.24 | 1875 | Outer | 7.63 |  |  |  | 502 |  | 724 |  | 0.07 |
| SWP2/5 | 69.04 | 1870 | Outer | 7.69 |  |  |  | 350 |  | 704 |  | 0.08 |
|  |  |  |  |  |  |  |  |  |  |  |  |  |
| **Farm Swartkloofberg (Pinnacle Reefs locality), 27°26'48.24"S, 16°33'43.62"E** | | | | | | | | | | | | |
| ***Schwarzrand Subgroup, Urusis Formation, Spitskop Member*** | | | | | | | | | | | | |
| SK1/10 | 89 | 1740 | Outer | 8.95 |  | 5.25 |  | 377 |  | 858 |  | 0.09 |
| SK1/9 | 75 | 1730 | Outer | 7.46 |  | 4.16 |  | 259 |  | 877 |  | 0.07 |
| ***Schwarzrand Subgroup, Urusis Formation, Feldschuhhorn Member*** | | | | | | | | | | | | |
| SK1/8 | 47.75 | 1728 | Mid | 9.63 |  | 5.29 |  | 624 |  | 799 |  | 0.07 |
| SK1/7 | 44.55 | 1710 | Mid | 9.05 |  | 5.37 |  | 683 |  | 788 |  | 0.09 |
| SK1/6 | 34.95 | 1700 | Mid | 9.05 |  | 5.29 |  | 705 |  | 797 |  | 0.19 |
| SK1/5 | 23.75 | 1690 | Mid | 8.33 |  | 4.63 |  | 1064 |  | 803 |  | 0.08 |
| SK1/4 | 15.75 | 1670 | Mid | 8.70 |  | 4.50 |  | 312 |  | 758 |  | 0.07 |
| SK1/3 | 10.15 | 1650 | Mid | 9.13 |  | 4.81 |  | 317 |  | 460 |  | 0.07 |
| SK1/1 | 1.5 | 1624 | Mid | 7.76 |  | 3.91 |  | 743 |  | 637 |  | 0.07 |
|  |  |  |  |  |  |  |  |  |  |  |  |  |
| **Sample ID** | **Section height (m)** | **Total height (m)** | **Ramp position** | **Al (wt%)** | **Ca (wt%)** | **Fe_T_ (wt%)** | **K (wt%)** | **Mn (ppm)** | **Na (wt%)** | **P (ppm)** | **Ti (wt%)** | **TOC (wt%)** |
| **Farm Kliphoek, 27°17'05.28"S, 16°46'47.58"E** | | | | | | | | | | | | |
| ***Schwarzrand Subgroup, Nudaus Formation, Vingerbreek Member*** | | | | | | | | | | | | |
| NUD-20 | 214 | 966 | Mid | 7.55 | 0.21 | 5.54 | 2.32 | 477 | 1.50 | 792 | 0.21 |  |
| NUD-19 | 188 | 964 | Mid | 3.70 | 0.14 | 19.27 | 2.80 |  | 1.49 | 207 | 0.10 |  |
| NUD-18 | 178 | 963.5 | Mid | 5.65 | 0.20 | 24.59 | 2.12 |  | 1.14 | 595 | 0.12 |  |
| NUD-16 | 154 | 952.5 | Mid | 7.46 | 0.29 | 22.08 | 1.90 |  |  |  | 0.20 |  |
| NUD-15 | 144 | 947 | Mid | 8.72 | 0.30 | 6.99 | 1.88 |  |  |  | 0.28 |  |
| NUD-14 | 134 | 941.5 | Mid | 7.39 | 0.22 | 17.55 | 1.78 |  |  |  | 0.19 |  |
| NUD-12 | 114 | 930.5 | Mid | 6.44 | 0.22 | 25.05 | 1.62 |  |  |  | 0.16 |  |
| NUD-11 | 104 | 925 | Mid | 6.50 | 0.11 | 28.69 | 2.00 | 883 | 0.03 | 308 | 0.17 |  |
| NUD-10 | 94 | 919.5 | Mid | 6.72 | 0.20 | 28.78 | 1.81 |  |  |  | 0.16 |  |
| NUD-9 | 84 | 914 | Mid | 5.25 | 0.16 | 18.87 | 1.44 | 56 | 0.35 | 730 | 0.13 |  |
| NUD-8 | 74 | 908.5 | Mid | 9.22 | 0.13 | 5.96 | 2.67 | 869 | 0.84 | 259 | 0.24 |  |
| NUD-7 | 64 | 903 | Mid | 9.82 | 0.15 | 6.34 | 2.96 | 524 | 0.17 | 169 | 0.27 |  |
| NUD-6 | 53 | 897.5 | Mid | 7.04 | 0.16 | 27.35 | 2.02 | 698 | 0.15 | 349 | 0.15 |  |
| NUD-5 | 43 | 892 | Mid | 6.49 | 0.27 | 29.77 | 1.83 |  |  |  | 0.16 |  |
| NUD-4 | 31 | 886.5 | Mid | 10.21 | 0.23 | 4.94 | 2.33 | 677 | 0.80 | 891 | 0.26 | 0.08 |
| NUD-3 | 24 | 881 | Mid | 11.27 | 0.16 | 5.07 | 2.87 | 912 | 0.68 | 397 | 0.28 | 0.08 |
| NUD-2 | 21 | 875 | Mid | 11.15 | 0.11 | 8.17 | 3.07 | 914 | 1.04 | 243 | 0.31 | 0.09 |
| NUD-1 | 10 | 870 | Mid | 9.13 | 0.14 | 9.41 | 2.07 | 686 | 1/49 | 540 | 0.18 | 0.10 |
|  |  |  |  |  |  |  |  |  |  |  |  |  |
|  |  |  |  |  |  |  |  |  |  |  |  |  |
|  |  |  |  |  |  |  |  |  |  |  |  |  |
| **Sample ID** | **Co-ordinates** | **Total height (m)** | **Ramp position** | **Al (wt%)** | **Ca (wt%)** | **Fe_T_ (wt%)** | **K (wt%)** | **Mn (ppm)** | **Na (wt%)** | **P (ppm)** | **Ti (wt%)** | **TOC (wt%)** |
| **Road transect D850** | | | | | | | | | | | | |
| ***Schwarzrand Subgroup, Nomtsas Formation, Niep Member*** | | | | | | | | | | | | |
| S15 | 24°42'56.22"S, 16°54'48.54"E | 2080.27 | Shallow inner |  |  | 3.20 |  |  |  |  |  |  |
| S14 | 24°41'16.68"S, 16°52'45.12"E | 2055.88 | Shallow inner |  |  | 2.50 |  |  |  |  |  |  |
| ***Schwarzrand Subgroup, Urusis Formation, undefined member*** | | | | | | | | | | | | |
| S13 | 24°40'49.08"S, 16°47'03.96"E | 1956.31 | Shallow inner |  |  | 2.41 |  |  |  |  |  |  |
| D850-1 | 24°40'53.52"S, 16°46'15.78"E | 1852.67 | Shallow inner | 6.78 | 0.39 | 3.89 | 2.21 | 526 | 0.96 | 605 | 0.23 |  |
| S12a | 24°40'49.20"S, 16°44'17.10"E | 1775.45 | Shallow inner |  |  | 1.64 |  |  |  |  |  |  |
| S12b | 24°40'49.20"S, 16°44'17.10"E | 1775.45 | Shallow inner | 4.39 |  | 3.37 |  | 411 |  | 1245 |  | 0.06 |
| D850-2 | 24°39'40.62"S, 16°42'22.44"E | 1584.44 | Shallow inner | 7.85 | 0.42 | 5.87 | 2.62 | 549 | 0.91 | 676 | 0.30 |  |
| S11 | 24°39'35.52"S, 16°42'12.54"E | 1492.99 | Shallow inner |  |  | 2.06 |  |  |  |  |  |  |
| D850-4 | 24°37'59.52"S, 16°40'11.22"E | 1316.20 | Shallow inner | 5.80 | 0.29 | 3.16 | 1.60 | 352 | 1.20 | 763 | 0.17 |  |
| D850-6 | 24°37'51.96"S, 16°38'02.46"E | 1142.46 | Shallow inner | 6.68 | 0.36 | 3.81 | 2.25 | 243 | 1.01 | 578 | 0.26 |  |
| S10 | 24°37'54.54"S, 16°36'22.92"E | 1051.02 | Shallow inner |  |  | 5.69 |  |  |  |  |  |  |
| ***Schwarzrand Subgroup, Nudaus Formation, Vingerbreek Member*** | | | | | | | | | | | | |
| S9 | 24°37'51.54"S, 16°33'23.16"E | 965 | Shallow inner |  |  | 3.77 |  |  |  |  |  |  |
| S8 | 24°36'38.76"S, 16°30'23.76"E | 930 | Shallow inner | 8.28 |  | 3.82 |  | 260 |  | 132 |  | 0.10 |
| S7 | 24°34'06.72"S, 16°28'40.86"E | 900 | Shallow inner |  |  | 2.98 |  |  |  |  |  |  |
| S6 | 24°32'19.02"S, 16°26'21.30"E | 870 | Shallow inner |  |  | 3.13 |  |  |  |  |  |  |
| ***Schwarzrand Subgroup, Nudaus Formation, Niederhagen Member*** | | | | | | | | | | | | |
| S5 | 24°29'50.76"S, 16°25'33.36"E | 865 | Deep inner | 5.86 |  | 3.68 |  | 205 |  | 311 |  | 0.09 |
| S4 | 24°28'55.14"S, 16°23'11.94"E | 780 | Deep inner |  |  | 3.11 |  |  |  |  |  |  |
| ***Kuibis Subgroup, Zaris Formation, Urikos Member*** | | | | | | | | | | | | |
| S3 | 24°29'18.96"S, 16°20'30.12"E | 760 | Mid |  |  | 3.73 |  |  |  |  |  |  |
| **Sample ID** | **Co-ordinates** | **Total height (m)** | **Ramp position** | **Al (wt%)** | **Ca (wt%)** | **Fe_T_ (wt%)** | **K (wt%)** | **Mn (ppm)** | **Na (wt%)** | **P (ppm)** | **Ti (wt%)** | **TOC (wt%)** |
| S2a | 24°29'09.72"S, 16°19'13.92"E | 680 | Mid |  |  | 3.56 |  |  |  |  |  |  |
| S2b | 24°29'09.72"S, 16°19'13.92"E | 680 | Mid |  |  | 6.68 |  |  |  |  |  |  |
| S1 | 24°29'09.72"S, 16°19'13.92"E | 660 | Mid | 9.22 |  | 6.49 |  | 352 |  | 338 |  | 0.08 |
|  |  |  |  |  |  |  |  |  |  |  |  |  |
| **Road transect C14** | | | | | | | | | | | | |
| ***Schwarzrand Subgroup, Nomtsas Formation, Niep Member*** | | | | | | | | | | | | |
| D860-21 | 24°34'58.98"S, 16°55'49.14"E | 2080.27 | Shallow inner | 8.37 | 0.25 | 5.23 | 2.96 | 418 | 1.09 | 691 | 0.30 |  |
| D860-20 | 24°28'47.04"S, 16°52'56.58"E | 2045.72 | Shallow inner | 7.36 | 0.35 | 3.58 | 2.05 | 501 | 2.02 | 1051 | 0.28 | 0.07 |
| D860-19 | 24°26'49.68"S, 16°52'06.48"E | 2028.45 | Shallow inner | 7.57 | 0.25 | 4.46 | 2.73 | 590 | 0.98 | 543 | 0.28 |  |
| ***Schwarzrand Subgroup, Nomtsas Formation, Kreyrivier Member*** | | | | | | | | | | | | |
| D860-18 | 24°25'24.60"S, 16°50'42.78"E | 2022.35 | Shallow inner | 6.87 | 0.38 | 2.94 | 1.60 | 511 | 1.49 | 1204 | 0.26 | 0.10 |
|  |  |  |  |  |  |  |  |  |  |  |  |  |
| **Road transect D860** | | | | | | | | | | | | |
| ***Schwarzrand Subgroup, Urusis Formation, undefined member*** | | | | | | | | | | | | |
| D860-16 | 24°21'06.00"S, 16°46'10.68"E | 1451.34 | Shallow inner | 6.63 | 0.33 | 4.45 | 1.86 | 510 | 1.87 | 832 | 0.24 |  |
| D860-15 | 24°19'19.32"S, 16°47'40.92"E | 1374.12 | Shallow inner | 8.31 | 0.39 | 5.16 | 2.57 | 451 | 1.37 | 723 | 0.34 |  |
| D860-14 | 24°14'54.48"S, 16°51'21.30"E | 1344.65 | Shallow inner | 8.60 | 0.33 | 5.38 | 2.84 | 488 | 1.35 | 768 | 0.35 |  |
| D860-10 | 24°10'35.34"S, 16°51'13.68"E | 1193.26 | Shallow inner | 6.29 | 0.19 | 3.47 | 1.73 | 292 | 1.02 | 463 | 0.18 |  |
| D860-9 | 24°10'41.22"S, 16°51'56.34"E | 1157.70 | Shallow inner | 9.49 | 0.25 | 4.90 | 2.90 | 377 | 1.23 | 868 | 0.36 | 0.10 |
| D860-8 | 24°10'29.40"S, 16°52'42.06"E | 1112.99 | Shallow inner | 6.62 | 0.21 | 4.97 | 1.58 | 336 | 1.56 | 501 | 0.22 |  |
| D860-7 | 24°10'09.90"S, 16°53'19.32"E | 1080.48 | Shallow inner | 7.80 | 0.31 | 5.20 | 2.37 | 333 | 1.56 | 704 | 0.31 |  |
| D860-6 | 24°09'07.08"S, 16°55'25.02"E | 1059.14 | Shallow inner | 5.34 | 0.18 | 3.47 | 1.14 | 232 | 1.09 | 294 | 0.19 |  |
|  |  |  |  |  |  |  |  |  |  |  |  |  |
| **Sample ID** | **Co-ordinates** | **Total height (m)** | **Ramp position** | **Al (wt%)** | **Ca (wt%)** | **Fe_T_ (wt%)** | **K (wt%)** | **Mn (ppm)** | **Na (wt%)** | **P (ppm)** | **Ti (wt%)** | **TOC (wt%)** |
| ***Schwarzrand Subgroup, Nudaus Formation, Vingerbreek Member*** | | | | | | | | | | | | |
| D860-5 | 24°06'57.42"S, 16°58'21.54"E | 950 | Shallow inner | 5.63 | 0.18 | 3.31 | 1.32 | 117 | 0.97 | 223 | 0.18 |  |
| D860-4 | 24°06'54.00"S, 16°58'26.16"E | 890 | Shallow inner | 6.73 | 0.12 | 4.42 | 1.77 | 185 | 0.81 | 96 | 0.22 |  |
| ***Schwarzrand Subgroup, Nudaus Formation, Niederhagen Member*** | | | | | | | | | | | | |
| D860-3 | 24°05'54.90"S, 16°59'36.96"E | 860 | Deep inner | 5.86 | 0.10 | 3.48 | 1.51 | 128 | 0.88 | 60 | 0.18 |  |
| D860-2 | 24°05'51.36"S, 16°59'44.58"E | 840 | Deep inner | 4.88 | 0.10 | 2.58 | 1.12 | 62 | 0.97 | 97 | 0.12 |  |
| D860-1 | 24°05'35.52"S, 16°59'48.06"E | 780 | Deep inner | 5.87 | 0.16 | 2.91 | 1.82 | 82 | 1.17 | 179 | 0.16 |  |
|  |  |  |  |  |  |  |  |  |  |  |  |  |
|  | | | | | | | | | | | | |
|  | | | | | | | | | | | | |
|  |  |  |  |  |  |  |  |  |  |  |  |  |
|  |  |  |  |  |  |  |  |  |  |  |  |  |
|  |  |  |  |  |  |  |  |  |  |  |  |  |
|  | | | | | | | | | | | | |
|  | | | | | | | | | | | | |
|  |  |  |  |  |  |  |  |  |  |  |  |  |
|  |  |  |  |  |  |  |  |  |  |  |  |  |
|  |  |  |  |  |  |  |  |  |  |  |  |  |
|  |  |  |  |  |  |  |  |  |  |  |  |  |
|  |  |  |  |  |  |  |  |  |  |  |  |  |
|  |  |  |  |  |  |  |  |  |  |  |  |  |
|  |  |  |  |  |  |  |  |  |  |  |  |  |
|  | | | | | | | | | | | | |
| **Sample ID** | **Section height (m)** | **Total height (m)** | **Ramp position** | **Al (wt%)** | **Ca (wt%)** | **Fe_T_ (wt%)** | **K (wt%)** | **Mn (ppm)** | **Na (wt%)** | **P (ppm)** | **Ti (wt%)** | **TOC (wt%)** |
| **Farm Swartmodder, 24°53'40.98"S, 16°19'31.02"E** | | | | | | | | | | | | |
| **Kuibis Subgroup, Dabis Formation, Kanies Member** | | | | | | | | | | | | |
| SWM2/3 | 15.5 | 155 | Shallow inner | 4.41 |  | 1.86 |  | 224 |  | 97 |  | 0.06 |
| SWM2/1 | 3 | 140 | Shallow inner | 5.15 |  | 2.00 |  | 323 |  | 837 |  | 0.05 |
|  |  |  |  |  |  |  |  |  |  |  |  |  |
| **Farm Omkyk, 24°48'19.02"S, 16°13'45.00"E** | | | | | | | | | | | | |
| ***Kuibis Subgroup, Zaris Formation, Omkyk Member*** | | | | | | | | | | | | |
| OMK4/1 | 73 | 288 | Deep inner | 2.49 |  | 0.71 |  | 51 |  | 57 |  | 0.04 |
| OMK3/14 | 44 | 241 | Mid | 4.78 |  | 2.03 |  | 54 |  | 1263 |  | 0.06 |
| OMK3/3 | 4.5 | 163 | Mid | 6.60 |  | 1.30 |  | 52 |  | 87 |  | 0.06 |
| OMK3/2 | 3 | 159 | Mid | 4.45 |  | 0.64 |  | 50 |  | 121 |  | 0.05 |
|  | | | | | | | | | | | | |
| **Farm Driedoornvlakte, 23°51.429'S 16°39.637'E** | | | | | | | | | | | | |
| ***Schwarzrand Subgroup, Nudaus Formation, Niederhagen Member*** | | | | | | | | | | | | |
| DV2-23 | 660 | 800 | Mid | 7.93 |  | 4.45 | 2.46 | 320 | 0.51 | 512 | 0.32 |  |
| DV2-24 | 640 | 790 | Mid | 6.57 |  | 3.98 | 1.84 | 116 | 0.65 | 702 | 0.26 |  |
| DV2-25 | 624 | 780 | Mid | 6.75 |  | 3.97 | 1.96 | 134 | 0.56 | 878 | 0.23 |  |
| DV2-26 | 622 | 770 | Mid | 8.30 |  | 4.74 | 2.79 | 192 | 0.35 | 646 | 0.32 |  |
| DV2-27 | 615 | 766 | Mid | 9.20 |  | 4.95 | 3.09 | 289 | 0.32 | 247 | 0.35 |  |
| ***Kuibis Subgroup, Zaris Formation, Urikos Member (above OS2 Unit 3m)*** | | | | | | | | | | | | |
| DV2-28 | 580 | 700 | Mid | 7.48 |  | 3.67 | 2.67 | 111 | 0.83 | 542 | 0.27 |  |
| DV2-30 | 534 | 600 | Mid | 7.60 |  | 4.03 | 2.39 | 186 | 0.72 | 614 | 0.25 |  |
| DV2-29 | 532 | 590 | Mid | 7.18 |  | 4.01 | 2.24 | 195 | 0.91 | 533 | 0.23 |  |
| **Sample ID** | **Section height (m)** | **Total height (m)** | **Ramp position** | **Al (wt%)** | **Ca (wt%)** | **Fe_T_ (wt%)** | **K (wt%)** | **Mn (ppm)** | **Na (wt%)** | **P (ppm)** | **Ti (wt%)** | **TOC (wt%)** |
| DV2-22 | 557 | 585 | Mid | 7.47 |  | 4.12 | 2.12 | 145 | 0.45 | 572 | 0.26 |  |
| DV2-22b | Replicate |  |  | 7.37 | 0.26 | 4.42 | 2.07 | 136 | 0.43 | 566 | 0.26 |  |
| DV2-22c | Replicate |  |  | 7.31 | 0.26 | 4.24 | 2.10 | 147 | 0.44 | 556 | 0.26 |  |
| DV2-22d | Replicate |  |  | 7.29 | 0.26 | 4.21 | 2.10 | 146 | 0.44 | 546 | 0.25 |  |
| DV2-22e | Replicate |  |  | 7.34 | 0.26 | 4.27 | 2.10 | 140 | 0.44 | 563 | 0.23 |  |
| DV2-21 | 555 | 580 | Mid | 7.13 |  | 3.59 | 1.61 | 182 | 0.91 | 500 | 0.24 | 0.09 |
| DV5 | 550 | 520 | Mid | 9.55 |  | 6.03 | 2.97 | 231 | 0.38 | 598 | 0.28 |  |
| DV4 | 548 | 515 | Mid | 6.43 |  | 3.66 | 1.70 | 148 | 1.00 | 479 | 0.23 |  |
| DV3 | 538 | 490 | Mid | 7.12 |  | 4.26 | 1.83 | 135 | 0.86 | 437 | 0.24 |  |
| DV2 | 530 | 465 | Mid | 3.77 |  | 1.99 | 0.58 | 27 | 0.81 | 132 | 0.10 |  |
| DV1 | 528 | 460 | Mid | 4.97 |  | 3.21 | 0.99 | 101 | 0.83 | 253 | 0.16 |  |
| DV2-31 | 510 | 430 | Mid | 6.62 | 0.17 | 3.83 | 1.82 | 128 | 0.69 | 423 | 0.23 |  |
| ***Kuibis Subgroup, Zaris Formation, Urikos Member (down-dip equivalent to OS2 Unit 3m)*** | | | | | | | | | | | | |
| DV2-20 | 470 | 399 | Mid | 9.34 | 0.26 | 5.46 | 2.70 | 251 | 0.79 | 483 | 0.27 | 0.09 |
| DV2-19 | 460 | 394 | Mid | 9.90 | 0.30 | 5.70 | 2.88 | 427 | 0.07 | 681 | 0.27 | 0.09 |
| ***Kuibis Subgroup, Zaris Formation, Urikos Member (down-dip equivalent to OS2 Unit 3i)*** | | | | | | | | | | | | |
| DV2-18 | 450 | 392 | Mid | 9.22 | 0.22 | 4.96 | 3.13 | 428 | 0.73 | 194 | 0.31 |  |
| DV2-17 | 425 | 384 | Mid | 8.83 |  | 5.34 | 3.65 | 15 |  | 228 | 0.38 |  |
| ***Kuibis Subgroup, Zaris Formation, Urikos Member (down-dip equivalent to OS2 Unit 2m)*** | | | | | | | | | | | | |
| DV2-15 | 310 | 350 | Mid | 9.13 |  | 5.12 | 3.35 | 77 | 0.46 | 367 | 0.32 |  |
| DV2-16 | 310 | 350 | Mid | 9.41 | 0.17 | 4.70 | 3.62 | 66 | 0.25 | 289 | 0.29 |  |
| ***Kuibis Subgroup, Zaris Formation, Urikos Member (down-dip equivalent to OS2 Unit 1b)*** | | | | | | | | | | | | |
| DV2-8 | 230 | 325.5 | Mid | 9.45 |  | 4.59 | 3.50 | 61 | 0.50 | 917 | 0.31 |  |
| DV2-14 | 230 | 325.5 | Mid | 9.38 | 0.40 | 4.67 | 3.52 | 83 | 1.21 | 396 | 0.30 |  |
| **Sample ID** | **Section height (m)** | **Total height (m)** | **Ramp position** | **Al (wt%)** | **Ca (wt%)** | **Fe_T_ (wt%)** | **K (wt%)** | **Mn (ppm)** | **Na (wt%)** | **P (ppm)** | **Ti (wt%)** | **TOC (wt%)** |
| DV2-13 | 185 | 313 | Mid | 8.84 | 1.38 | 4.49 | 3.11 | 124 | 1.07 | 338 | 0.31 |  |
| DV2-7 | 179 | 312 | Mid | 9.47 |  | 5.34 | 3.57 | 88 |  | 329 | 0.35 |  |
| DV2-6 | 172 | 308.5 | Mid | 9.66 |  | 4.91 | 3.51 | 60 | 0.25 | 316 | 0.35 |  |
| DV2-12 | 170 | 308 | Mid | 9.49 | 0.48 | 4.73 | 3.64 | 146 | 0.77 | 324 | 0.31 |  |
| ***Kuibis Subgroup, Zaris Formation, Urikos Member (down-dip equivalent to OS2 Unit 1a)*** | | | | | | | | | | | | |
| DV2-11 | 163 | 306 | Mid | 9.64 | 1.07 | 4.87 | 3.62 | 155 | 1.09 | 292 | 0.38 |  |
| DV2-9 | 120 | 293 | Mid | 8.48 |  | 4.65 | 3.06 | 17 | 0.02 | 219 | 0.39 |  |
| DV2-10 | 120 | 293 | Mid | 9.20 |  | 4.15 | 3.49 | 162 | 0.42 | 247 | 0.29 |  |
|  |  |  |  |  |  |  |  |  |  |  |  |  |
| **Farm Arasab, 26°57'48.54"S, 16°27'25.98"E** | | | | | | | | | | | | |
| ***Kuibis Subgroup, Dabis Formation, Mara Member*** | | | | | | | | | | | | |
| ARS4/6 | 22.2 | 178 | Deep inner | 11.99 |  | 4.93 |  | 181 |  | 328 |  | 0.09 |
| ARS4/2 | 2.6 | 123 | Shallow inner | 1.14 |  | 1.20 |  | 941 |  | 255 |  | 0.14 |
| ARS4/1 | 1.6 | 115 | Shallow inner | 1.12 |  | 1.29 |  | 1241 |  | 213 |  | 0.09 |
|  |  |  |  |  |  |  |  |  |  |  |  |  |
| **Farm Ababis (Brak section), 23°58'16.98"S, 16°08'06.48"E** | | | | | | | | | | | | |
| ***Kuibis Subgroup, Dabis Formation, Kanies Member*** | | | | | | | | | | | | |
| BRK2/12 | 65 | 97 | Outer | 2.40 |  | 1.48 |  | 795 |  | 136 |  | 0.10 |
| BRK2/11 | 60 | 85 | Outer | 9.79 |  | 5.03 |  | 187 |  | 116 |  | 0.11 |
| PACS2a | Standard |  |  | 6.52 | 1.89 | 4.05 | 1.27 | 430 | 3.40 | 990 | 0.41 |  |
| PACS2b | Standard |  |  | 6.50 | 1.89 | 4.07 | 1.27 | 433 | 3.33 | 987 | 0.42 |  |
| PACS2c | Standard |  |  |  |  | 4.05 |  |  |  |  |  |  |
| PACS2d | Standard |  |  |  |  | 4.06 |  |  |  |  |  |  |

**Table S4:** Fe speciation data

| **Sample ID** | **Section height (m)** | **Ramp position** | **Fecarb (wt%)** | **Feox (wt%)** | **Femag (wt%)** | **Fepy (wt%)** | **FeHR/FeT** | **Fepy/FeHR** |
| --- | --- | --- | --- | --- | --- | --- | --- | --- |
| **Farm Swartpunt, 27°28'25.98"S, 16°41'45.12"E** (data published in ref 14) | | | | | | | | |
| ***Schwarzrand Subgroup, Urusis Formation, Spitskop Member*** | | | | | | | | |
| SWP2/12 | 87.24 | Outer | 0.096 | 0.325 | 0.125 | 0.001 | 0.133 | 0.002 |
| SWP2/10 | 78.44 | Outer | 0.117 | 0.278 | 0.180 | 0.002 | 0.186 | 0.004 |
| SWP2/8 | 74.74 | Outer | 0.116 | 0.116 | 0.156 | 0.001 | 0.072 | 0.003 |
| SWP2/7 | 72.64 | Outer | 0.152 | 0.084 | 0.130 | 0.001 | 0.073 | 0.002 |
| SWP2/6 | 70.24 | Outer | 0.148 | 0.082 | 0.155 | 0.003 | 0.070 | 0.008 |
| SWP2/5 | 69.04 | Outer | 0.176 | 0.082 | 0.165 | 0.001 | 0.094 | 0.001 |
|  |  |  |  |  |  |  |  |  |
| **Farm Swartkloofberg (Pinnacle Reefs locality), 27°26'48.24"S, 16°33'43.62"E** (data published in ref 14) | | | | | | | | |
| ***Schwarzrand Subgroup, Urusis Formation, Spitskop Member*** | | | | | | | | |
| SK1/10 | 89 | Outer | 0.180 | 0.112 | 0.152 | 0.002 | 0.085 | 0.005 |
| SK1/9 | 75 | Outer | 0.146 | 0.252 | 0.141 | 0.006 | 0.131 | 0.012 |
| ***Schwarzrand Subgroup, Urusis Formation, Feldschuhhorn Member*** | | | | | | | | |
| SK1/8 | 47.75 | Mid | 0.146 | 0.067 | 0.154 | 0.003 | 0.070 | 0.008 |
| SK1/7 | 44.55 | Mid | 0.146 | 0.079 | 0.156 | 0.001 | 0.071 | 0.003 |
| SK1/6 | 34.95 | Mid | 0.159 | 0.076 | 0.168 | 0.001 | 0.077 | 0.003 |
| SK1/5 | 23.75 | Mid | 0.285 | 0.116 | 0.277 | 0.006 | 0.147 | 0.009 |
| SK1/4 | 15.75 | Mid | 0.050 | 0.636 | 0.133 | 0.000 | 0.182 | 0.000 |
| SK1/3 | 10.15 | Mid | 0.059 | 0.051 | 0.096 | 0.001 | 0.043 | 0.003 |
| SK1/1 | 1.5 | Mid | 0.145 | 0.092 | 0.119 | 0.002 | 0.092 | 0.007 |
|  |  |  |  |  |  |  |  |  |
|  |  |  |  |  |  |  |  |  |
| **Sample ID** | **Section height (m)** | **Ramp position** | **Fecarb (wt%)** | **Feox (wt%)** | **Femag (wt%)** | **Fepy (wt%)** | **FeHR/FeT** | **Fepy/FeHR** |
| **Farm Kliphoek, 27°17'05.28"S, 16°46'47.58"E** | | | | | | | | |
| ***Schwarzrand Subgroup, Nudaus Formation, Vingerbreek Member*.** Samples in red denote ironstones (data not included in the compilation) | | | | | | | | |
| NUD-20 | 214 | Mid | 0.147 | 0.775 | 0.216 | 0.001 | 0.206 | 0.001 |
| NUD-19 | 188 | Mid | 0.021 | 17.326 | 0.249 | 0.003 | 0.913 | 0.000 |
| NUD-18 | 178 | Mid | 0.018 | 21.417 | 0.466 | 0.000 | 0.891 | 0.000 |
| NUD-16 | 154 | Mid | 0.012 | 16.175 | 0.713 | 0.001 | 0.765 | 0.000 |
| NUD-15 | 144 | Mid | 0.028 | 4.397 | 0.238 | 0.002 | 0.667 | 0.000 |
| NUD-14 | 134 | Mid | 0.024 | 14.675 | 0.303 | 0.002 | 0.855 | 0.000 |
| NUD-12 | 114 | Mid | 0.014 | 17.939 | 0.762 | 0.000 | 0.747 | 0.000 |
| NUD-10 | 94 | Mid | 0.016 | 22.396 | 0.879 | 0.001 | 0.809 | 0.000 |
| NUD-8 | 74 | Mid | 0.0153 | 0.562 | 0.096 | 0.001 | 0.136 | 0.001 |
| NUD-7 | 64 | Mid | 0.085 | 1.838 | 0.094 | 0.001 | 0.318 | 0.000 |
| NUD-5 | 43 | Mid | 0.012 | 15.753 | 0.742 | 0.002 | 0.554 | 0.000 |
| NUD-4 | 31 | Mid | 0.194 | 0.156 | 0.125 | 0.001 | 0.096 | 0.002 |
| NUD-3 | 24 | Mid | 0.109 | 0.341 | 0.068 | 0.000 | 0.102 | 0.001 |
| NUD-2 | 21 | Mid | 0.163 | 3.609 | 0.180 | 0.002 | 0.484 | 0.000 |
| NUD-1 | 10 | Mid | 0.138 | 5.088 | 0.183 | 0.001 | 0.575 | 0.000 |
|  |  |  |  |  |  |  |  |  |
|  |  |  |  |  |  |  |  |  |
|  |  |  |  |  |  |  |  |  |
|  |  |  |  |  |  |  |  |  |
|  |  |  |  |  |  |  |  |  |
|  |  |  |  |  |  |  |  |  |
| **Sample ID** | **Rescaled total Nama height (m)** | **Ramp position** | **Fecarb (wt%)** | **Feox (wt%)** | **Femag (wt%)** | **Fepy (wt%)** | **FeHR/FeT** | **Fepy/FeHR** |
| **Road transect D850** | | | | | | | | |
| ***Schwarzrand Subgroup, Nomtsas Formation, Niep Member*** | | | | | | | | |
| S15 | 2080.27 | Shallow inner | 0.097 | 0.713 | 0.148 | 0.001 | 0.300 | 0.001 |
| S14 | 2055.88 | Shallow inner | 0.076 | 0.517 | 0.116 | 0.000 | 0.284 | 0.000 |
| ***Schwarzrand Subgroup, Urusis Formation, undefined member*** | | | | | | | | |
| S13 | 1956.31 | Shallow inner | 0.070 | 0.483 | 0.077 | 0.001 | 0.262 | 0.001 |
| D850-1 | 1852.67 | Shallow inner | 0.087 | 0.906 | 0.152 | 0.001 | 0.295 | 0.001 |
| S12a | 1775.45 | Shallow inner | 0.085 | 0.286 | 0.075 | 0.000 | 0.271 | 0.001 |
| S12b | 1775.45 | Shallow inner | 0.112 | 1.063 | 0.219 | 0.001 | 0.415 | 0.001 |
| D850-2 | 1584.44 | Shallow inner | 0.266 | 1.380 | 0.486 | 0.000 | 0.363 | 0.000 |
| S11 | 1492.99 | Shallow inner | 0.062 | 0.491 | 0.070 | 0.000 | 0.303 | 0.000 |
| D850-4 | 1316.20 | Shallow inner | 0.092 | 0.714 | 0.105 | 0.001 | 0.289 | 0.001 |
| D850-6 | 1142.46 | Shallow inner | 0.202 | 1.046 | 0.343 | 0.001 | 0.418 | 0.001 |
| S10 | 1051.02 | Shallow inner | 0.154 | 0.841 | 0.271 | 0.001 | 0.223 | 0.001 |
| ***Schwarzrand Subgroup, Nudaus Formation, Vingerbreek Member*** | | | | | | | | |
| S9 | 965 | Shallow inner | 0.168 | 0.940 | 0.248 | 0.001 | 0.360 | 0.001 |
| S8 | 930 | Shallow inner | 0.110 | 0.604 | 0.133 | 0.001 | 0.222 | 0.001 |
| S7 | 900 | Shallow inner | 0.232 | 0.555 | 0.187 | 0.001 | 0.327 | 0.001 |
| S6 | 870 | Shallow inner | 0.299 | 0.644 | 0.271 | 0.000 | 0.387 | 0.000 |
| ***Schwarzrand Subgroup, Nudaus Formation, Niederhagen Member*** | | | | | | | | |
| S5 | 865 | Deep inner | 0.213 | 1.224 | 0.244 | 0.000 | 0.457 | 0.000 |
| S4 | 780 | Deep inner | 0.289 | 0.676 | 0.253 | 0.002 | 0.392 | 0.001 |
| ***Kuibis Subgroup, Zaris Formation, Urikos Member*** | | | | | | | | |
| S3 | 760 | Mid | 0.256 | 0.879 | 0.279 | 0.000 | 0.380 | 0.000 |
| **Sample ID** | **Rescaled total Nama height (m)** | **Ramp position** | **Fecarb (wt%)** | **Feox (wt%)** | **Femag (wt%)** | **Fepy (wt%)** | **FeHR/FeT** | **Fepy/FeHR** |
| S2a | 680 | Mid | 1.018 | 0.966 | 0.452 | 0.000 | 0.685 | 0.000 |
| S2b | 680 | Mid | 0.645 | 0.732 | 0.432 | 0.000 | 0.271 | 0.000 |
| S1 | 660 | Mid | 0.202 | 0.555 | 0.187 | 0.001 | 0.146 | 0.001 |
|  |  |  |  |  |  |  |  |  |
| **Road transect C14** | | | | | | | | |
| ***Schwarzrand Subgroup, Nomtsas Formation, Niep Member*** | | | | | | | | |
| D860-21 | 2080.27 | Shallow inner | 0.073 | 1.195 | 0.152 | 0.001 | 0.272 | 0.001 |
| D860-20 | 2045.72 | Shallow inner | 0.078 | 0.558 | 0.143 | 0.000 | 0.217 | 0.000 |
| D860-19 | 2028.45 | Shallow inner | 0.079 | 0.744 | 0.246 | 0.001 | 0.240 | 0.001 |
| ***Schwarzrand Subgroup, Nomtsas Formation, Kreyrivier Member*** | | | | | | | | |
| D860-18 | 2022.35 | Shallow inner | 0.147 | 0.309 | 0.165 | 0.001 | 0.211 | 0.001 |
|  |  |  |  |  |  |  |  |  |
| **Road transect D860** | | | | | | | | |
| ***Schwarzrand Subgroup, Urusis Formation, undefined member*** | | | | | | | | |
| D860-16 | 1451.34 | Shallow inner | 0.074 | 0.950 | 0.153 | 0.001 | 0.265 | 0.001 |
| D860-15 | 1374.12 | Shallow inner | 0.098 | 1.318 | 0.280 | 0.000 | 0.329 | 0.000 |
| D860-14 | 1344.65 | Shallow inner | 0.034 | 1.097 | 0.123 | 0.001 | 0.234 | 0.001 |
| D860-10 | 1193.26 | Shallow inner | 0.081 | 0.574 | 0.126 | 0.000 | 0.225 | 0.000 |
| D860-9 | 1157.70 | Shallow inner | 0.076 | 0.475 | 0.129 | 0.000 | 0.139 | 0.000 |
| D860-8 | 1112.99 | Shallow inner | 0.096 | 1.131 | 0.157 | 0.001 | 0.279 | 0.001 |
| D860-7 | 1080.48 | Shallow inner | 0.135 | 1.092 | 0.322 | 0.000 | 0.298 | 0.000 |
| D860-6 | 1059.14 | Shallow inner | 0.128 | 0.902 | 0.135 | 0.001 | 0.336 | 0.001 |
|  |  |  |  |  |  |  |  |  |
| **Sample ID** | **Rescaled total Nama height (m)** | **Ramp position** | **Fecarb (wt%)** | **Feox (wt%)** | **Femag (wt%)** | **Fepy (wt%)** | **FeHR/FeT** | **Fepy/FeHR** |
| ***Schwarzrand Subgroup, Nudaus Formation, Vingerbreek Member*** | | | | | | | | |
| D860-5 | 950 | Shallow inner | 0.085 | 0.864 | 0.133 | 0.000 | 0.327 | 0.000 |
| D860-4 | 890 | Shallow inner | 0.121 | 1.059 | 0.179 | 0.000 | 0.307 | 0.000 |
| ***Schwarzrand Subgroup, Nudaus Formation, Niederhagen Member*** | | | | | | | | |
| D860-3 | 860 | Deep inner | 0.059 | 0.718 | 0.062 | 0.000 | 0.241 | 0.000 |
| D860-2 | 840 | Deep inner | 0.084 | 0.837 | 0.085 | 0.001 | 0.391 | 0.001 |
| D860-1 | 780 | Deep inner | 0.066 | 0.668 | 0.102 | 0.000 | 0.287 | 0.000 |
|  |  |  |  |  |  |  |  |  |
|  |  |  |  |  |  |  |  |  |
|  |  |  |  |  |  |  |  |  |
|  |  |  |  |  |  |  |  |  |
|  |  |  |  |  |  |  |  |  |
|  |  |  |  |  |  |  |  |  |
|  |  |  |  |  |  |  |  |  |
|  |  |  |  |  |  |  |  |  |
|  |  |  |  |  |  |  |  |  |
|  |  |  |  |  |  |  |  |  |
|  |  |  |  |  |  |  |  |  |
|  |  |  |  |  |  |  |  |  |
|  |  |  |  |  |  |  |  |  |
|  |  |  |  |  |  |  |  |  |
|  |  |  |  |  |  |  |  |  |
|  |  |  |  |  |  |  |  |  |
|  |  |  |  |  |  |  |  |  |
| **Sample ID** | **Section height (m)** | **Ramp position** | **Fecarb (wt%)** | **Feox (wt%)** | **Femag (wt%)** | **Fepy (wt%)** | **FeHR/FeT** | **Fepy/FeHR** |
| **Farm Swartmodder, 24°53'40.98"S, 16°19'31.02"E** (data published in ref 14) | | | | | | | | |
| **Kuibis Subgroup, Dabis Formation, Kanies Member** | | | | | | | | |
| SWM2/3 | 15.5 | Shallow inner |  |  |  |  | 0.130 | 0.000 |
| SWM2/1 | 3 | Shallow inner |  |  |  |  | 0.190 | 0.000 |
|  |  |  |  |  |  |  |  |  |
| **Farm Omkyk, 24°48'19.02"S, 16°13'45.00"E** (data published in ref 14) | | | | | | | | |
| ***Kuibis Subgroup, Zaris Formation, Omkyk Member*** | | | | | | | | |
| OMK4/1 | 73 | Deep inner |  |  |  |  | 0.860 | 0.000 |
| OMK3/14 | 44 | Mid |  |  |  |  | 0.800 | 0.000 |
| OMK3/3 | 4.5 | Mid |  |  |  |  | 0.690 | 0.000 |
| OMK3/2 | 3 | Mid |  |  |  |  | 0.600 | 0.046 |
|  | | | | | | | | |
| **Farm Driedoornvlakte, 23°51.429'S 16°39.637'E** | | | | | | | | |
| ***Schwarzrand Subgroup, Nudaus Formation, Niederhagen Member*** | | | | | | | | |
| DV2-23 | 660 | Mid | 0.110 | 1.089 | 0.278 | 0.001 | 0.332 | 0.000 |
| DV2-24 | 640 | Mid | 0.048 | 1.110 | 0.139 | 0.001 | 0.321 | 0.000 |
| DV2-24b | Replicate |  | 0.053 | 1.068 | 0.130 |  |  |  |
| DV2-24c | Replicate |  | 0.054 | 1.045 | 0.130 |  |  |  |
| DV2-24d | Replicate |  | 0.053 | 1.061 | 0.139 |  |  |  |
| DV2-24e | Replicate |  | 0.051 | 1.034 | 0.138 |  |  |  |
| DV2-25 | 624 | Mid | 0.045 | 1.111 | 0.160 | 0.000 | 0.332 | 0.000 |
| DV2-26 | 622 | Mid | 0.118 | 1.388 | 0.263 | 0.001 | 0.373 | 0.000 |
| DV2-27 | 615 | Mid | 0.051 | 1.146 | 0.151 | 0.001 | 0.272 | 0.000 |
|  | | | | | | | | |
| **Sample ID** | **Section height (m)** | **Ramp position** | **Fecarb (wt%)** | **Feox (wt%)** | **Femag (wt%)** | **Fepy (wt%)** | **FeHR/FeT** | **Fepy/FeHR** |
| ***Kuibis Subgroup, Zaris Formation, Urikos Member (above OS2 Unit 3m)*** | | | | | | | | |
| DV2-28 | 580 | Mid | 0.047 | 0.772 | 0.112 | 0.000 | 0.254 | 0.000 |
| DV2-30 | 534 | Mid | 0.081 | 0.079 | 0.083 | 0.000 | 0.060 | 0.000 |
| DV2-29 | 532 | Mid | 0.098 | 0.237 | 0.090 | 0.000 | 0.106 | 0.001 |
| DV2-22 | 557 | Mid | 0.135 | 0.586 | 0.166 | 0.000 | 0.215 | 0.000 |
| DV2-21 | 555 | Mid | 0.174 | 0.370 | 0.168 | 0.003 | 0.199 | 0.004 |
| DV5 | 550 | Mid | 0.175 | 0.618 | 0.199 | 0.001 | 0.165 | 0.001 |
| DV4 | 548 | Mid | 0.141 | 0.362 | 0.117 | 0.003 | 0.170 | 0.005 |
| DV3 | 538 | Mid | 0.252 | 0.585 | 0.244 | 0.001 | 0.254 | 0.001 |
| DV2 | 530 | Mid | 0.125 | 0.186 | 0.078 | 0.000 | 0.196 | 0.000 |
| DV1 | 528 | Mid | 0.157 | 0.231 | 0.104 | 0.000 | 0.153 | 0.000 |
| DV2-31 | 510 | Mid | 0.065 | 0.204 | 0.054 | 0.000 | 0.084 | 0.000 |
| ***Kuibis Subgroup, Zaris Formation, Urikos Member (down-dip equivalent to OS2 Unit 3m)*** | | | | | | | | |
| DV2-20 | 470 | Mid | 0.161 | 0.797 | 0.203 | 0.000 | 0.213 | 0.000 |
| DV2-19 | 460 | Mid | 0.169 | 0.238 | 0.185 | 0.000 | 0.104 | 0.001 |
| ***Kuibis Subgroup, Zaris Formation, Urikos Member (down-dip equivalent to OS2 Unit 3i)*** | | | | | | | | |
| DV2-18 | 450 | Mid | 0.087 | 1.467 | 0.136 | 0.000 | 0.325 | 0.000 |
| DV2-17 | 425 | Mid | 0.031 | 1.429 | 0.188 | 0.000 | 0.309 | 0.000 |
| ***Kuibis Subgroup, Zaris Formation, Urikos Member (down-dip equivalent to OS2 Unit 2m)*** | | | | | | | | |
| DV2-15 | 310 | Mid | 0.093 | 0.630 | 0.078 | 0.002 | 0.157 | 0.002 |
| DV2-16 | 310 | Mid | 0.137 | 0.321 | 0.090 | 0.001 | 0.117 | 0.001 |
| ***Kuibis Subgroup, Zaris Formation, Urikos Member (down-dip equivalent to OS2 Unit 1b)*** | | | | | | | | |
| DV2-8 | 230 | Mid | 0.100 | 0.100 | 0.085 | 0.004 | 0.063 | 0.015 |
| DV2-14 | 230 | Mid | 0.172 | 0.201 | 0.102 | 0.001 | 0.102 | 0.001 |
| **Sample ID** | **Section height (m)** | **Ramp position** | **Fecarb (wt%)** | **Feox (wt%)** | **Femag (wt%)** | **Fepy (wt%)** | **FeHR/FeT** | **Fepy/FeHR** |
| DV2-13 | 185 | Mid | 0.155 | 0.208 | 0.104 | 0.000 | 0.104 | 0.001 |
| DV2-7 | 179 | Mid | 0.087 | 0.997 | 0.084 | 0.001 | 0.219 | 0.001 |
| DV2-6 | 172 | Mid | 0.100 | 0.259 | 0.065 | 0.001 | 0.086 | 0.002 |
| DV2-12 | 170 | Mid | 0.175 | 0.151 | 0.122 | 0.001 | 0.095 | 0.002 |
| ***Kuibis Subgroup, Zaris Formation, Urikos Member (down-dip equivalent to OS2 Unit 1a)*** | | | | | | | | |
| DV2-11 | 163 | Mid | 0.258 | 0.318 | 0.201 | 0.001 | 0.159 | 0.001 |
| DV2-9 | 120 | Mid | 0.040 | 2.092 | 0.053 | 0.004 | 0.471 | 0.002 |
| DV2-10 | 120 | Mid | 0.119 | 0.288 | 0.067 | 0.014 | 0.118 | 0.029 |
|  |  |  |  |  |  |  |  |  |
| **Farm Arasab, 26°57'48.54"S, 16°27'25.98"E** (data published in ref 14) | | | | | | | | |
| ***Kuibis Subgroup, Dabis Formation, Mara Member*** | | | | | | | | |
| ARS4/6 | 22.2 | Deep inner | 0.000 | 2.912 | 0.160 | 0.001 | 0.623 | 0.000 |
| ARS4/2 | 2.6 | Shallow inner | 0.161 | 0.564 | 0.025 | 0.003 | 0.628 | 0.004 |
| ARS4/1 | 1.6 | Shallow inner | 0.063 | 0.926 | 0.027 | 0.003 | 0.788 | 0.003 |
|  |  |  |  |  |  |  |  |  |
| **Farm Ababis (Brak section), 23°58'16.98"S, 16°08'06.48"E** (data published in ref 14) | | | | | | | | |
| ***Kuibis Subgroup, Dabis Formation, Kanies Member*** | | | | | | | | |
| BRK2/12 | 65 | Outer |  |  |  |  | 0.890 | 0.080 |
| BRK2/11 | 60 | Outer |  |  |  |  | 0.630 | 0.000 |
|  |  |  |  |  |  |  |  |  |
| **In house standard** | |  |  |  |  |  |  |  |
| C165 |  |  | 0.847 | 0.324 | 0.858 |  |  |  |
| C165 |  |  | 0.868 | 0.331 | 0.887 |  |  |  |
|  |  | Long-term: | 0.84±0.16 | 0.33±0.04 | 0.80±0.11 |  |  |  |

**Table S5:** P speciation data

| **Sample ID** | **Section height (m)** | **Ramp position** | **PFe1 (wt%)** | **Pauth (wt%)** | **Pmag (wt%)** | **PFe2 (wt%)** | **Pdet (wt%)** | **Porg (wt%)** | **Preac (wt%)** |  |
| --- | --- | --- | --- | --- | --- | --- | --- | --- | --- | --- |
| **Farm Swartpunt, 27°28'25.98"S, 16°41'45.12"E** | | | | | | | | | | |
| ***Schwarzrand Subgroup, Urusis Formation, Spitskop Member*** | | | | | | | | | | |
| SWP2/12 | 87.24 | Outer | 0.001 | 0.034 | 0.001 | 0.000 | 0.043 | 0.001 | 0.037 |  |
| SWP2/10 | 78.44 | Outer | 0.001 | 0.039 | 0.001 | 0.003 | 0.022 | 0.001 | 0.044 |  |
| SWP2/8 | 74.74 | Outer | 0.000 | 0.065 | 0.001 | 0.000 | 0.030 | 0.001 | 0.067 |  |
| SWP2/7 | 72.64 | Outer | 0.001 | 0.038 | 0.001 | 0.000 | 0.036 | 0.001 | 0.040 |  |
| SWP2/6 | 70.24 | Outer | 0.000 | 0.036 | 0.001 | 0.000 | 0.031 | 0.001 | 0.038 |  |
| SWP2/5 | 69.04 | Outer | 0.001 | 0.035 | 0.001 | 0.000 | 0.028 | 0.002 | 0.038 |  |
|  |  |  |  |  |  |  |  |  |  |  |
| **Farm Swartkloofberg (Pinnacle Reefs locality), 27°26'48.24"S, 16°33'43.62"E** | | | | | | | | | | |
| ***Schwarzrand Subgroup, Urusis Formation, Spitskop Member*** | | | | | | | | | | |
| SK1/10 | 89 | Outer | 0.001 | 0.034 | 0.001 | 0.000 | 0.038 | 0.001 | 0.036 |  |
| SK1/9 | 75 | Outer | 0.001 | 0.036 | 0.001 | 0.000 | 0.038 | 0.001 | 0.039 |  |
| ***Schwarzrand Subgroup, Urusis Formation, Feldschuhhorn Member*** | | | | | | | | | | |
| SK1/8 | 47.75 | Mid | 0.000 | 0.037 | 0.001 | 0.000 | 0.032 | 0.001 | 0.039 |  |
| SK1/7 | 44.55 | Mid | 0.000 | 0.032 | 0.001 | 0.000 | 0.038 | 0.001 | 0.035 |  |
| SK1/6 | 34.95 | Mid | 0.001 | 0.035 | 0.001 | 0.000 | 0.034 | 0.001 | 0.039 |  |
| SK1/5 | 23.75 | Mid | 0.001 | 0.034 | 0.001 | 0.000 | 0.037 | 0.001 | 0.037 |  |
| SK1/4 | 15.75 | Mid | 0.016 | 0.040 | 0.001 | 0.000 | 0.008 | 0.002 | 0.058 |  |
| SK1/3 | 10.15 | Mid | 0.000 | 0.028 | 0.000 | 0.000 | 0.012 | 0.001 | 0.029 |  |
| SK1/1 | 1.5 | Mid | 0.000 | 0.028 | 0.001 | 0.000 | 0.027 | 0.001 | 0.030 |  |
|  |  |  |  |  |  |  |  |  |  |  |
| **Sample ID** | **Section/total height (m)** | **Ramp position** | **PFe1 (wt%)** | **Pauth (wt%)** | **Pmag (wt%)** | **PFe2 (wt%)** | **Pdet (wt%)** | **Porg (wt%)** | **Preac (wt%)** |  |
| **Farm Kliphoek, 27°17'05.28"S, 16°46'47.58"E** | | | | | | | | | | |
| ***Schwarzrand Subgroup, Nudaus Formation, Vingerbreek Member*** | | | | | | | | | | |
| NUD-4 | 31 | Mid | 0.001 | 0.051 | 0.001 | 0.000 | 0.027 | 0.001 | 0.054 |  |
| NUD-3 | 24 | Mid | 0.001 | 0.026 | 0.000 | 0.000 | 0.009 | 0.001 | 0.029 |  |
| NUD-1 | 10 | Mid | 0.008 | 0.021 | 0.000 | 0.000 | 0.003 | 0.016 | 0.047 |  |
|  |  |  |  |  |  |  |  |  |  |  |
| **Road transect D850** | | | | | | | | | | |
| ***Schwarzrand Subgroup, Urusis Formation, undefined member*** | | | | | | | | | | |
| S12b | 1775.45 | Shallow inner | 0.002 | 0.055 | 0.002 | 0.000 | 0.054 | 0.001 | 0.060 |  |
| ***Schwarzrand Subgroup, Nudaus Formation, Niederhagen Member*** | | | | | | | | | | |
| S5 | 865 | Deep inner | 0.002 | 0.017 | 0.001 | 0.000 | 0.005 | 0.002 | 0.021 |  |
| ***Kuibis Subgroup, Zaris Formation, Urikos Member*** | | | | | | | | | | |
| S1 | 660 | Mid | 0.000 | 0.019 | 0.000 | 0.000 | 0.006 | 0.002 | 0.022 |  |
|  |  |  |  |  |  |  |  |  |  |  |
| **Road transect C14** | | | | | | | | | | |
| ***Schwarzrand Subgroup, Nomtsas Formation, Niep Member*** | | | | | | | | | | |
| D860-20 | 2045.72 | Shallow inner | 0.000 | 0.061 | 0.001 | 0.000 | 0.031 | 0.001 | 0.063 |  |
| ***Schwarzrand Subgroup, Nomtsas Formation, Kreyrivier Member*** | | | | | | | | | | |
| D860-18 | 2022.35 | Shallow inner | 0.002 | 0.067 | 0.001 | 0.000 | 0.040 | 0.001 | 0.071 |  |
|  |  |  |  |  |  |  |  |  |  |  |
| **Road transect D860** | | | | | | | | | | |
| ***Schwarzrand Subgroup, Urusis Formation, undefined member*** | | | | | | | | | | |
| D860-9 | 1157.70 | Shallow inner | 0.001 | 0.051 | 0.001 | 0.001 | 0.022 | 0.001 | 0.055 |  |
| **Sample ID** | **Section height (m)** | **Ramp position** | **PFe1 (wt%)** | **Pauth (wt%)** | **Pmag (wt%)** | **PFe2 (wt%)** | **Pdet (wt%)** | **Porg (wt%)** | **Preac (wt%)** |  |
| **Farm Swartmodder, 24°53'40.98"S, 16°19'31.02"E** | | | | | | | | | | |
| **Kuibis Subgroup, Dabis Formation, Kanies Member** | | | | | | | | | | |
| SWM2/3 | 15.5 | Shallow inner | 0.001 | 0.005 | 0.000 | 0.001 | 0.001 | 0.000 | 0.007 |  |
| SWM2/1 | 3 | Shallow inner | 0.000 | 0.027 | 0.001 | 0.000 | 0.048 | 0.001 | 0.029 |  |
|  |  |  |  |  |  |  |  |  |  |  |
| **Farm Omkyk, 24°48'19.02"S, 16°13'45.00"E** | | | | | | | | | | |
| ***Kuibis Subgroup, Zaris Formation, Omkyk Member*** | | | | | | | | | | |
| OMK4/1 | 73 | Deep inner | 0.005 | 0.001 | 0.000 | 0.000 | 0.001 | 0.002 | 0.009 |  |
| OMK3/14 | 44 | Mid | 0.002 | 0.075 | 0.001 | 0.000 | 0.024 | 0.001 | 0.079 |  |
| OMK3/3 | 4.5 | Mid | 0.002 | 0.000 | 0.000 | 0.001 | 0.001 | 0.002 | 0.005 |  |
| OMK3/2 | 3 | Mid | 0.001 | 0.001 | 0.000 | 0.004 | 0.000 | 0.007 | 0.012 |  |
|  | | | | | | | | | | |
| **Farm Driedoornvlakte, 23°51.429'S 16°39.637'E** | | | | | | | | | | |
| ***Kuibis Subgroup, Zaris Formation, Urikos Member (above OS2 Unit 3m)*** | | | | | | | | | | |
| DV2-21 | 555 | Mid | 0.001 | 0.030 | 0.001 | 0.000 | 0.009 | 0.003 | 0.034 |  |
| ***Kuibis Subgroup, Zaris Formation, Urikos Member (down-dip equivalent to OS2 Unit 3m)*** | | | | | | | | | | |
| DV2-20 | 470 | Mid | 0.001 | 0.031 | 0.001 | 0.000 | 0.009 | 0.002 | 0.035 |  |
| DV2-19 | 460 | Mid | 0.000 | 0.047 | 0.001 | 0.001 | 0.015 | 0.001 | 0.050 |  |
|  |  |  |  |  |  |  |  |  |  |  |
| **Farm Arasab, 26°57'48.54"S, 16°27'25.98"E** | | | | | | | | | | |
| ***Kuibis Subgroup, Dabis Formation, Mara Member*** | | | | | | | | | | |
| ARS4/6 | 22.2 | Deep inner | 0.007 | 0.015 | 0.001 | 0.001 | 0.006 | 0.001 | 0.024 |  |
| ARS4/2 | 2.6 | Shallow inner | 0.002 | 0.007 | 0.000 | 0.000 | 0.015 | 0.000 | 0.010 |  |
| **Sample ID** | **Section height (m)** | **Ramp position** | **PFe1 (wt%)** | **Pauth (wt%)** | **Pmag (wt%)** | **PFe2 (wt%)** | **Pdet (wt%)** | **Porg (wt%)** | **Preac (wt%)** |  |
| ARS4/1 | 1.6 | Shallow inner | 0.002 | 0.009 | 0.000 | 0.000 | 0.009 | 0.000 | 0.011 |  |
|  |  |  |  |  |  |  |  |  |  |  |
| **Farm Ababis (Brak section), 23°58'16.98"S, 16°08'06.48"E** | | | | | | | | | | |
| ***Kuibis Subgroup, Dabis Formation, Kanies Member*** | | | | | | | | | | |
| BRK2/12 | 65 | Outer | 0.001 | 0.003 | 0.000 | 0.000 | 0.010 | 0.000 | 0.006 |  |
| BRK2/11 | 60 | Outer | 0.001 | 0.008 | 0.000 | 0.000 | 0.003 | 0.001 | 0.011 |  |
|  |  |  |  |  |  |  |  |  |  |  |
| **Reproducibility** | |  |  |  |  |  |  |  |  |  |
|  |  |  | **PFe1** | **Pauth** | **Pmag** | **PFe2** | **Pdet** | **Porg** | **Summed P** |  |
| **%RSD** |  |  | 3.2 | 5.3 | 7.3 | 2.9 | 2.7 | 8.1 | 1.8 |  |
